# Identification of QTLs and a candidate gene affecting rice grain volume via high-density genetic mapping

**DOI:** 10.3389/fpls.2025.1579589

**Published:** 2025-03-31

**Authors:** Zhiguang Sun, Hongzhou An, Zeyu Qiu, Jingfang Li, Jian Li, Bo Yang, Jinbo Liu, Tingmu Chen, Yuqin Zhang, Baiguan Lu, Yan Liu, Baoxiang Wang, Dayong Xu

**Affiliations:** ^1^ Institute of Rice Research, Lianyungang Academy of Agricultural Sciences, Lianyungang, China; ^2^ The Key Laboratory of Crop Genetics and Breeding of Hebei Province, Institute of Cereal and Oil Crops, Hebei Academy of Agriculture and Forestry Sciences, Shijiazhuang, China; ^3^ Institute of Crop Germplasm and Biotechnology, Provincial Key Laboratory of Agrobiology, Jiangsu Academy of Agricultural Sciences, Nanjing, China

**Keywords:** weedy rice, grain volume, QTL, high-density genetic map, ethylene receptor

## Abstract

**Introduction:**

Grain volume is a key agronomic trait of rice. It is coordinately determined by grain length, width, thickness, and roundness, which influences the rice yield and quality, yet the molecular mechanism is still not fully understood.

**Methods:**

In this study, a mapping population of Ludao (weedy rice) and Guangbaixiangzhan (GBXZ) was developed in Lianyungang, Jiangsu province, China, and was employed to construct a high-density genetic map by use of the RICE 1 K mGPS chip in 2021. The mapping of QTLs was carried out with IciMapping software using the inclusive composite interval mapping (ICIM) method.

**Results and discussion:**

A total of eight QTLs for grain volume, explained 4.22–19.75% of the total phenotypic variation, were detected with LOD scores ranging from 3.33 to 13.25. Among these loci, five are known genes or loci related to grain size, and three loci, *qGV4-2*, *qGV7-1*, and *qGV7-2*, were newly identified. The major QTL, *qGV7-2*, explained the highest phenotypic variation, was validated using NIL pairs. By combining gene functional annotation, gene expression analysis and sequence comparison within the mapping interval of *qGV7-2*, a candidate gene (*LOC_Os07g15540*), encoding an ethylene receptor, OsETR4, was identified. Further haplotype–phenotype analysis revealed this gene to be significantly associated with grain length, width, and thousand-grain weight. Thus we identified *LOC_Os07g15540* as the most likely candidate gene. Taken together, our findings provide a basis for functional research on *qGV7-2*, and broaden our understanding of role of genetic factors in regulating grain volume, thus providing an important resource for yield improvement in rice.

## 1 Introduction

Rice (*Oryza sativa* L.) is one of the most important food crops worldwide, the total production can be influenced by multiple factors such as a reduction in cultivated land area, frequency of extreme weather events, and wars, thereby posing a serious threat to global food security. Consequently, ensuring continual increases in the yield of rice has become a priority for breeders in all rice-producing countries (Liu et al., 2022; Li et al., 2022).

Grain volume reflects the “reservoir capacity” for the accumulation of photosynthetic products in rice, and is coordinately determined by grain length, width, thickness, and roundness (Hao et al., 2024). Grain volume is a complex trait controlled by multiple loci, and affected by environmental factors. To date, most studies on the genes regulating grain traits have tended to focus on grain length, width, and thickness, and 1000-grain weight. For example, Zhan et al. cloned a quantitative trait locus (QTL), *GL10*, which encodes a MADS-box family (Zhan et al., 2022), whereas Shi et al. cloned a QTL (*GW6*) regulating grain width, which encodes a GAST family protein regulated by gibberellins that increase grain width by promoting cell expansion of the spikelet hull in rice (Shi et al., 2020). Using a RIL population, Zhang et al. detected a QTL strongly associated with grain thickness on rice chromosome 9, and identified the candidate gene *Os09g0535500* based on transcriptome and RT-PCR analyses (Zhang et al., 2020). In addition, Ruan et al. cloned a semi-dominant QTL (*qTGW2*) regulating grain width and weight in rice. This gene encodes a cell number regulator, OsCNR1, which affects grain width and weight by influencing cell division and expansion in the grains (Ruan et al., 2020). However, owing to limitations with respect to measurement methods and effects, there have been few reports on genes or QTLs associated rice grain volume to date.

As a consequence of on-going advances in molecular biology techniques, millions of single-nucleotide polymorphism (SNP) markers have been discovered within the rice genome, facilitating the construction of high-quality genetic linkage maps for the detection and fine mapping of QTLs (Wei et al., 2024). Notably, all of these SNP markers are biallelic, which implies that the results obtained in different studies can be readily shared among different researchers. SNP markers have markedly refined the methods used for QTL detecting, and have been widely used in MAS breeding, GWAS analyses, and diversity analyses in recent years. For example, using a genetic map constructed by SNP markers, Wei et al. performed QTL mapping in an RIL population derived from ZP37 and R8605, and detected 39 QTLs affecting grain traits (Wei et al., 2023). These studies accordingly revealed the indispensable utility of SNP markers in QTL identification.

In this work, we performed QTL mapping of grain volume using a segregating population derived from *japonica*-type weedy rice Ludao and the *indica* rice cultivar Guangbaixiangzhan (GBXZ). The goals of this study were to (1) reliably identify novel QTLs associated with grain volume; (2) validate the effects and obtain reliable candidate gene of the major QTL-*qGV7-2*, and (3) offer molecular information to improve grain volume by pyramiding breeding. Overall, these results provide insight into the mechanisms underlying grain volume control and useful information for improvement of grain trait in rice.

## 2 Materials and methods

### 2.1 Plant materials

An F_2_ population (LG population), containing 96 plants, was generated from a cross between maternal Ludao and paternal Guangbaixiangzhan (GBXZ). Ludao is a *japonica*-type weedy rice germplasm resource unique to Lianyungang city, Jiangsu Province in eastern China, whereas GBXZ is a *indica* rice cultivar from Guangdong Province in southeastern China. The LG population and both parents were planted in the Experimental field of Lianyungang Academy of Agricultural Sciences (119°32′E, 34°56′N) in the summer of 2021, water and fertilizer were controlled using conventional management practices.

To verify the effect of the major QTL (*qGV7-2*) for grain volume, near-isogenic lines were constructed using LG43 plants, with the Ludao allele at the *qGV7-2* loci, as the donor parent, and GBXZ as the recurrent parent, by multi-generation backcrossing and selfing. In each generation, two flanking PCR markers, LG7-5 and LG7-9, were used to select the target recombinants. Finally, twelve lines carrying the *qGV7-2^LD^* or *qGV7-2^GBXZ^* alleles were selected from the BC_4_F_2_ population. The primer sequences used to amplify the target DNA sequence were as follows: LG7-5: forward primer CCGACGTGACTGGTCTGATA, reverse primer CCTCTCCCTTGTTCTGGGGTT; LG7-9: forward primer GCGTATGAGGAGTGTGGTGTGTGTGT, reverse primer TGTGTGTATAGGCCGCTTGACA. These lines for QTL validation were grown at Nanjing (118°52′E, 18°1′N) and Lianyungang in the summer of 2023.

### 2.2 Measurement of rice grain volume

At maturity, rice grains of Ludao, GBXZ, LG population, and near-isogenic lines were harvested and dried naturally. After removing empty and incomplete grains, the samples were stored at the central laboratory for later use. For the determination of grain volumes, a medical syringe was used to inject approximately 1 mL of pure water into a micro-cylinder with 0.02-mL graduations and a total volume of 2 mL, and a precise volume reading was taken. After awns had been removed from the test materials, 20 full and healthy grains were selected from each cultivar/line and placed in the previously prepared micro-cylinder, which was shaken slightly to remove bubbles adhered to the grains before reading the volume of the liquid. The volume of a single grain was then calculated based on the difference between the pre- and post-grain addition volumes and the number of grains. Each grain sample was measured three times.

### 2.3 QTL detection

The leaves of the parents and LG population plants were collected, and DNA was extracted using a column-based extraction method. After quality control was performed, the DNA samples were used for library construction, and an Agilent 2200 Bioanalyzer System was employed for library quality inspection. Thereafter, sample detection and in-depth analysis were performed using the Illumina PE150 high-throughput sequencing platform, which finally yielded 5,420 high-quality SNP loci. Using the ‘BIN’ function, SNP markers with no recombination were classified into a single bin. The presence of a QTL was determined using the ICIM method, with the LOD threshold set as 2.5, and the region in which the LOD value had declined by 1.0 was regarded as the confidence interval of the QTL (Li et al., 2007).

### 2.4 Statistical analysis

GraphPad Prism 9.0 was used for statistical analysis and generating graphs. The Correlation Plot plug-in of Origin 2021 software was used to assess the correlation between grain traits. Linkage maps were visualized using MapGene2Chromosome v2.0, and optimized using Adobe Illustrator CS6. The expression profile data of annotated genes were extracted from the RiceXPro Version 3.0 database, and the heatmaps of the annotated genes were constructed and visualized using Chiplot (https://www.chiplot.online/heatmap.html).

### 2.5 Candidate gene prediction

The genes in the QTL region were functionally annotated and analyzed by using the RGAP database (http://rice.uga.edu). The BLAST function in the *Arabidopsis* Information Resource was employed to identify the gene functions of sequences homologous to the candidate genes. The target genes were then submitted to the RiceXPro database to obtain the gene expression profile in different organs, tissues, and developmental stages in the japonica rice cultivar ‘Nipponbare’.

### 2.6 Haplotype -phenotype association analysis

The genotype function in the MBKBASE (https://www.mbkbase.org/) was employed to search for the target genes, and the genome sequences of the haplotypes were aligned with those of Ludao and GBXZ, following which, the phenotypic data of the two haplotypes were retrieved for grain length, grain width, TGW, grain roundness, grain perimeter, grain surface area, cold tolerance.

## 3 Results

### 3.1 Phenotypes of the parents and LG population

**Figure 1:**
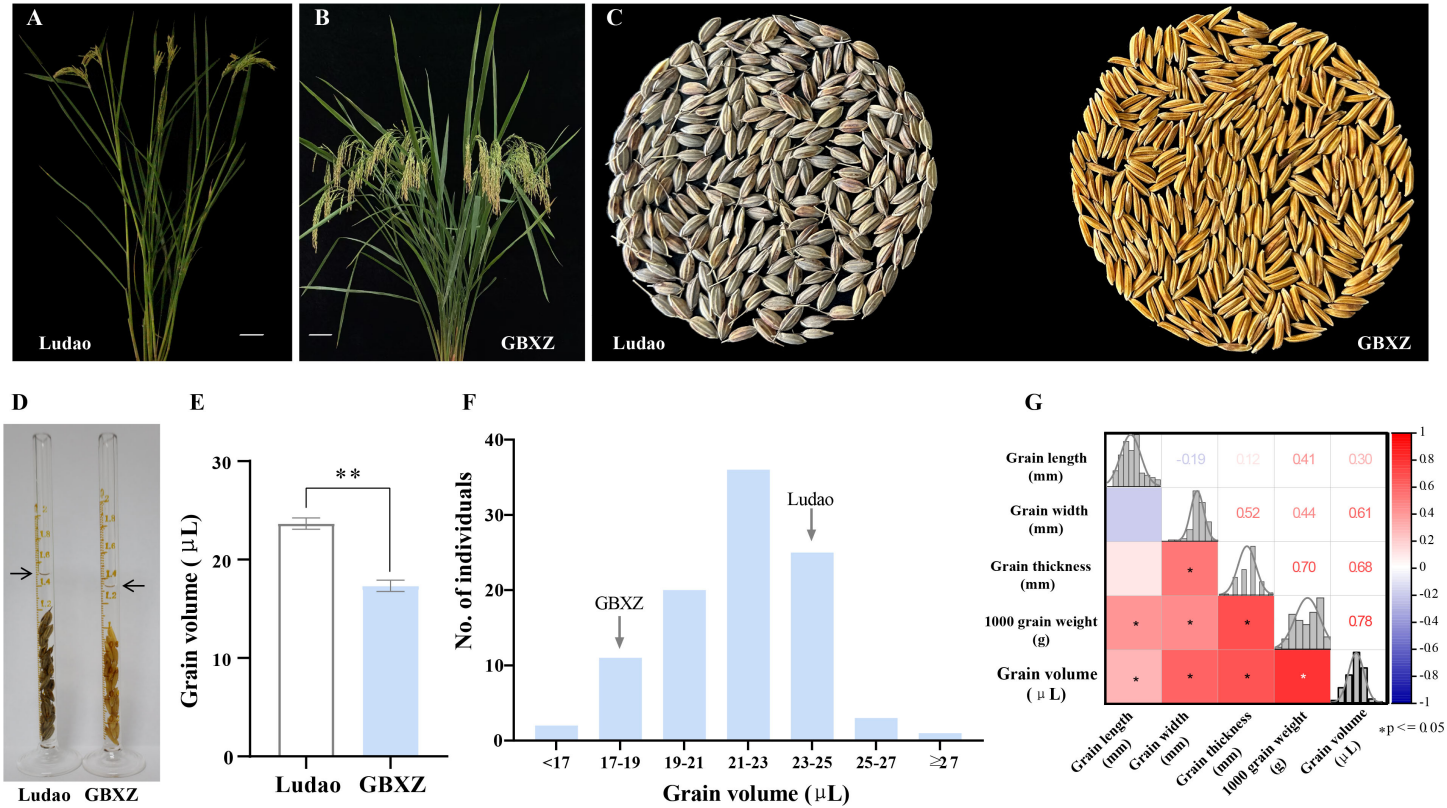
Phenotypic analysis of the parents and LG population. **(A)** Plant morphology of Ludao, scale bar = 8 cm. **(B)** Plant morphology of Guangbaixiangzhan (GBXZ), scale bar = 8 cm. **(C)** Grain phenotypes of two rice parents Ludao and GBXZ. **(D)** The measurement of grain volume of Ludao and GBXZ. Arrows indicate the liquid level after adding 20 seeds. **(E)** Comparison of the grain volume of Ludao and GBXZ. **indicate significant differences at *P* < 0.05 by Student’s *t*-test. **(F)** Distribution of grain volume in the LG population derived from Ludao and GBXZ. **(G)** Correlation analysis of grain traits in LG population. The color of the lower left square is correlated with the Pearson correlation value, and the darker the color, the higher the positive (red) or negative (blue) correlation coefficient. The number in the upper right corner represents the correlation coefficient. * indicate significant correlation at 0.05 level.

As a weedy rice germplasm, Ludao exhibiets distinguishing features including shattering grains and strong seed dormancy. It also demonstrates agronomically valuable traits such as well-developed root system and enhanced stress tolerance. Nevertheless, it possesses adverse traits, such as high plant and long awn than cultivated rice (**Figures 1A, B**), and the colors of the mature caryopsis glume and seed are black brown (**Figure 1C**). The contrasting parent, Guangbaixiangzhan (GBXZ) is a typical *indica* rice variety, which has the advantages of high quality, multiple resistance, and high yield-attributes that collectively establish it as an ideal parental line for hybrid breeding programs.

The grain volume showed significant differences (*P* < 0.01) between the parents, with the volume of Ludao grains being established to be 36.54% higher than that of GBXZ (**Figures 1D, E**). Wide variation ranging from 15.00 to 29.33 μL was observed for grain volume in LG population, with a coefficient of variation of 10.88%. Continuous segregation and significant transgressive segregation at two directions indicated that grain volume trait is controlled by multiple loci, thus grain volume in the population met the requirements for QTL mapping (**Figure 1F**).

Correlation analysis indicated that there was a high degree of correlation among grain traits in LG population. Significant correlation was observed for each pair wise combination except that between grain length and width, grain length and thickness (**Figure 1G**). Grain volume had significant positive correlations with grain length (r = 0.30), width (r = 0.61), thickness (r = 0.68) and TGW (r = 0.78), indicating that grain volume has significant impact on grain size and weight.

### 3.2 Distribution of polymorphic SNP markers and linkage map construction

LG population and the two parents were genotyped with the RICE 1 K mGPS chip by Higentec Co.,Ltd. (Changsha, China). Of 5420 possible SNP markers, 1859 were polymorphic between Ludao and GBXZ, with an average of 154.9 markers harbored on each chromosome, ranging from 106 (Chr. 12) to 221 (Chr. 1) (**Table 1**). Based on the recombination breakpoints caused by the progeny, a high-density genetic map contains 770 bin markers was finally obtained, which contained an average of 64.2 bin markers per chromosome, ranging from 34 (Chr. 12) to 92 (Chr. 1). The total length of the constructed linkage map was 4,680.8 cM, with an average bin marker interval of 6.1 cM. (**Table 1**).

**Table 1:** Distribution of polymorphic molecular markers throughout the 12 chromosomes.

Chromosome	Number of SNP markers	Number of Bin markers	Genetic distance (cM)
Chr.1	221	92	693.6
Chr.2	157	73	534.9
Chr.3	177	87	410.2
Chr.4	115	40	273.1
Chr.5	134	60	385.5
Chr.6	131	49	374.9
Chr.7	197	76	340.1
Chr.8	144	63	357.2
Chr.9	136	51	366.2
Chr.10	162	68	324.7
Chr.11	179	77	389.0
Chr.12	106	34	231.4
Total	1859	770	4680.8

### 3.3 Detection of QTLs for grain volume in rice

**Figure 2:**
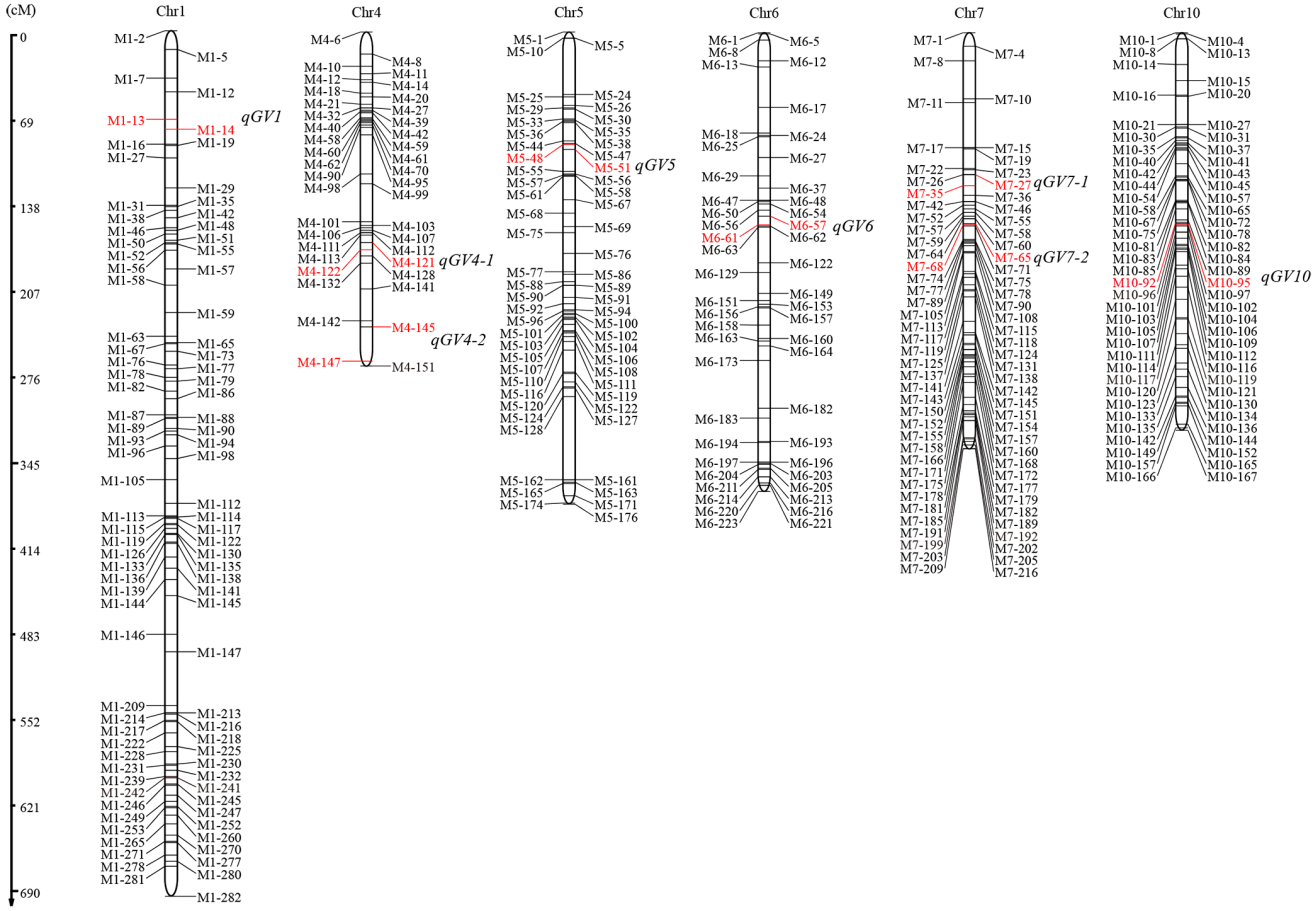
Distribution of QTLs for grain volume on rice chromosomes. Genetic map distances are shown on the left side of the linkage groups in cM and marker names are shown on both side of each chromosome, markers in red font indicate the location of corresponding QTL.

Using the ICIM method, we totally identified eight QTLs distributed on chromosomes 1, 4, 5, 6, 7, and 10, with LOD scores ranging from 3.33 to 13.25 (**Figure 2**). These QTLs individually explained 4.22-19.75% of the phenotypic variance in this population. Among these QTLs, the additive effect of *qGV1*, *qGV4-1*, *qGV5*, *qGV7-2* and *qGV10*, were positive, suggesting that the alleles from the Ludao parent increased grain volume. Conversely, the other three QTLs, *qGV4-2*, *qGV6* and *qGV7-1*, exhibited a negative effect, with the GBXZ alleles increasing grain volume (**Table 2**). These results indicated that grain volume was a complex trait controlled by numerous loci, and both parents contributed favorable alleles.

**Table 2:** QTLs for grain volume detected in LG population.

Trait locus	Chr	Marker interval	Physical position (bp)	LOD score	PVE (%)	Additive effect	Rnown loci
*qGV1*	1	M1-13~M1-14	3702591~4080706	3.8746	4.8404	0.6226	*qGL1* Liu et al. (2017)
*qGV4-1*	4	M4-121~M4-122	21032274~21538124	6.2766	7.7676	0.309	*qTGW4* Liu et al. (2017)
*qGV4-2*	4	M4-145~M4-147	31579302~34369506	3.6313	4.3782	-0.61	
*qGV5*	5	M5-48~M5-51	5622115~5864159	6.7859	8.0651	0.7791	*GW5/qSW5* Deng et al. (2017)
*qGV6*	6	M6-57~M6-61	10080211~10433606	3.3344	4.2237	-0.6517	*Pigm* Jiang et al. (2022)
*qGV7-1*	7	M7-27~M7-35	6690589~6813628	7.9765	10.3881	-0.9559	
*qGV7-2*	7	M7-65~M7-68	8880501~8910708	13.2496	19.7498	1.3563	
*qGV10*	10	M10-92~M10-95	13528116~13697797	11.5311	16.6025	1.3366	*qGW10* Wuriyanghan et al. (2009)

### 3.4 Epistatic interactions

**Figure 3:**
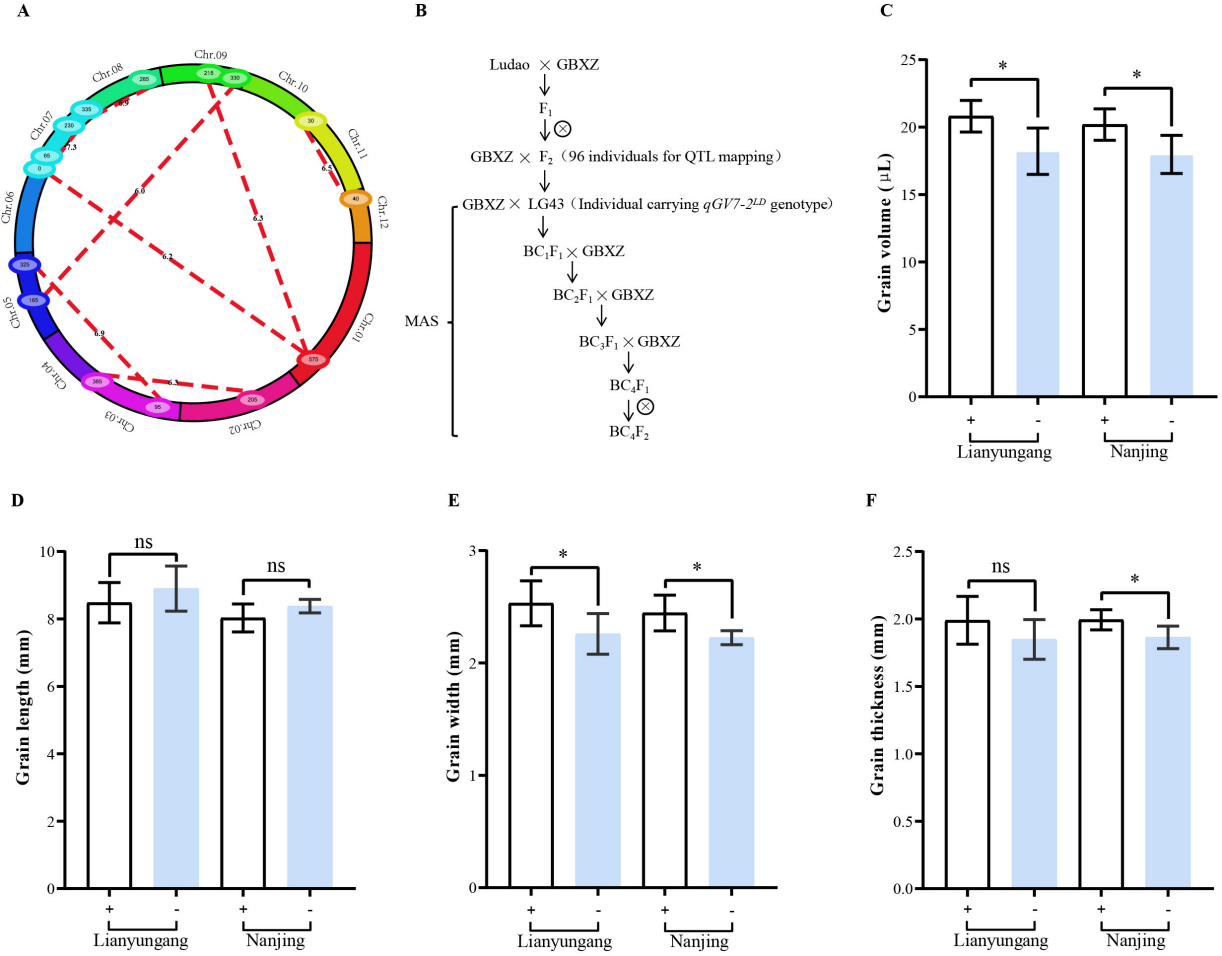
Epistasis analysis of QTLs for grain volume and validation of a major QTL (*qGV7-2*). **(A)** Cyclic graph for epistatic analysis. Red lines represents the epistatic effect among grain volume loci, the numbers on red lines represent the LOD score of epistatic QTLs. **(B)** Roadmap of constructing near-isogenic lines in Guangbaixiangzhan (GBXZ) genetic background. Grain volume **(C)**, grain length **(D)**, grain width **(E)**, and grain thickness **(F)** of near-isogenic lines at Lianyungang and Nanjing. + indicate lines carrying Ludao allele of *qGV7-2*, - indicate lines carrying GBXZ allele of *qGV7-2*, (n=6),* indicate significant differences at *P* < 0.05 by Student’s *t*-test, ns indicate not statistically significant.

Eight pairs of loci with significant epistatic effects were detected distributed on chromosomes 1, 2, 3, 5, 7, 8, 9, 11, and 12, with the LOD threshold set as 6.0 (**Figure 3A**). These QTLs individually explained 1.95 to 5.85% of the phenotypic variance in this population, and jointly they explained 24.63% of the total phenotypic variance (**Supplementary Table S1**). Among these, we identified significant epistatic interactions between the bin marker M1-225–M1-228 region on chromosome 1 and the bin marker M9-75–M9-78 region on chromosome 9 (LOD score of 6.33; 2.32% of PVE); between the bin marker M1-228–M1-230 region on chromosome 1 and M7-1–M7-4 region on chromosome 7 (6.17 and2.33%); between the bin marker M2-74–M2-85 region on chromosome 2 and M3-200–M3-244 region on chromosome 3 (6.34 and 3.37%); between the bin marker M3-69–M3-75 region on chromosome 3 and M5-128–M5-161 region on chromosome 5 (6.95 and 5.85%); between the bin marker M5-75–M5-76 region on chromosome 5 and M9-112–M9-118 region on chromosome 9 (6.01 and 1.95%); between the bin marker M7-11–M7-15 region on chromosome 7 and M7-138–M7-141 region on chromosome 7 (7.29 and 3.93%); between the bin marker M7-203–M7-205 region on chromosome 7 and M8-128–M8-132 region on chromosome 8 (6.89 and 2.56%); and between the bin marker M11-18–M11-26 region on chromosome 11 and M12-5–M12-17 region on chromosome 12 (6.49 and 2.31%). These results indicated there was a relatively large number of epistatic interacting loci regulating grain volume in the assessed mapping population, and these loci did not overlap additive QTL, indicating that epistatic effects play a pivotal role in regulating grain volume.

### 3.5 Validation of a major QTL *(qGV7-2*)

To further confirm the effect of *qGV7-2*, near-isogenic lines (NILs) of *qGV7-2* were constructed (**Figure 3B**). The genotype of NILs was assessed using a PCR-based genotyping protocol, indicating the proportion of GBXZ genotype was more than 95% (**Supplementary Table S2**). Phenotype comparison of NILs showed that the average grain volume of lines carrying Ludao allele was significantly greater than those carrying GBXZ allele in both locations, increasing by 14.29% and 12.22%, respectively (**Figure 3C**). No significant allelic effects was observed in grain length (**Figure 3D**), and lines carrying the Ludao allele demonstrated significantly greater grain width compared to those with the GBXZ allele at both experimental locations, increasing by 10.76% and 9.00%, respectively (**Figure 3E**). Interestingly, the thickness advantage of Ludao allele-containing lines reached statistical significance specifically under Nanjing conditions, exhibiting a 6.53% increase compared to GBXZ allele-containing lines (**Figure 3F**).

**Figure 4:**
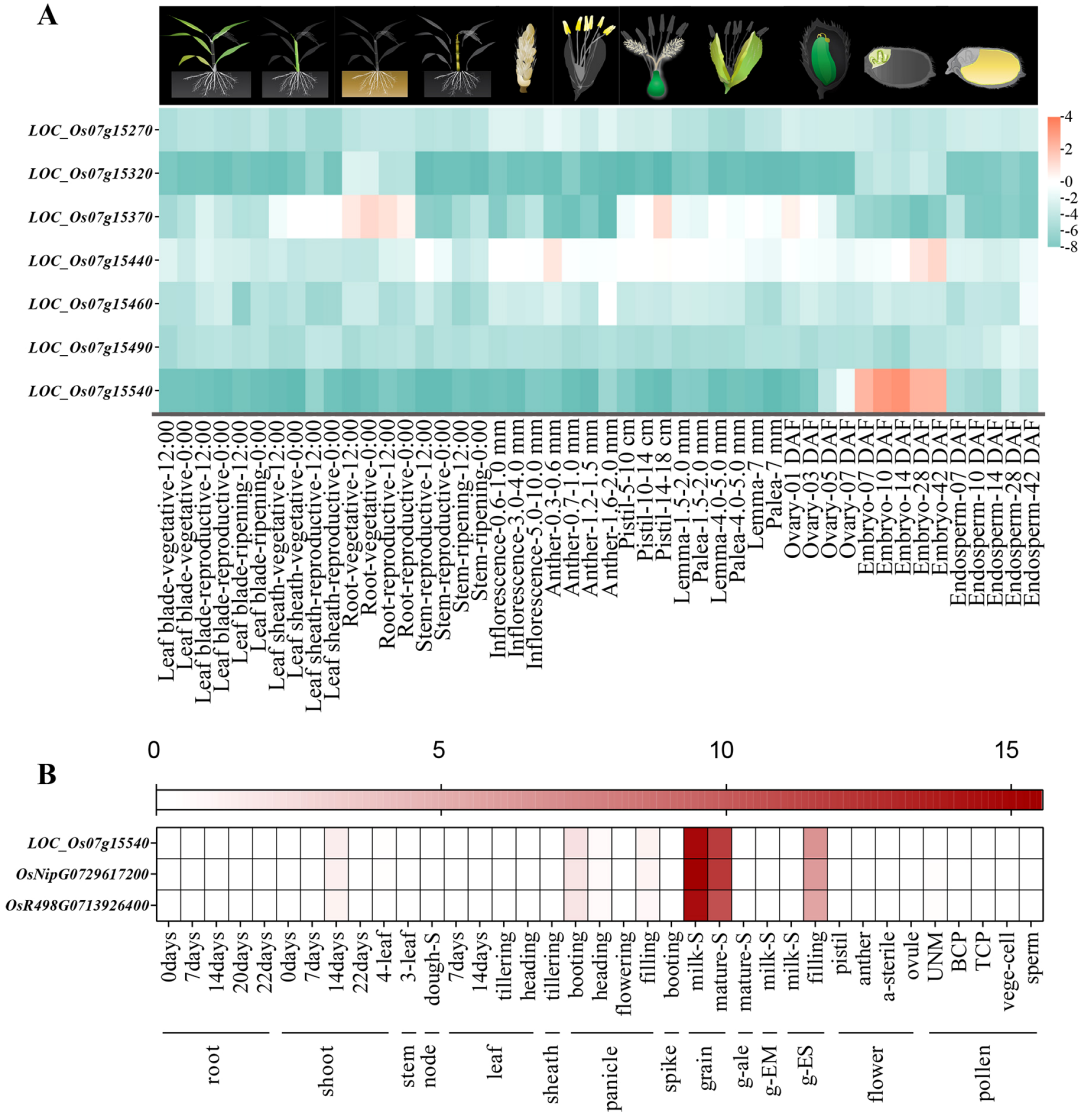
Expression profiling analysis of candidate genes in *qGV7-2* locus. **(A)** Expression profiling of seven candidate genes in *qGV7-2* locus (expression data from RiceXPro database), the pictures above the heatmap indicate organs/tissues sampled for gene expression analysis. **(B)** Expression analysis of *LOC-Os07g15540* in different developmental stages and organs of rice (expression data from MBKbase website).

By comparing the physical positions of QTLs detected in our study with those previously reported, we identified *qGV7-2* as a novel major QTL regulating grain volume. A total of 38 annotated genes were identified in the QTL region. Gene annotations of homologous *Arabidopsis thaliana* genes suggested seven of these genes may influence grain volume, and then, we used the RiceXPro database, to analyze the spatiotemporal expression patterns of these seven genes in different organs, and tissues (**Figure 4A**). One candidate gene, *LOC_Os07g15540*, was specifically and highly expressed in the ovary and embryo. Transcriptome analysis of *LOC_Os07g15540*, obtained from the MBKBase, revealed that this gene was highly expressed in organs associated with seed development (**Figure 4B**). Thus this gene may play an important role in seed development.

A sequence comparison between Ludao and GBXZ revealed that 15 SNPs in the coding region of *LOC_Os07g15540*, seven of which resulted in amino acid alterations. Specifically, the A/G base substitution at base 142 resulted in the substitution of aspartic acid with asparagine; A/G substitutions at 791 and 1541 resulted in the substitution of glycine with glutamic acid; A/G substitutions at 1195 and 1570 resulted in the substitution of valine with isoleucine; T/C substitution at 1319 resulted in the substitution of threonine with isoleucine; and G/C substitution at 2125 resulted in the substitution of arginine with glycine. All of these differential loci may affect protein function.

**Figure 5:**
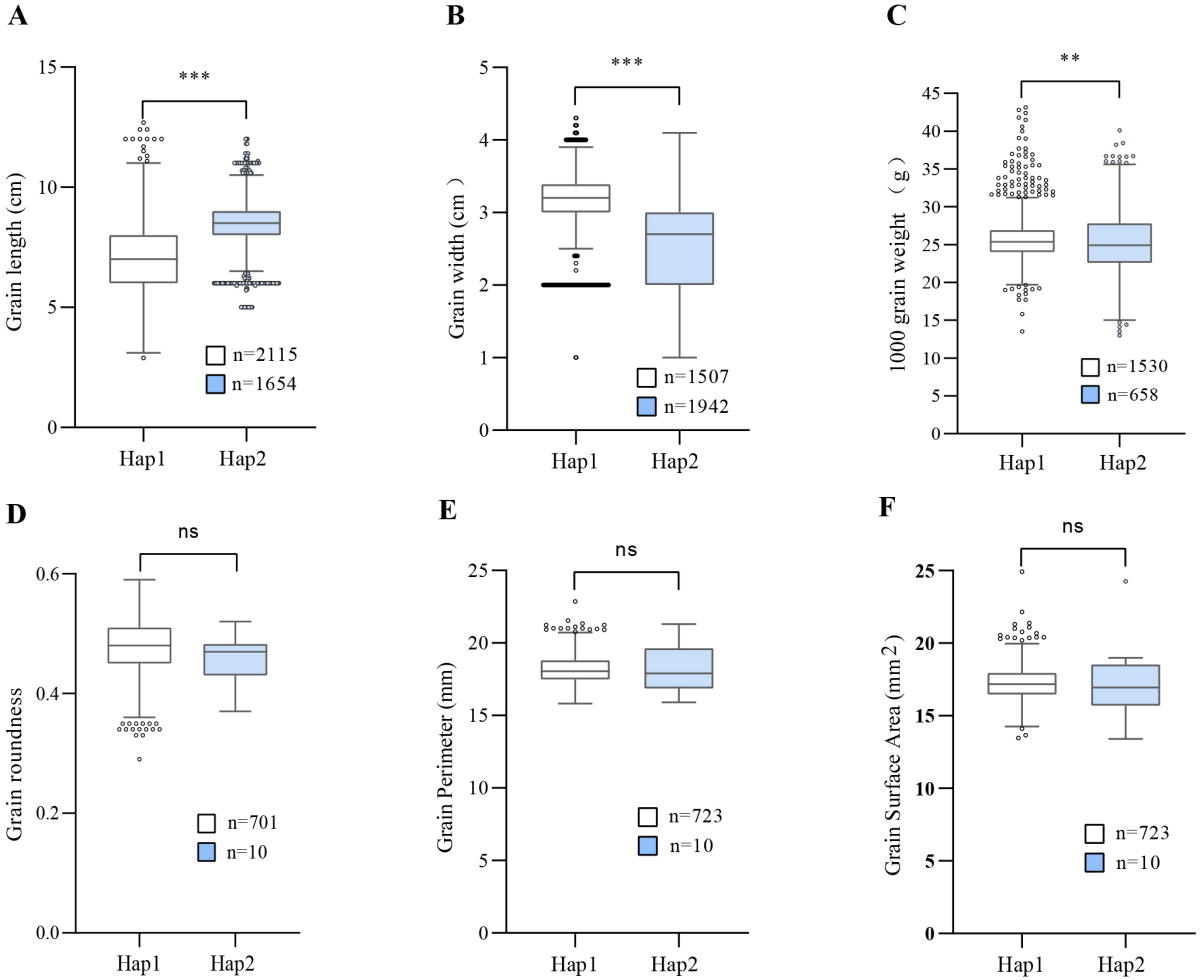
Comparison of different traits between two parental haplotypes of *LOC-Os07g15540*. Haplotype analysis of *LOC-Os07g15540* for grain length **(A)**, grain width **(B)**, thousand grain weight **(C)**, grain roundness **(D)**, grain perimeter **(E)**, grain surface area **(F)**. ** and *** indicates statistical significance at *P* < 0.01 and *P* < 0.001, respectively (Student’s *t*-test), ns indicate not statistically significant.

Based on the above results, the most likely candidate gene, *LOC_Os07g15540*, was submitted to the haplotype module of MBKBase for haplotype-phenotype analysis. Compared with the germplasms sharing the same haplotype with GBXZ (Hap2), that carrying Ludao haplotype (Hap1) exhibited a 15.64% reduction in grain length, and increases of 21.57% and 1.71% in grain width and TGW, respectively (**Figure 5**). On the basis of these findings, we thus hypothesized that the difference in grain volume between Ludao and GBXZ may be influenced by differences in grain length, grain width, and TGW. Taken together, *LOC_Os07g15540* is a possible candidate gene for *qGV7-2*.

## 4 Discussion

The mapping and cloning of genes associated with rice grain traits has significant implications for improving the quality and yield of rice. According to statistics obtained from the China Rice Data Center website (https://www.ricedata.cn/gene/), more than 300 grain traits-related QTLs/genes have been identified via genetic mapping, association analysis, and other methods. These grain trait-related genes/QTLs are distributed on almost all of the 12 chromosomes of rice, with relatively larger numbers found on chromosomes 2, 3 and 5 (Ponce et al., 2020). On the basis of a comparison of physical positions, we found that the QTLs identified here fit well with the QTLs and genes related to grain shape in previous studies. For example, the physical chromosomal positions of *qGV1* and *qGV4-1* identified in this study partially overlap with the locations of *qGL1* and *qTGW4* detected by Zhou et al. (2018). The *qGV5* may be associated with the *GW5/qSW5* gene, which controls grain width of rice (Shomura et al., 2008; Liu et al., 2017). Furthermore, the interval covered by *qGV6* detected in this study contains a broad-spectrum disease resistance gene, *PigmR*, the encoded protein of which can form homodimers and confers resistance against rice blast fungus M. *oryzae*. However *PigmR* transgenic plants displayed a yield loss due to the decrease in TGW and grain size relative to the control (Deng et al., 2017). Hence, we postulate that *PigmR* is a reasonable candidate gene for *qGV6*. Moreover, the interval covered by *qGV10* was found to coincide with that of the grain width QTL *qGW10* previously detected in this population (Sun et al., 2024). In addition, using the RiceVarMap v2.0 tool, we detected a locus significantly associated with TGW (Var ID: 0432427836) on rice chromosome 4 (**Supplementary Figure S1**), it shared a region with *qGV4-2*, a novel QTL detected in our study. In brief, the eight QTLs for grain volume detected in this study contained both previously mapped or cloned genes associated with grain traits, including *GL1*, *qTGW4* (Zhou et al., 2018), *GW5/qSW5* (Shomura et al., 2008; Liu et al., 2017), *PigmR* (Deng et al., 2017) and *qGW10* (Sun et al., 2024), as well as the newly discovered loci *qGV4-2*, *qGV7-1*, and *qGV7-2*. These findings would thus tend to indicate that our detection methods and results are authentic and reliable.

Grain traits are complex traits controlled by multiple genetic factors. On the basis of functional studies on previously cloned grain-related trait genes, it has been established that the regulatory networks involved in grain-related traits mainly include phytohormone signaling, ubiquitin proteasome, transcriptional regulators, and G protein signaling pathways (Jiang et al., 2022). In this study, a novel major QTL, *qGV7-2*, with a LOD score of 13.25 was detected on rice chromosome 7 (**Figure 2**; **Table 2**). A candidate gene, *LOC_Os07g15540*, was identified quickly and accurately by sequence comparison and expression analysis. Furthermore, haplotype-phenotype analysis revealed that the Ludao allele could increase grain width and TGW, whereas these two traits were significantly correlated with grain volume with correlation coefficients of 0.61 and 0.78, respectively (**Figure 1G**). Those results suggested that *LOC_Os07g15540* may play an important role in regulating grain volume. Hence, we accordingly conclude that this gene, which encodes an ethylene receptor OsETR4, is the most likely candidate gene. Although we obtained the candidate gene underlying *qGV7-2* using various analytical methods, this study still has some limitations. Further experiments, such as functional validation of candidate gene in transgenic lines, evaluation of QTL effects in different genetic backgrounds, need to be performed to verify our results more firmly.

In plants, ethylene receptors play crucial roles during ethylene signal transduction. Upon binding to ethylene, ethylene receptors undergo structural changes that not only influence the expression of associated genes but also modulate the interactions between different signaling pathways. These receptors consist of two subfamilies, namely, subfamily I (*OsERS1* and *OsERS2*) and subfamily II (*OsETR2*, *OsETR3*, and *OsETR4*). With the exception of *OsETR4*, the remaining four genes have been shown to play key regulatory roles in the ethylene signaling pathway (Wuriyanghan et al., 2009; Ma et al., 2013; Yang et al., 2015a). Ma et al. have demonstrated that rice grain size is regulated by ethylene, and found that *mhz7* mutant plants had smaller grain lengths and widths relative to the wild-type, whereas the overexpression of *MHZ7* contributed to increases in grain length (Ma et al., 2013). Similar findings have been reported for *Oseil1/mhz6* overexpression lines (Yang et al., 2015b), thus indicating that ethylene plays a positive role in regulating rice grain size.

Previous studies have shown that the etiolated seedlings of single loss-of-function ethylene receptor mutants are characterized by mild ethylene sensitivity, although showed essentially normal growth in the field (Wuriyanghan et al., 2009; Ma et al., 2013; Yin et al., 2015). In contrast, homozygous *Osers1+Osers2* double mutants have been observed to have severe growth issues, including produce seeds that can germinate, although the resulting seedlings tend to have poor survival (Zhou et al., 2019). These findings thus tend to indicate that members of ethylene receptor subfamily I may play essential regulatory roles during the growth of rice. In further studies, *OsETR2*, a member of ethylene receptor subfamily II, has been demonstrated to regulate the α-amylase gene *RAmy3D*, which causes an increase in starch accumulation, thereby affecting TGW and yield of rice (Wuriyanghan et al., 2009). Compared with other receptor genes, *OsETR4* is typically expressed at low levels, it shows high expression in spikelets at grain-filling process, thereby suggesting that *OsETR4* plays a key role during the grain-filling process (Sekhar et al., 2015). Collectively, these findings indicated that ethylene receptors play pivotal roles in regulating rice grain size. In addition, Jiang et al. have demonstrated that overexpression of *OsETR4* contributes to a significant enhancement of cold tolerance at the seedling stage (Jiang et al., 2020). Consistent with these observations, haplotype–phenotype analysis of *OsETR4* in this study revealed that the cold tolerance of germplasms, carrying the Ludao haplotype, was significantly higher than that of germplasms, carrying the GBXZ haplotype, at the bud burst and seedling stage (**Supplementary Figure S2**). These findings would thus tend to indicate that *OsETR4* regulates cold stress tolerance and yield in rice through ethylene signaling pathway.

## 5 Conclusion

In this study, a high-density genetic map was constructed by whole genome resequencing to identify QTLs related to grain volume of rice. Eight loci distributed on chromosomes 1, 4, 5, 6, 7 and 10 of rice were identified, with LOD scores ranging from 3.33 to 13.25, they jointly explained 76.02% of the total phenotypic variation. Among them, the novel locus, *qGV7-2*, with the highest PVE was further validated using NIL pairs. A promising candidate gene for *qGV7-2*, *LOC_Os07g15540*, was identified by expression, and haplotype-phenotype analysis. This study broadens our understanding of role of genetic factors in regulating grain volume, thus providing an important resource for yield improvement in rice.

## Data Availability

The datasets presented in this study can be found in online repositories. The names of the repository/repositories and accession number(s) can be found below: https://www.ebi.ac.uk/biostudies/studies/S-BSST1640, S-BSST1640.
