# Supplementary material for: Identification of QTLs and a candidate gene affecting rice grain volume via high-density genetic mapping
Source: Front Plant Sci. 2025 Mar 31;16:1579589. doi: 10.3389/fpls.2025.1579589 (PMC11994671; doi:10.3389/fpls.2025.1579589)
Supplement: Supplementary file 1 [file DataSheet1.pdf]

frontiers

Supplementary Figure

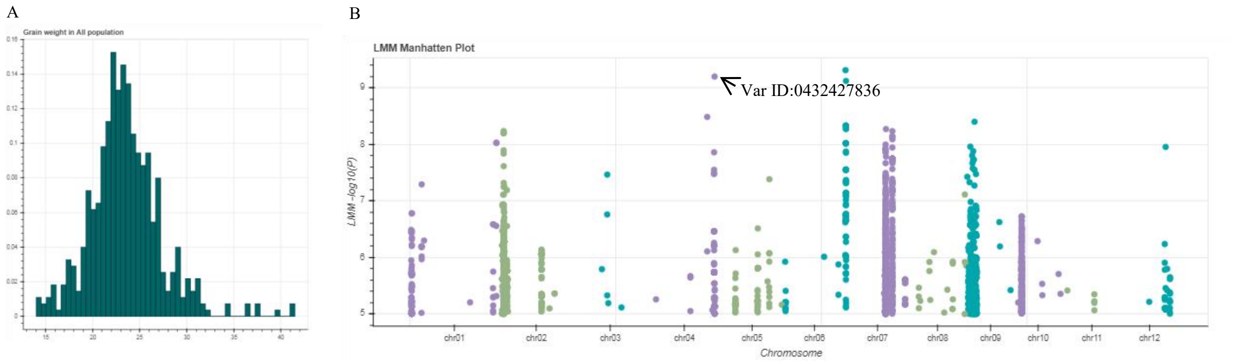

Supplementary Figure 1. GWAS for TGW using RiceVarMap data from a population of 529 rice accessions. (A) Frequency distribution of TGW among 529 rice accessions. (B) Manhattan plots of GWAS for TGW using 529 accessions. The black arrow indicates the identified locus.

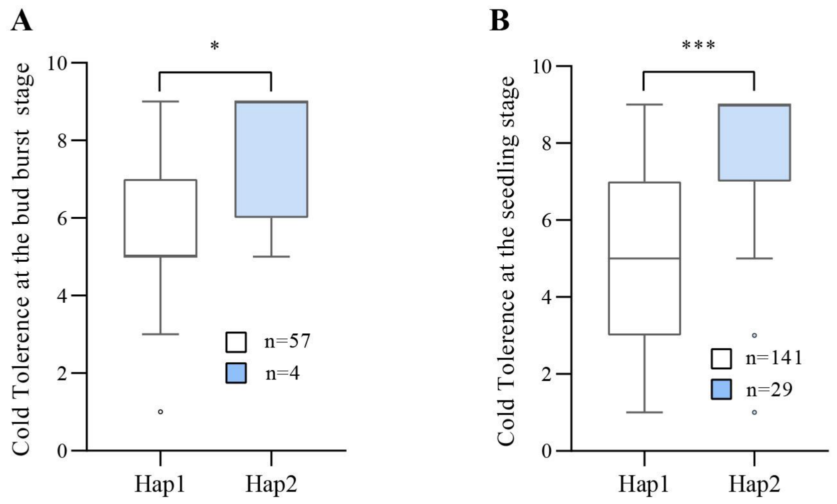

Supplementary Figure 2. Comparison of cold tolerance between two parental haplotypes of *LOC-Os07g15540*. Haplotype analysis of *LOC-Os07g15540* for cold tolerance at the bud burst (A) and seedling (B) stage.
