# Supplementary material for: Identification of QTLs and a candidate gene affecting rice grain volume via high-density genetic mapping
Source: Front Plant Sci. 2025 Mar 31;16:1579589. doi: 10.3389/fpls.2025.1579589 (PMC11994671; doi:10.3389/fpls.2025.1579589)
Supplement: Supplementary file 2 [file Table1.docx]

Supplementary Table

**Supplementary Table 1.** Epistatic QTLs for grain volume detected in the LG population

| **Chr1** | **Position1** | **Marker interval** | **Chr2** | **Position2** | **Marker interval** | **LOD** | **PVE(%)** |
| --- | --- | --- | --- | --- | --- | --- | --- |
| 2 | 205 | M2-74~M2-85 | 3 | 385 | M3-200~M3-244 | 6.342 | 3.3677 |
| 3 | 95 | M3-69~M3-75 | 5 | 325 | M5-128~M5-161 | 6.9464 | 5.853 |
| 1 | 580 | M1-228~M1-230 | 7 | 0 | M7-1~M7-4 | 6.1663 | 2.3276 |
| 7 | 65 | M7-11~M7-15 | 7 | 230 | M7-138~M7-141 | 7.2916 | 3.9335 |
| 7 | 335 | M7-203~M7-205 | 8 | 285 | M8-128~M8-132 | 6.887 | 2.5649 |
| 1 | 575 | M1-225~M1-228 | 9 | 215 | M9-75~M9-78 | 6.333 | 2.3157 |
| 5 | 165 | M5-75~M5-76 | 9 | 330 | M9-112~M9-118 | 6.0052 | 1.9523 |
| 11 | 30 | M11-18~M11-26 | 12 | 40 | M12-5~M12-17 | 6.4912 | 2.312 |

LOD score: (logarithm [base 10] of odds); PVE: phenotypic variation explained.

**Supplementary Table 2.** The genotype of NILs

| Name of different lines | GBXZ | NILLudao-1 | NILGBXZ-1 | NILLudao-2 | NILGBXZ-2 | NILLudao-3 | NILGBXZ-3 | NILLudao-4 | NILGBXZ-4 | NILLudao-5 | NILGBXZ-5 | NILLudao-6 | NILGBXZ-6 |
| --- | --- | --- | --- | --- | --- | --- | --- | --- | --- | --- | --- | --- | --- |
| Proportion of GBXZ genotype | 100% | 97.36% | 98.12% | 96.45% | 97.69% | 98.49% | 99.35% | 95.05% | 96.45% | 98.76% | 99.57% | 96.50% | 98.22% |
| Genotype | TT | TT | TT | TT | TT | TT | TT | TT | TT | TT | TT | TT | TT |
|  | GG | GG | GG | GG | GG | GG | GG | GG | GG | GG | GG | GG | GG |
|  | AA | AA | AA | AA | AA | AA | AA | AA | AA | AA | AA | AA | AA |
|  | TT | TT | TT | TT | TT | TT | TT | TT | TT | TT | TT | TT | TT |
|  | GG | GG | GG | GG | GG | GG | GG | GG | GG | GG | GG | GG | GG |
|  | GG | GG | GG | GG | GG | GG | GG | GG | GG | GG | GG | GG | GG |
|  | CC | CC | CC | CC | CC | CC | CC | CC | CC | CC | CC | CC | CC |
|  | GG | GG | GG | GG | GG | GG | GG | GG | GG | GG | GG | GG | GG |
|  | CC | CC | CC | CC | CC | CC | CC | CC | CC | CC | CC | CC | CC |
|  | TT | TT | TT | TT | TT | TT | TT | TT | TT | TT | TT | TT | TT |
|  | TT | TT | TT | TT | TT | TT | TT | TT | TT | TT | TT | TT | TT |
|  | CC | CC | CC | CC | CC | CC | CC | CC | CC | CC | CC | CC | CC |
|  | TT | TT | TT | TT | TT | TT | TT | TT | TT | TT | TT | TT | TT |
|  | AA | AA | AA | AA | AA | AA | AA | AA | AA | AA | AA | AA | AA |
|  | AA | AA | AA | AA | AA | AA | AA | AA | AA | AA | AA | AA | AA |
|  | GG | GG | GG | GG | GG | GG | GG | GG | GG | GG | GG | GG | GG |
|  | TT | TT | TT | TT | TT | TT | TT | TT | TT | TT | TT | TT | TT |
|  | AA | AA | AA | AA | AA | AA | AA | AA | AA | AA | AA | AA | AA |
|  | GG | GG | GG | GG | GG | GG | GG | GG | GG | GG | GG | GG | GG |
|  | GG | GG | GG | GG | GG | GG | GG | GG | GG | GG | GG | GG | GG |
|  | AA | AA | AA | AA | AA | AA | AA | AA | AA | AA | AA | AA | AA |
|  | GG | GG | GG | GG | GG | GG | GG | GG | GG | GG | GG | GG | GG |
|  | GG | GG | GG | GG | GG | GG | GG | GG | GG | GG | GG | GG | GG |
|  | AA | AA | AA | AA | AA | AA | AA | AA | AA | AA | AA | AA | AA |
|  | AA | AA | AA | AA | AA | AA | AA | AA | AA | AA | AA | AA | AA |
|  | GG | GG | GG | GG | GG | GG | GG | GG | GG | GG | GG | GG | GG |
|  | TT | TT | TT | TT | TT | TT | TT | TT | TT | TT | TT | TT | TT |
|  | AA | AA | AA | AA | AA | AA | AA | AA | AA | AA | AA | AA | AA |
|  | TT | TT | TT | TT | TT | TT | TT | TT | TT | TT | TT | TT | TT |
|  | GG | GG | GG | GG | GG | GG | GG | GG | GG | GG | GG | GG | GG |
|  | CC | CC | CC | CC | CC | CC | CC | CC | CC | CC | CC | CC | CC |
|  | AA | AA | AA | AA | AA | AA | AA | AA | AA | AA | AA | AA | AA |
|  | CC | CC | CC | CC | CC | CC | CC | CC | CC | CC | CC | CC | CC |
|  | AA | AA | AA | AA | AA | AA | AA | AA | AA | AA | AA | AA | AA |
|  | AA | AA | AA | AA | AA | AA | AA | AA | AA | AA | AA | AA | AA |
|  | GG | GG | GG | GG | GG | GG | GG | GG | GG | GG | GG | GG | GG |
|  | TT | TT | TT | TT | TT | TT | TT | TT | TT | TT | TT | TT | TT |
|  | GG | GG | GG | GG | GG | GG | GG | GG | GG | GG | GG | GG | GG |
|  | GG | GG | GG | GG | GG | GG | GG | GG | GG | GG | GG | GG | GG |
|  | GG | GG | GG | GG | GG | GG | GG | GG | GG | GG | GG | GG | GG |
|  | TT | TT | TT | TT | TT | TT | TT | TT | TT | TT | TT | TT | TT |
|  | CC | CC | CC | CC | CC | CC | CC | CC | CC | CC | CC | CC | CC |
|  | GG | GG | GG | GG | GG | GG | GG | GG | GG | GG | GG | GG | GG |
|  | CC | CC | CC | CC | CC | CC | CC | CC | CC | CC | CC | CC | CC |
|  | GG | GG | GG | GG | GG | GG | GG | GG | GG | GG | GG | GG | GG |
|  | AA | AA | AA | AA | AA | AA | AA | AA | AA | AA | AA | AA | AA |
|  | CC | CC | CC | CC | CC | CC | CC | CC | CC | CC | CC | CC | CC |
|  | GG | GG | GG | GG | GG | GG | GG | GG | GG | GG | GG | GG | GG |
|  | AA | AA | AA | AA | AA | AA | AA | AA | AA | AA | AA | AA | AA |
|  | CC | CC | CC | CC | CC | CC | CC | CC | CC | CC | CC | CC | CC |
|  | GG | GG | GG | GG | GG | GG | GG | GG | GG | GG | GG | GG | GG |
|  | AA | AA | AA | AA | AA | AA | AA | AA | AA | AA | AA | AA | AA |
|  | AA | AA | AA | AA | AA | AA | AA | AA | AA | AA | AA | AA | AA |
|  | TT | TT | TT | TT | TT | TT | TT | TT | TT | TT | TT | TT | TT |
|  | CC | CC | CC | CC | CC | CC | CC | CC | CC | CC | CC | CC | CC |
|  | CC | CC | CC | CC | CC | CC | CC | CC | CC | CC | CC | CC | CC |
|  | TT | TT | TT | TT | TT | TT | TT | TT | TT | TT | TT | TT | TT |
|  | AA | AA | AA | AA | AA | AA | AA | AA | AA | AA | AA | AA | AA |
|  | CC | CC | CC | CC | CC | CC | CC | CC | CC | CC | CC | CC | CC |
|  | TT | TT | TT | TT | TT | TT | TT | TT | TT | TT | TT | TT | TT |
|  | TT | TT | TT | TT | TT | TT | TT | TT | TT | TT | TT | TT | TT |
|  | CC | CC | CC | CC | CC | CC | CC | CC | CC | CC | CC | CC | CC |
|  | CC | CC | CC | CC | CC | CC | CC | CC | CC | CC | CC | CC | CC |
|  | TT | TT | TT | TT | TT | TT | TT | TT | TT | TT | TT | TT | TT |
|  | AA | AA | AA | AA | AA | AA | AA | AA | AA | AA | AA | AA | AA |
|  | AA | AA | AA | AA | AA | AA | AA | AA | AA | AA | AA | AA | AA |
|  | GG | GG | GG | GG | GG | GG | GG | GG | GG | GG | GG | GG | GG |
|  | AA | AA | AA | AA | AA | AA | AA | AA | AA | AA | AA | AA | AA |
|  | AA | AA | AA | AA | AA | AA | AA | AA | AA | AA | AA | AA | AA |
|  | TT | TT | TT | TT | TT | TT | TT | TT | TT | TT | TT | TT | TT |
|  | CC | CC | CC | CC | CC | CC | CC | CC | CC | CC | CC | CC | CC |
|  | TT | TT | TT | TT | TT | TT | TT | TT | TT | TT | TT | TT | TT |
|  | AA | AA | AA | AA | AA | AA | AA | AA | AA | AA | AA | AA | AA |
|  | GG | GG | GG | GG | GG | GG | GG | GG | GG | GG | GG | GG | GG |
|  | GG | GG | GG | GG | GG | GG | GG | GG | GG | GG | GG | GG | GG |
|  | CC | CC | CC | CC | CC | CC | CC | CC | CC | CC | CC | CC | CC |
|  | CC | CC | CC | CC | CC | CC | CC | CC | CC | CC | CC | CC | CC |
|  | CC | CC | CC | CC | CC | CC | CC | CC | CC | CC | CC | CC | CC |
|  | TT | TT | TT | TT | TT | TT | TT | TT | TT | TT | TT | TT | TT |
|  | TT | TT | TT | TT | TT | TT | TT | TT | TT | TT | TT | TT | TT |
|  | AA | AA | AA | AA | AA | AA | AA | AA | AA | AA | AA | AA | AA |
|  | AA | AA | AA | AA | AA | AA | AA | AA | AA | AA | AA | AA | AA |
|  | CC | CC | CC | CC | CC | CC | CC | CC | CC | CC | CC | CC | CC |
|  | CC | CC | CC | CC | CC | CC | CC | CC | CC | CC | CC | CC | CC |
|  | TT | TT | TT | TT | TT | TT | TT | TT | TT | TT | TT | TT | TT |
|  | GG | GG | GG | GG | GG | GG | GG | GG | GG | GG | GG | GG | GG |
|  | AA | AA | AA | AA | AA | AA | AA | AA | AA | AA | AA | AA | AA |
|  | TT | TT | TT | TT | TT | TT | TT | TT | TT | TT | TT | TT | TT |
|  | TT | TT | TT | TT | TT | TT | TT | TT | TT | TT | TT | TT | TT |
|  | AA | AA | AA | AA | AA | AA | AA | AA | AA | AA | AA | AA | AA |
|  | AA | AA | AA | AA | AA | AA | AA | AA | AA | AA | AA | AA | AA |
|  | TT | TT | TT | TT | TT | TT | TT | TT | TT | TT | TT | TT | TT |
|  | TT | TT | TT | TT | TT | TT | TT | TT | TT | TT | TT | TT | TT |
|  | TT | TT | TT | TT | TT | TT | TT | TT | TT | TT | TT | TT | TT |
|  | GG | GG | GG | GG | GG | GG | GG | GG | GG | GG | GG | GG | GG |
|  | CC | CC | CC | CC | CC | CC | CC | CC | CC | CC | CC | CC | CC |
|  | GG | GG | GG | GG | GG | GG | GG | GG | GG | GG | GG | GG | GG |
|  | GG | GG | GG | GG | GG | GG | GG | GG | GG | GG | GG | GG | GG |
|  | CC | CC | CC | CC | CC | CC | CC | CC | CC | CC | CC | CC | CC |
|  | TT | TT | TT | TT | TT | TT | TT | TT | TT | TT | TT | TT | TT |
|  | GG | GG | GG | GG | GG | GG | GG | GG | GG | GG | GG | GG | GG |
|  | CC | CC | CC | CC | CC | CC | CC | CC | CC | CC | CC | CC | CC |
|  | TT | TT | TT | TT | TT | TT | TT | TT | TT | TT | TT | TT | TT |
|  | CC | CC | CC | CC | CC | CC | CC | CC | CC | CC | CC | CC | CC |
|  | TT | TT | TT | TT | TT | TT | TT | TT | TT | TT | TT | TT | TT |
|  | TT | TT | TT | TT | TT | TT | TT | TT | TT | TT | TT | TT | TT |
|  | AA | AA | AA | AA | AA | AA | AA | AA | AA | AA | AA | AA | AA |
|  | TT | TT | TT | TT | TT | TT | TT | TT | TT | TT | TT | TT | TT |
|  | CC | CC | CC | CC | CC | CC | CC | CC | CC | CC | CC | CC | CC |
|  | TT | TT | TT | TT | TT | TT | TT | TT | TT | TT | TT | TT | TT |
|  | GG | GG | GG | GG | GG | GG | GG | GG | GG | GG | GG | GG | GG |
|  | TT | TT | TT | TT | TT | TT | TT | TT | TT | TT | TT | TT | TT |
|  | TT | TT | TT | TT | TT | TT | TT | TT | TT | TT | TT | TT | TT |
|  | TT | TT | TT | TT | TT | TT | TT | TT | TT | TT | TT | TT | TT |
|  | TT | TT | TT | TT | TT | TT | TT | TT | TT | TT | TT | TT | TT |
|  | CC | CC | CC | CC | CC | CC | CC | CC | CC | CC | CC | CC | CC |
|  | AA | AA | AA | AA | AA | AA | AA | AA | AA | AA | AA | AA | AA |
|  | AA | AA | AA | AA | AA | AA | AA | AA | AA | AA | AA | AA | AA |
|  | CC | CC | CC | CC | CC | CC | CC | CC | CC | CC | CC | CC | CC |
|  | GG | GG | GG | GG | GG | GG | GG | GG | GG | GG | GG | GG | GG |
|  | AA | AA | AA | AA | AA | AA | AA | AA | AA | AA | AA | AA | AA |
|  | TT | TT | TT | TT | TT | TT | TT | TT | TT | TT | TT | TT | TT |
|  | GG | GG | GG | GG | GG | GG | GG | GG | GG | GG | GG | GG | GG |
|  | AA | AA | AA | AA | AA | AA | AA | AA | AA | AA | AA | AA | AA |
|  | TT | TT | TT | TT | TT | TT | TT | TT | TT | TT | TT | TT | TT |
|  | CC | CC | CC | CC | CC | CC | CC | CC | CC | CC | CC | CC | CC |
|  | AA | AA | AA | AA | AA | AA | AA | AA | AA | AA | AA | AA | AA |
|  | AA | AA | AA | AA | AA | AA | AA | AA | AA | AA | AA | AA | AA |
|  | TT | TT | TT | TT | TT | TT | TT | TT | TT | TT | TT | TT | TT |
|  | AA | AA | AA | AA | AA | AA | AA | AA | AA | AA | AA | AA | AA |
|  | GG | GG | GG | GG | GG | GG | GG | GG | GG | GG | GG | GG | GG |
|  | CC | CC | CC | CC | CC | CC | CC | CC | CC | CC | CC | CC | CC |
|  | TT | TT | TT | TT | TT | TT | TT | TT | TT | TT | TT | TT | TT |
|  | CC | CC | CC | CC | CC | CC | CC | CC | CC | CC | CC | CC | CC |
|  | TT | TT | TT | TT | TT | TT | TT | TT | TT | TT | TT | TT | TT |
|  | AA | AA | AA | AA | AA | AA | AA | AA | AA | AA | AA | AA | AA |
|  | GG | GG | GG | GG | GG | GG | GG | GG | GG | GG | GG | GG | GG |
|  | GG | GG | GG | GG | GG | GG | GG | GG | GG | GG | GG | GG | GG |
|  | AA | AA | AA | AA | AA | AA | AA | AA | AA | AA | AA | AA | AA |
|  | GG | GG | GG | GG | GG | GG | GG | GG | GG | GG | GG | GG | GG |
|  | TT | TT | TT | TT | TT | TT | TT | TT | TT | TT | TT | TT | TT |
|  | AA | AA | AA | AA | AA | AA | AA | AA | AA | AA | AA | AA | AA |
|  | AA | AA | AA | AA | AA | AA | AA | AA | AA | AA | AA | AA | AA |
|  | AA | AA | AA | AA | AA | AA | AA | AA | AA | AA | AA | AA | AA |
|  | GG | GG | GG | GG | GG | GG | GG | GG | GG | GG | GG | GG | GG |
|  | AA | AA | AA | AA | AA | AA | AA | AA | AA | AA | AA | AA | AA |
|  | GG | GG | GG | GG | GG | GG | GG | AA | AA | GG | GG | GG | GG |
|  | GG | GG | GG | GG | GG | GG | GG | TT | TT | GG | GG | GG | GG |
|  | CC | CC | CC | CC | CC | CC | CC | GG | GG | CC | CC | CC | CC |
|  | GG | GG | GG | GG | GG | GG | GG | AA | AA | GG | GG | GG | GG |
|  | TT | TT | TT | TT | TT | TT | TT | GG | GG | TT | TT | TT | TT |
|  | TT | TT | TT | TT | TT | TT | TT | CC | CC | TT | TT | TT | TT |
|  | AA | AA | AA | AA | AA | AA | AA | GG | GG | AA | AA | AA | AA |
|  | AA | AA | AA | AA | AA | AA | AA | GG | GG | AA | AA | AA | AA |
|  | AA | AA | AA | AA | AA | AA | AA | GG | GG | AA | AA | AA | AA |
|  | GG | GG | GG | GG | GG | GG | GG | AA | AA | GG | GG | GG | GG |
|  | CC | CC | CC | CC | CC | CC | CC | GG | GG | CC | CC | CC | CC |
|  | GG | GG | GG | GG | GG | GG | GG | AA | AA | GG | GG | GG | GG |
|  | CC | CC | CC | CC | CC | CC | CC | TT | TT | CC | CC | CC | CC |
|  | AA | AA | AA | AA | AA | AA | AA | AA | AA | AA | AA | AA | AA |
|  | CC | CC | CC | CC | CC | CC | CC | CC | CC | CC | CC | CC | CC |
|  | AA | AA | AA | AA | AA | AA | AA | AA | AA | AA | AA | AA | AA |
|  | AA | AA | AA | AA | AA | AA | AA | AA | AA | AA | AA | AA | AA |
|  | TT | TT | TT | TT | TT | TT | TT | TT | TT | TT | TT | TT | TT |
|  | AA | AA | AA | AA | AA | AA | AA | AA | AA | AA | AA | AA | AA |
|  | CC | CC | CC | CC | CC | CC | CC | CC | CC | CC | CC | CC | CC |
|  | TT | TT | TT | TT | TT | TT | TT | TT | TT | TT | TT | TT | TT |
|  | CC | CC | CC | CC | CC | CC | CC | CC | CC | CC | CC | CC | CC |
|  | GG | GG | GG | GG | GG | GG | GG | GG | GG | GG | GG | GG | GG |
|  | GG | GG | GG | GG | GG | GG | GG | GG | GG | GG | GG | GG | GG |
|  | AA | AA | AA | AA | AA | AA | AA | AA | AA | AA | AA | AA | AA |
|  | CC | CC | CC | CC | CC | CC | CC | CC | CC | CC | CC | CC | CC |
|  | TT | TT | TT | TT | TT | TT | TT | TT | TT | TT | TT | TT | TT |
|  | TT | TT | TT | TT | TT | TT | TT | TT | TT | TT | TT | TT | TT |
|  | TT | TT | TT | TT | TT | TT | TT | TT | TT | TT | TT | TT | TT |
|  | AA | AA | AA | AA | AA | AA | AA | AA | AA | AA | AA | AA | AA |
|  | AA | GG | GG | AA | AA | AA | AA | AA | AA | AA | AA | AA | AA |
|  | CC | TT | TT | CC | CC | CC | CC | CC | CC | CC | CC | CC | CC |
|  | GG | AA | AA | GG | GG | GG | GG | GG | GG | GG | GG | GG | GG |
|  | CC | CC | CC | CC | CC | CC | CC | CC | CC | CC | CC | CC | CC |
|  | AA | AA | AA | AA | AA | AA | AA | AA | AA | AA | AA | AA | AA |
|  | CC | CC | CC | CC | CC | CC | CC | CC | CC | CC | CC | CC | CC |
|  | AA | AA | AA | AA | AA | AA | AA | AA | AA | AA | AA | AA | AA |
|  | AA | AA | AA | AA | AA | AA | AA | AA | AA | AA | AA | AA | AA |
|  | GG | GG | GG | GG | GG | GG | GG | GG | GG | GG | GG | GG | GG |
|  | GG | GG | GG | GG | GG | GG | GG | GG | GG | GG | GG | GG | GG |
|  | TT | TT | TT | TT | TT | TT | TT | TT | TT | TT | TT | TT | TT |
|  | CC | CC | CC | CC | CC | CC | CC | CC | CC | CC | CC | CC | CC |
|  | AA | AA | AA | AA | AA | AA | AA | AA | AA | AA | AA | GG | GG |
|  | AA | AA | AA | AA | AA | AA | AA | AA | AA | AA | AA | TT | TT |
|  | CC | CC | CC | CC | CC | CC | CC | CC | CC | CC | CC | TT | TT |
|  | GG | GG | GG | GG | GG | GG | GG | GG | GG | GG | GG | AA | AA |
|  | AA | AA | AA | AA | AA | AA | AA | AA | AA | AA | AA | GG | GG |
|  | AA | AA | AA | AA | AA | AA | AA | AA | AA | AA | AA | GG | GG |
|  | GG | GG | GG | GG | GG | GG | GG | GG | GG | GG | GG | AA | AA |
|  | GG | GG | GG | GG | GG | GG | GG | GG | GG | GG | GG | AA | AA |
|  | GG | GG | GG | GG | GG | GG | GG | GG | GG | GG | GG | TT | TT |
|  | TT | TT | TT | TT | TT | TT | TT | TT | TT | TT | TT | CC | CC |
|  | AA | AA | AA | AA | AA | AA | AA | AA | AA | AA | AA | GG | GG |
|  | TT | TT | TT | TT | TT | TT | TT | TT | TT | TT | TT | CC | CC |
|  | CC | CC | CC | CC | CC | CC | CC | CC | CC | CC | CC | CC | CC |
|  | GG | GG | GG | GG | GG | GG | GG | GG | GG | GG | GG | GG | GG |
|  | AA | AA | AA | AA | AA | AA | AA | AA | AA | AA | AA | AA | AA |
|  | CC | CC | CC | CC | CC | CC | CC | CC | CC | CC | CC | CC | CC |
|  | GG | GG | GG | GG | GG | GG | GG | GG | GG | GG | GG | GG | GG |
|  | TT | TT | TT | TT | TT | TT | TT | TT | TT | TT | TT | TT | TT |
|  | GG | GG | GG | GG | GG | GG | GG | GG | GG | GG | GG | GG | GG |
|  | CC | CC | CC | CC | CC | CC | CC | CC | CC | CC | CC | CC | CC |
|  | CC | CC | CC | CC | CC | CC | CC | CC | CC | CC | CC | CC | CC |
|  | GG | GG | GG | GG | GG | GG | GG | GG | GG | GG | GG | GG | GG |
|  | CC | CC | CC | CC | CC | CC | CC | CC | CC | CC | CC | CC | CC |
|  | CC | CC | CC | CC | CC | CC | CC | CC | CC | CC | CC | CC | CC |
|  | GG | GG | GG | GG | GG | GG | GG | GG | GG | GG | GG | GG | GG |
|  | TT | TT | TT | TT | TT | TT | TT | TT | TT | TT | TT | TT | TT |
|  | AA | AA | AA | AA | AA | AA | AA | AA | AA | AA | AA | AA | AA |
|  | AA | AA | AA | AA | AA | AA | AA | AA | AA | AA | AA | AA | AA |
|  | CC | CC | CC | CC | CC | CC | CC | CC | CC | CC | CC | CC | CC |
|  | AA | AA | AA | AA | AA | AA | AA | AA | AA | AA | AA | AA | AA |
|  | CC | CC | CC | CC | CC | CC | CC | CC | CC | CC | CC | CC | CC |
|  | GG | GG | GG | GG | GG | GG | GG | GG | GG | GG | GG | GG | GG |
|  | GG | GG | GG | GG | GG | GG | GG | GG | GG | GG | GG | GG | GG |
|  | CC | CC | CC | CC | CC | CC | CC | CC | CC | CC | CC | CC | CC |
|  | CC | CC | CC | CC | CC | CC | CC | CC | CC | CC | CC | CC | CC |
|  | TT | TT | TT | TT | TT | TT | TT | TT | TT | TT | TT | TT | TT |
|  | AA | AA | AA | AA | AA | AA | AA | AA | AA | AA | AA | AA | AA |
|  | CC | CC | CC | CC | CC | CC | CC | CC | CC | CC | CC | CC | CC |
|  | TT | TT | TT | TT | TT | TT | TT | TT | TT | TT | TT | TT | TT |
|  | GG | GG | GG | GG | GG | GG | GG | GG | GG | GG | GG | GG | GG |
|  | CC | CC | CC | CC | CC | CC | CC | CC | CC | CC | CC | CC | CC |
|  | GG | GG | GG | GG | GG | GG | GG | GG | GG | GG | GG | GG | GG |
|  | AA | AA | AA | AA | AA | AA | AA | AA | AA | AA | AA | AA | AA |
|  | CC | CC | CC | CC | CC | CC | CC | CC | CC | CC | CC | CC | CC |
|  | CC | CC | CC | CC | CC | CC | CC | CC | CC | CC | CC | CC | CC |
|  | AA | AA | AA | AA | AA | AA | AA | AA | AA | AA | AA | AA | AA |
|  | AA | AA | AA | AA | AA | AA | AA | AA | AA | AA | AA | AA | AA |
|  | GG | GG | GG | GG | GG | GG | GG | GG | GG | GG | GG | GG | GG |
|  | TT | TT | TT | TT | TT | TT | TT | TT | TT | TT | TT | TT | TT |
|  | AA | AA | AA | AA | AA | AA | AA | AA | AA | AA | AA | AA | AA |
|  | TT | TT | TT | TT | TT | TT | TT | TT | TT | TT | TT | TT | TT |
|  | GG | GG | GG | GG | GG | GG | GG | GG | GG | GG | GG | GG | GG |
|  | AA | GG | GG | AA | AA | AA | AA | AA | AA | AA | AA | AA | AA |
|  | TT | CC | CC | TT | TT | TT | TT | TT | TT | TT | TT | TT | TT |
|  | TT | CC | CC | TT | TT | TT | TT | TT | TT | TT | TT | TT | TT |
|  | GG | AA | AA | GG | GG | GG | GG | GG | GG | GG | GG | GG | GG |
|  | GG | GG | GG | GG | GG | GG | GG | GG | GG | GG | GG | GG | GG |
|  | AA | AA | AA | AA | AA | AA | AA | AA | AA | AA | AA | AA | AA |
|  | AA | AA | AA | AA | AA | AA | AA | AA | AA | AA | AA | AA | AA |
|  | AA | AA | AA | AA | AA | AA | AA | AA | AA | AA | AA | AA | AA |
|  | GG | GG | GG | GG | GG | GG | GG | GG | GG | GG | GG | GG | GG |
|  | GG | GG | GG | GG | GG | GG | GG | GG | GG | GG | GG | GG | GG |
|  | GG | GG | GG | GG | GG | GG | GG | GG | GG | GG | GG | GG | GG |
|  | AA | AA | AA | AA | AA | AA | AA | AA | AA | AA | AA | AA | AA |
|  | CC | CC | CC | CC | CC | CC | CC | CC | CC | CC | CC | CC | CC |
|  | TT | TT | TT | TT | TT | TT | TT | TT | TT | TT | TT | TT | TT |
|  | TT | TT | TT | TT | TT | TT | TT | TT | TT | TT | TT | TT | TT |
|  | GG | GG | GG | GG | GG | GG | GG | GG | GG | GG | GG | GG | GG |
|  | TT | TT | TT | TT | TT | TT | TT | TT | TT | TT | TT | TT | TT |
|  | GG | GG | GG | GG | GG | GG | GG | GG | GG | GG | GG | GG | GG |
|  | GG | GG | GG | GG | GG | GG | GG | GG | GG | GG | GG | GG | GG |
|  | GG | GG | GG | GG | GG | GG | GG | GG | GG | GG | GG | GG | GG |
|  | GG | GG | GG | GG | GG | GG | GG | GG | GG | GG | GG | GG | GG |
|  | CC | CC | CC | CC | CC | CC | CC | CC | CC | CC | CC | CC | CC |
|  | CC | CC | CC | CC | CC | CC | CC | CC | CC | CC | CC | CC | CC |
|  | AA | AA | AA | AA | AA | AA | AA | AA | AA | AA | AA | AA | AA |
|  | AA | AA | AA | AA | AA | AA | AA | AA | AA | AA | AA | AA | AA |
|  | TT | TT | TT | TT | TT | TT | TT | TT | TT | TT | TT | TT | TT |
|  | AA | AA | AA | AA | AA | AA | AA | AA | AA | AA | AA | AA | AA |
|  | AA | AA | AA | AA | AA | AA | AA | AA | AA | AA | AA | AA | AA |
|  | GG | GG | GG | GG | GG | GG | GG | GG | GG | GG | GG | GG | GG |
|  | TT | TT | TT | TT | TT | TT | TT | TT | TT | TT | TT | TT | TT |
|  | CC | CC | CC | CC | CC | CC | CC | CC | CC | CC | CC | CC | CC |
|  | GG | GG | GG | GG | GG | GG | GG | GG | GG | GG | GG | GG | GG |
|  | AA | AA | AA | AA | AA | AA | AA | AA | AA | AA | AA | AA | AA |
|  | AA | AA | AA | AA | AA | AA | AA | AA | AA | AA | AA | AA | AA |
|  | CC | CC | CC | CC | CC | CC | CC | CC | CC | CC | CC | CC | CC |
|  | TT | TT | TT | TT | TT | TT | TT | TT | TT | TT | TT | TT | TT |
|  | CC | CC | CC | CC | CC | CC | CC | CC | CC | CC | CC | CC | CC |
|  | CC | CC | CC | CC | CC | CC | CC | CC | CC | CC | CC | CC | CC |
|  | GG | GG | GG | GG | GG | GG | GG | GG | GG | GG | GG | GG | GG |
|  | TT | TT | TT | TT | TT | TT | TT | TT | TT | TT | TT | TT | TT |
|  | AA | AA | AA | AA | AA | AA | AA | AA | AA | AA | AA | AA | AA |
|  | GG | GG | GG | GG | GG | GG | GG | GG | GG | GG | GG | GG | GG |
|  | CC | CC | CC | CC | CC | CC | CC | CC | CC | CC | CC | CC | CC |
|  | CC | CC | CC | CC | CC | CC | CC | CC | CC | CC | CC | CC | CC |
|  | AA | AA | AA | AA | AA | AA | AA | AA | AA | AA | AA | AA | AA |
|  | TT | TT | TT | TT | TT | TT | TT | TT | TT | TT | TT | TT | TT |
|  | AA | AA | AA | AA | AA | AA | AA | AA | AA | AA | AA | AA | AA |
|  | CC | CC | CC | CC | CC | CC | CC | CC | CC | CC | CC | CC | CC |
|  | AA | AA | AA | AA | AA | AA | AA | AA | AA | AA | AA | AA | AA |
|  | CC | CC | CC | CC | CC | CC | CC | CC | CC | CC | CC | CC | CC |
|  | TT | TT | TT | TT | TT | TT | TT | TT | TT | TT | TT | TT | TT |
|  | CC | CC | CC | CC | CC | CC | CC | CC | CC | CC | CC | CC | CC |
|  | GG | GG | GG | GG | GG | GG | GG | GG | GG | GG | GG | GG | GG |
|  | TT | TT | TT | TT | TT | TT | TT | TT | TT | TT | TT | TT | TT |
|  | AA | AA | AA | AA | AA | AA | AA | AA | AA | AA | AA | AA | AA |
|  | GG | GG | GG | GG | GG | GG | GG | GG | GG | GG | GG | GG | GG |
|  | GG | GG | GG | GG | GG | GG | GG | GG | GG | GG | GG | GG | GG |
|  | TT | TT | TT | TT | TT | TT | TT | TT | TT | TT | TT | TT | TT |
|  | TT | TT | TT | TT | TT | TT | TT | TT | TT | TT | TT | TT | TT |
|  | GG | GG | GG | GG | GG | GG | GG | GG | GG | GG | GG | GG | GG |
|  | AA | AA | AA | AA | AA | AA | AA | AA | AA | AA | AA | AA | AA |
|  | CC | CC | CC | CC | CC | CC | CC | CC | CC | CC | CC | CC | CC |
|  | CC | CC | CC | CC | CC | CC | CC | CC | CC | CC | CC | CC | CC |
|  | TT | TT | TT | TT | TT | TT | TT | TT | TT | TT | TT | TT | TT |
|  | AA | AA | AA | AA | AA | AA | AA | AA | AA | AA | AA | AA | AA |
|  | GG | GG | GG | GG | GG | GG | GG | GG | GG | GG | GG | GG | GG |
|  | TT | TT | TT | TT | TT | TT | TT | TT | TT | TT | TT | TT | TT |
|  | CC | CC | CC | CC | CC | CC | CC | CC | CC | CC | CC | CC | CC |
|  | GG | GG | GG | GG | GG | GG | GG | GG | GG | GG | GG | GG | GG |
|  | TT | TT | TT | TT | TT | TT | TT | TT | TT | TT | TT | TT | TT |
|  | GG | GG | GG | GG | GG | GG | GG | GG | GG | GG | GG | GG | GG |
|  | CC | CC | CC | CC | CC | CC | CC | CC | CC | CC | CC | CC | CC |
|  | AA | AA | AA | AA | AA | AA | AA | AA | AA | AA | AA | AA | AA |
|  | AA | AA | AA | AA | AA | AA | AA | AA | AA | AA | AA | AA | AA |
|  | TT | TT | TT | TT | TT | TT | TT | TT | TT | TT | TT | TT | TT |
|  | TT | TT | TT | TT | TT | TT | TT | TT | TT | TT | TT | TT | TT |
|  | TT | TT | TT | TT | TT | TT | TT | TT | TT | TT | TT | TT | TT |
|  | TT | TT | TT | TT | TT | TT | TT | TT | TT | TT | TT | TT | TT |
|  | GG | GG | GG | GG | GG | GG | GG | GG | GG | GG | GG | GG | GG |
|  | GG | GG | GG | GG | GG | GG | GG | GG | GG | AA | AA | GG | GG |
|  | GG | AA | AA | GG | GG | GG | GG | GG | GG | AA | AA | GG | GG |
|  | AA | TT | TT | AA | AA | AA | AA | AA | AA | TT | TT | AA | AA |
|  | AA | CC | CC | AA | AA | AA | AA | AA | AA | CC | CC | AA | AA |
|  | TT | CC | CC | TT | TT | TT | TT | TT | TT | TT | TT | TT | TT |
|  | GG | AA | AA | GG | GG | GG | GG | GG | GG | GG | GG | GG | GG |
|  | CC | TT | TT | CC | CC | CC | CC | CC | CC | CC | CC | CC | CC |
|  | TT | TT | TT | TT | TT | TT | TT | TT | TT | TT | TT | TT | TT |
|  | AA | AA | AA | AA | AA | AA | AA | AA | AA | AA | AA | AA | AA |
|  | AA | AA | AA | AA | AA | AA | AA | AA | AA | AA | AA | AA | AA |
|  | GG | GG | GG | GG | GG | GG | GG | GG | GG | GG | GG | GG | GG |
|  | TT | TT | TT | TT | TT | TT | TT | TT | TT | TT | TT | TT | TT |
|  | CC | CC | CC | CC | CC | CC | CC | CC | CC | CC | CC | CC | CC |
|  | TT | TT | TT | TT | TT | TT | TT | TT | TT | TT | TT | TT | TT |
|  | AA | AA | AA | AA | AA | AA | AA | AA | AA | AA | AA | AA | AA |
|  | AA | AA | AA | AA | AA | AA | AA | AA | AA | AA | AA | AA | AA |
|  | TT | TT | TT | TT | TT | TT | TT | TT | TT | TT | TT | TT | TT |
|  | AA | AA | AA | AA | AA | AA | AA | AA | AA | AA | AA | AA | AA |
|  | AA | AA | AA | AA | AA | AA | AA | AA | AA | AA | AA | AA | AA |
|  | TT | TT | TT | TT | TT | TT | TT | TT | TT | TT | TT | TT | TT |
|  | TT | TT | TT | TT | TT | TT | TT | TT | TT | TT | TT | TT | TT |
|  | AA | AA | AA | AA | AA | AA | AA | AA | AA | AA | AA | AA | AA |
|  | GG | GG | GG | GG | GG | GG | GG | GG | GG | GG | GG | GG | GG |
|  | TT | TT | TT | TT | TT | TT | TT | TT | TT | TT | TT | TT | TT |
|  | GG | GG | GG | GG | GG | GG | GG | GG | GG | GG | GG | GG | GG |
|  | GG | GG | GG | GG | GG | GG | GG | GG | GG | GG | GG | GG | GG |
|  | GG | GG | GG | GG | GG | GG | GG | GG | GG | GG | GG | GG | GG |
|  | AA | AA | AA | AA | AA | AA | AA | AA | AA | AA | AA | AA | AA |
|  | TT | TT | TT | TT | TT | TT | TT | TT | TT | TT | TT | TT | TT |
|  | AA | AA | AA | AA | AA | AA | AA | AA | AA | AA | AA | AA | AA |
|  | TT | TT | TT | TT | TT | TT | TT | TT | TT | TT | TT | TT | TT |
|  | AA | AA | AA | AA | AA | AA | AA | AA | AA | AA | AA | AA | AA |
|  | AA | AA | AA | AA | AA | AA | AA | AA | AA | AA | AA | AA | AA |
|  | CC | CC | CC | CC | CC | CC | CC | CC | CC | CC | CC | CC | CC |
|  | AA | AA | AA | AA | AA | AA | AA | AA | AA | AA | AA | AA | AA |
|  | AA | AA | AA | AA | AA | AA | AA | AA | AA | AA | AA | AA | AA |
|  | CC | CC | CC | CC | CC | CC | CC | CC | CC | CC | CC | CC | CC |
|  | TT | TT | TT | TT | TT | TT | TT | TT | TT | TT | TT | TT | TT |
|  | CC | CC | CC | CC | CC | CC | CC | CC | CC | CC | CC | CC | CC |
|  | GG | GG | GG | GG | GG | GG | GG | GG | GG | GG | GG | GG | GG |
|  | TT | TT | TT | TT | TT | TT | TT | TT | TT | TT | TT | TT | TT |
|  | CC | CC | CC | CC | CC | CC | CC | CC | CC | CC | CC | CC | CC |
|  | CC | CC | CC | CC | CC | CC | CC | CC | CC | CC | CC | CC | CC |
|  | AA | AA | AA | AA | AA | AA | AA | AA | AA | AA | AA | AA | AA |
|  | GG | GG | GG | GG | GG | GG | GG | GG | GG | GG | GG | GG | GG |
|  | TT | TT | TT | TT | TT | TT | TT | TT | TT | TT | TT | TT | TT |
|  | TT | TT | TT | TT | TT | TT | TT | TT | TT | TT | TT | TT | TT |
|  | TT | TT | TT | TT | TT | TT | TT | TT | TT | TT | TT | TT | TT |
|  | TT | TT | TT | TT | TT | TT | TT | TT | TT | TT | TT | TT | TT |
|  | TT | TT | TT | TT | TT | TT | TT | TT | TT | TT | TT | TT | TT |
|  | AA | AA | AA | AA | AA | AA | AA | AA | AA | AA | AA | AA | AA |
|  | AA | AA | AA | AA | AA | AA | AA | AA | AA | AA | AA | AA | AA |
|  | TT | TT | TT | TT | TT | TT | TT | TT | TT | TT | TT | TT | TT |
|  | AA | AA | AA | AA | AA | AA | AA | AA | AA | AA | AA | AA | AA |
|  | TT | TT | TT | TT | TT | TT | TT | TT | TT | TT | TT | TT | TT |
|  | CC | CC | CC | CC | CC | CC | CC | CC | CC | CC | CC | CC | CC |
|  | TT | TT | TT | TT | TT | TT | TT | TT | TT | TT | TT | TT | TT |
|  | GG | GG | GG | GG | GG | GG | GG | GG | GG | GG | GG | GG | GG |
|  | TT | TT | TT | TT | TT | TT | TT | TT | TT | TT | TT | TT | TT |
|  | GG | GG | GG | GG | GG | GG | GG | GG | GG | GG | GG | GG | GG |
|  | GG | GG | GG | GG | GG | GG | GG | GG | GG | GG | GG | GG | GG |
|  | AA | AA | AA | AA | AA | AA | AA | AA | AA | AA | AA | AA | AA |
|  | CC | CC | CC | CC | CC | CC | CC | CC | CC | CC | CC | CC | CC |
|  | TT | TT | TT | TT | TT | TT | TT | TT | TT | TT | TT | TT | TT |
|  | CC | CC | CC | CC | CC | CC | CC | CC | CC | CC | CC | CC | CC |
|  | GG | GG | GG | GG | GG | GG | GG | GG | GG | GG | GG | GG | GG |
|  | GG | GG | GG | GG | GG | GG | GG | GG | GG | GG | GG | GG | GG |
|  | GG | GG | GG | GG | GG | GG | GG | GG | GG | GG | GG | GG | GG |
|  | AA | AA | AA | AA | AA | AA | AA | AA | AA | AA | AA | AA | AA |
|  | TT | TT | TT | TT | TT | TT | TT | TT | TT | TT | TT | TT | TT |
|  | GG | GG | GG | GG | GG | GG | GG | GG | GG | GG | GG | GG | GG |
|  | AA | AA | AA | AA | AA | AA | AA | AA | AA | AA | AA | AA | AA |
|  | GG | GG | GG | GG | GG | GG | GG | GG | GG | GG | GG | GG | GG |
|  | AA | AA | AA | AA | AA | AA | AA | AA | AA | AA | AA | AA | AA |
|  | TT | TT | TT | TT | TT | TT | TT | TT | TT | TT | TT | TT | TT |
|  | AA | AA | AA | AA | AA | AA | AA | AA | AA | AA | AA | AA | AA |
|  | CC | CC | CC | CC | CC | CC | CC | CC | CC | CC | CC | CC | CC |
|  | CC | CC | CC | CC | CC | CC | CC | CC | CC | CC | CC | CC | CC |
|  | CC | CC | CC | CC | CC | CC | CC | CC | CC | CC | CC | CC | CC |
|  | AA | AA | AA | AA | AA | AA | AA | AA | AA | AA | AA | AA | AA |
|  | AA | AA | AA | AA | AA | AA | AA | AA | AA | AA | AA | AA | AA |
|  | AA | AA | AA | AA | AA | AA | AA | AA | AA | AA | AA | AA | AA |
|  | TT | TT | TT | TT | TT | TT | TT | TT | TT | TT | TT | TT | TT |
|  | TT | TT | TT | TT | TT | TT | TT | TT | TT | TT | TT | TT | TT |
|  | AA | AA | AA | AA | AA | AA | AA | AA | AA | AA | AA | AA | AA |
|  | CC | CC | CC | CC | CC | CC | CC | CC | CC | CC | CC | CC | CC |
|  | TT | TT | TT | TT | TT | TT | TT | TT | TT | TT | TT | TT | TT |
|  | TT | TT | TT | TT | TT | TT | TT | TT | TT | TT | TT | TT | TT |
|  | GG | GG | GG | GG | GG | GG | GG | GG | GG | GG | GG | GG | GG |
|  | CC | CC | CC | CC | CC | CC | CC | CC | CC | CC | CC | CC | CC |
|  | AA | AA | AA | AA | AA | AA | AA | AA | AA | AA | AA | AA | AA |
|  | GG | GG | GG | GG | GG | GG | GG | GG | GG | GG | GG | GG | GG |
|  | TT | TT | TT | TT | TT | TT | TT | TT | TT | TT | TT | TT | TT |
|  | CC | CC | CC | CC | CC | CC | CC | CC | CC | CC | CC | CC | CC |
|  | AA | AA | AA | AA | AA | AA | AA | AA | AA | AA | AA | AA | AA |
|  | AA | AA | AA | AA | AA | AA | AA | AA | AA | AA | AA | AA | AA |
|  | GG | GG | GG | GG | GG | GG | GG | GG | GG | GG | GG | GG | GG |
|  | GG | GG | GG | GG | GG | GG | GG | GG | GG | GG | GG | GG | GG |
|  | CC | CC | CC | CC | CC | CC | CC | CC | CC | CC | CC | CC | CC |
|  | TT | TT | TT | TT | TT | TT | TT | TT | TT | TT | TT | TT | TT |
|  | TT | TT | TT | TT | TT | TT | TT | TT | TT | TT | TT | TT | TT |
|  | TT | TT | TT | TT | TT | TT | TT | TT | TT | TT | TT | TT | TT |
|  | AA | AA | AA | AA | AA | AA | AA | AA | AA | AA | AA | AA | AA |
|  | AA | AA | AA | AA | AA | AA | AA | AA | AA | AA | AA | AA | AA |
|  | AA | AA | AA | AA | AA | AA | AA | AA | AA | AA | AA | AA | AA |
|  | CC | CC | CC | CC | CC | CC | CC | CC | CC | CC | CC | CC | CC |
|  | GG | GG | GG | GG | GG | GG | GG | GG | GG | GG | GG | GG | GG |
|  | GG | GG | GG | GG | GG | GG | GG | GG | GG | GG | GG | GG | GG |
|  | AA | AA | AA | AA | AA | AA | AA | AA | AA | AA | AA | AA | AA |
|  | CC | CC | CC | CC | CC | CC | CC | CC | CC | CC | CC | CC | CC |
|  | TT | TT | TT | TT | TT | TT | TT | TT | TT | TT | TT | TT | TT |
|  | AA | AA | AA | AA | AA | AA | AA | AA | AA | AA | AA | AA | AA |
|  | CC | CC | CC | CC | CC | CC | CC | CC | CC | CC | CC | CC | CC |
|  | AA | AA | AA | AA | AA | AA | AA | AA | AA | AA | AA | AA | AA |
|  | CC | CC | CC | CC | CC | CC | CC | CC | CC | CC | CC | CC | CC |
|  | TT | TT | TT | TT | TT | TT | TT | TT | TT | TT | TT | TT | TT |
|  | AA | AA | AA | AA | AA | AA | AA | AA | AA | AA | AA | AA | AA |
|  | CC | CC | CC | CC | CC | CC | CC | CC | CC | CC | CC | CC | CC |
|  | CC | CC | CC | CC | CC | CC | CC | CC | CC | CC | CC | CC | CC |
|  | TT | TT | TT | TT | TT | TT | TT | TT | TT | TT | TT | TT | TT |
|  | TT | TT | TT | TT | TT | TT | TT | TT | TT | TT | TT | TT | TT |
|  | GG | GG | GG | GG | GG | GG | GG | GG | GG | GG | GG | GG | GG |
|  | CC | CC | CC | CC | CC | CC | CC | CC | CC | CC | CC | CC | CC |
|  | TT | TT | TT | TT | TT | TT | TT | TT | TT | TT | TT | TT | TT |
|  | GG | GG | GG | GG | GG | GG | GG | GG | GG | GG | GG | GG | GG |
|  | AA | AA | AA | AA | AA | AA | AA | AA | AA | AA | AA | AA | AA |
|  | GG | GG | GG | GG | GG | GG | GG | GG | GG | GG | GG | GG | GG |
|  | AA | AA | AA | AA | AA | AA | AA | AA | AA | AA | AA | AA | AA |
|  | GG | GG | GG | GG | GG | GG | GG | GG | GG | GG | GG | GG | GG |
|  | GG | GG | GG | GG | GG | GG | GG | GG | GG | GG | GG | GG | GG |
|  | TT | TT | TT | TT | TT | TT | TT | TT | TT | TT | TT | TT | TT |
|  | CC | CC | CC | CC | CC | CC | CC | CC | CC | CC | CC | CC | CC |
|  | CC | CC | CC | CC | CC | CC | CC | CC | CC | CC | CC | CC | CC |
|  | CC | CC | CC | CC | CC | CC | CC | CC | CC | CC | CC | CC | CC |
|  | CC | CC | CC | CC | CC | CC | CC | CC | CC | CC | CC | CC | CC |
|  | AA | AA | AA | AA | AA | AA | AA | AA | AA | AA | AA | AA | AA |
|  | GG | GG | GG | GG | GG | GG | GG | GG | GG | GG | GG | GG | GG |
|  | GG | GG | GG | GG | GG | GG | GG | GG | GG | GG | GG | GG | GG |
|  | TT | TT | TT | TT | TT | TT | TT | TT | TT | TT | TT | TT | TT |
|  | TT | TT | TT | TT | TT | TT | TT | TT | TT | TT | TT | TT | TT |
|  | TT | TT | TT | TT | TT | TT | TT | TT | TT | TT | TT | TT | TT |
|  | AA | AA | AA | AA | AA | AA | AA | AA | AA | AA | AA | AA | AA |
|  | AA | AA | AA | AA | AA | AA | AA | AA | AA | AA | AA | AA | AA |
|  | AA | AA | AA | AA | AA | AA | AA | AA | AA | AA | AA | AA | AA |
|  | CC | CC | CC | CC | CC | CC | CC | CC | CC | CC | CC | CC | CC |
|  | TT | TT | TT | TT | TT | TT | TT | TT | TT | TT | TT | TT | TT |
|  | TT | TT | TT | TT | TT | TT | TT | TT | TT | TT | TT | TT | TT |
|  | AA | AA | AA | AA | AA | AA | AA | AA | AA | AA | AA | AA | AA |
|  | AA | AA | AA | AA | AA | AA | AA | AA | AA | AA | AA | AA | AA |
|  | AA | AA | AA | AA | AA | AA | AA | AA | AA | AA | AA | AA | AA |
|  | TT | TT | TT | TT | TT | TT | TT | TT | TT | TT | TT | TT | TT |
|  | GG | GG | GG | GG | GG | GG | GG | GG | GG | GG | GG | GG | GG |
|  | AA | AA | AA | AA | AA | AA | AA | AA | AA | AA | AA | AA | AA |
|  | CC | CC | CC | CC | CC | CC | CC | CC | CC | CC | CC | CC | CC |
|  | AA | AA | AA | AA | AA | AA | AA | AA | AA | AA | AA | AA | AA |
|  | GG | GG | GG | GG | GG | GG | GG | GG | GG | GG | GG | GG | GG |
|  | AA | AA | AA | AA | AA | AA | AA | AA | AA | AA | AA | AA | AA |
|  | GG | GG | GG | GG | GG | GG | GG | GG | GG | GG | GG | GG | GG |
|  | AA | AA | AA | AA | AA | AA | AA | AA | AA | AA | AA | AA | AA |
|  | AA | AA | AA | AA | AA | AA | AA | AA | AA | AA | AA | AA | AA |
|  | TT | TT | TT | TT | TT | TT | TT | TT | TT | TT | TT | TT | TT |
|  | AA | AA | AA | AA | AA | AA | AA | AA | AA | AA | AA | AA | AA |
|  | CC | CC | CC | CC | CC | CC | CC | CC | CC | CC | CC | CC | CC |
|  | TT | TT | TT | TT | TT | TT | TT | TT | TT | TT | TT | TT | TT |
|  | TT | TT | TT | TT | TT | TT | TT | TT | TT | TT | TT | TT | TT |
|  | CC | CC | CC | CC | CC | CC | CC | CC | CC | CC | CC | CC | CC |
|  | TT | TT | TT | TT | TT | TT | TT | TT | TT | TT | TT | TT | TT |
|  | GG | GG | GG | GG | GG | GG | GG | GG | GG | GG | GG | GG | GG |
|  | CC | CC | CC | CC | CC | CC | CC | CC | CC | CC | CC | CC | CC |
|  | GG | GG | GG | GG | GG | GG | GG | GG | GG | GG | GG | GG | GG |
|  | TT | TT | TT | TT | TT | TT | TT | TT | TT | TT | TT | TT | TT |
|  | CC | CC | CC | CC | CC | CC | CC | CC | CC | CC | CC | CC | CC |
|  | TT | TT | TT | TT | TT | TT | TT | TT | TT | TT | TT | TT | TT |
|  | AA | AA | AA | AA | AA | AA | AA | AA | AA | AA | AA | AA | AA |
|  | AA | AA | AA | AA | AA | AA | AA | AA | AA | AA | AA | AA | AA |
|  | AA | AA | AA | AA | AA | AA | AA | AA | AA | AA | AA | AA | AA |
|  | AA | AA | AA | AA | AA | AA | AA | AA | AA | AA | AA | AA | AA |
|  | AA | AA | AA | AA | AA | AA | AA | AA | AA | AA | AA | AA | AA |
|  | TT | TT | TT | TT | TT | TT | TT | TT | TT | TT | TT | TT | TT |
|  | TT | TT | TT | TT | TT | TT | TT | TT | TT | TT | TT | TT | TT |
|  | GG | GG | GG | GG | GG | GG | GG | GG | GG | GG | GG | GG | GG |
|  | TT | TT | TT | TT | TT | TT | TT | TT | TT | TT | TT | TT | TT |
|  | CC | CC | CC | CC | CC | CC | CC | TT | TT | CC | CC | CC | CC |
|  | GG | GG | GG | GG | GG | GG | GG | AA | AA | GG | GG | GG | GG |
|  | CC | CC | CC | CC | CC | CC | CC | AA | AA | CC | CC | CC | CC |
|  | CC | CC | CC | CC | CC | CC | CC | TT | TT | CC | CC | CC | CC |
|  | CC | CC | CC | CC | CC | CC | CC | AA | AA | CC | CC | CC | CC |
|  | TT | TT | TT | TT | TT | TT | TT | AA | AA | TT | TT | TT | TT |
|  | AA | AA | AA | AA | AA | AA | AA | CC | CC | AA | AA | AA | AA |
|  | CC | CC | CC | CC | CC | CC | CC | TT | TT | CC | CC | CC | CC |
|  | AA | AA | AA | AA | AA | AA | AA | GG | GG | AA | AA | AA | AA |
|  | TT | TT | TT | TT | TT | TT | TT | TT | TT | TT | TT | TT | TT |
|  | AA | AA | AA | AA | AA | AA | AA | AA | AA | AA | AA | AA | AA |
|  | CC | CC | CC | CC | CC | CC | CC | CC | CC | CC | CC | CC | CC |
|  | TT | TT | TT | TT | TT | TT | TT | TT | TT | TT | TT | TT | TT |
|  | CC | CC | CC | CC | CC | CC | CC | CC | CC | CC | CC | CC | CC |
|  | TT | TT | TT | TT | TT | TT | TT | TT | TT | TT | TT | TT | TT |
|  | GG | GG | GG | GG | GG | GG | GG | GG | GG | GG | GG | GG | GG |
|  | AA | AA | AA | AA | AA | AA | AA | AA | AA | AA | AA | AA | AA |
|  | TT | TT | TT | TT | TT | TT | TT | TT | TT | TT | TT | TT | TT |
|  | GG | GG | GG | GG | GG | GG | GG | GG | GG | GG | GG | GG | GG |
|  | TT | TT | TT | TT | TT | TT | TT | TT | TT | TT | TT | TT | TT |
|  | GG | GG | GG | GG | GG | GG | GG | GG | GG | GG | GG | GG | GG |
|  | TT | TT | TT | TT | TT | TT | TT | TT | TT | TT | TT | TT | TT |
|  | GG | GG | GG | GG | GG | GG | GG | GG | GG | GG | GG | GG | GG |
|  | CC | CC | CC | CC | CC | CC | CC | CC | CC | CC | CC | CC | CC |
|  | TT | TT | TT | TT | TT | TT | TT | TT | TT | TT | TT | TT | TT |
|  | CC | CC | CC | CC | CC | CC | CC | CC | CC | CC | CC | CC | CC |
|  | AA | AA | AA | AA | AA | AA | AA | AA | AA | AA | AA | AA | AA |
|  | GG | GG | GG | GG | GG | GG | GG | GG | GG | GG | GG | GG | GG |
|  | TT | TT | TT | TT | TT | TT | TT | TT | TT | TT | TT | TT | TT |
|  | CC | CC | CC | CC | CC | CC | CC | CC | CC | CC | CC | CC | CC |
|  | CC | CC | CC | CC | CC | CC | CC | CC | CC | CC | CC | CC | CC |
|  | TT | TT | TT | TT | TT | TT | TT | TT | TT | TT | TT | TT | TT |
|  | TT | TT | TT | TT | TT | TT | TT | TT | TT | TT | TT | TT | TT |
|  | GG | GG | GG | GG | GG | GG | GG | GG | GG | GG | GG | GG | GG |
|  | TT | TT | TT | TT | TT | TT | TT | TT | TT | TT | TT | TT | TT |
|  | GG | GG | GG | GG | GG | GG | GG | GG | GG | GG | GG | GG | GG |
|  | CC | CC | CC | CC | CC | CC | CC | CC | CC | CC | CC | CC | CC |
|  | GG | GG | GG | GG | GG | GG | GG | GG | GG | GG | GG | GG | GG |
|  | TT | TT | TT | TT | TT | TT | TT | TT | TT | TT | TT | TT | TT |
|  | CC | CC | CC | CC | CC | CC | CC | CC | CC | CC | CC | CC | CC |
|  | CC | CC | CC | CC | CC | CC | CC | CC | CC | CC | CC | CC | CC |
|  | GG | GG | GG | GG | GG | GG | GG | GG | GG | GG | GG | GG | GG |
|  | TT | TT | TT | TT | TT | TT | TT | TT | TT | TT | TT | TT | TT |
|  | TT | TT | TT | TT | TT | TT | TT | TT | TT | TT | TT | TT | TT |
|  | CC | CC | CC | CC | CC | CC | CC | CC | CC | CC | CC | CC | CC |
|  | AA | AA | AA | AA | AA | AA | AA | AA | AA | AA | AA | AA | AA |
|  | AA | AA | AA | AA | AA | AA | AA | AA | AA | AA | AA | AA | AA |
|  | GG | GG | GG | GG | GG | GG | GG | GG | GG | GG | GG | GG | GG |
|  | GG | GG | GG | GG | GG | GG | GG | GG | GG | GG | GG | GG | GG |
|  | AA | AA | AA | AA | AA | AA | AA | AA | AA | AA | AA | AA | AA |
|  | CC | CC | CC | CC | CC | CC | CC | CC | CC | CC | CC | CC | CC |
|  | AA | AA | AA | AA | AA | AA | AA | AA | AA | AA | AA | AA | AA |
|  | TT | TT | TT | TT | TT | TT | TT | TT | TT | TT | TT | TT | TT |
|  | GG | GG | GG | GG | GG | GG | GG | GG | GG | GG | GG | GG | GG |
|  | TT | TT | TT | TT | TT | TT | TT | TT | TT | TT | TT | TT | TT |
|  | CC | CC | CC | CC | CC | CC | CC | CC | CC | CC | CC | CC | CC |
|  | TT | TT | TT | TT | TT | TT | TT | TT | TT | TT | TT | TT | TT |
|  | GG | GG | GG | GG | GG | GG | GG | GG | GG | GG | GG | GG | GG |
|  | AA | AA | AA | AA | AA | AA | AA | AA | AA | AA | AA | AA | AA |
|  | GG | GG | GG | GG | GG | GG | GG | GG | GG | GG | GG | GG | GG |
|  | AA | AA | AA | AA | AA | AA | AA | AA | AA | AA | AA | AA | AA |
|  | GG | GG | GG | GG | GG | GG | GG | GG | GG | GG | GG | GG | GG |
|  | TT | TT | TT | TT | TT | TT | TT | TT | TT | TT | TT | TT | TT |
|  | AA | AA | AA | AA | AA | AA | AA | AA | AA | AA | AA | AA | AA |
|  | TT | TT | TT | TT | TT | TT | TT | TT | TT | TT | TT | TT | TT |
|  | TT | TT | TT | TT | TT | TT | TT | TT | TT | TT | TT | TT | TT |
|  | CC | CC | CC | CC | CC | CC | CC | CC | CC | CC | CC | CC | CC |
|  | AA | AA | AA | AA | AA | AA | AA | AA | AA | AA | AA | AA | AA |
|  | CC | CC | CC | CC | CC | CC | CC | CC | CC | CC | CC | CC | CC |
|  | GG | GG | GG | GG | GG | GG | GG | GG | GG | GG | GG | GG | GG |
|  | CC | CC | CC | CC | CC | CC | CC | CC | CC | CC | CC | CC | CC |
|  | CC | CC | CC | CC | CC | CC | CC | CC | CC | CC | CC | CC | CC |
|  | AA | AA | AA | AA | AA | AA | AA | AA | AA | AA | AA | AA | AA |
|  | TT | TT | TT | TT | TT | TT | TT | TT | TT | TT | TT | TT | TT |
|  | AA | AA | AA | AA | AA | AA | AA | AA | AA | AA | AA | AA | AA |
|  | TT | TT | TT | TT | TT | TT | TT | TT | TT | TT | TT | TT | TT |
|  | GG | GG | GG | GG | GG | GG | GG | GG | GG | GG | GG | GG | GG |
|  | GG | GG | GG | GG | GG | GG | GG | GG | GG | GG | GG | GG | GG |
|  | CC | CC | CC | CC | CC | CC | CC | CC | CC | CC | CC | CC | CC |
|  | TT | TT | TT | TT | TT | TT | TT | TT | TT | TT | TT | TT | TT |
|  | AA | AA | AA | AA | AA | AA | AA | AA | AA | AA | AA | AA | AA |
|  | GG | GG | GG | GG | GG | GG | GG | GG | GG | GG | GG | GG | GG |
|  | TT | TT | TT | TT | TT | TT | TT | TT | TT | TT | TT | TT | TT |
|  | TT | TT | TT | TT | TT | TT | TT | TT | TT | TT | TT | TT | TT |
|  | CC | CC | CC | CC | CC | CC | CC | CC | CC | CC | CC | CC | CC |
|  | GG | GG | GG | GG | GG | GG | GG | GG | GG | GG | GG | GG | GG |
|  | TT | TT | TT | TT | TT | TT | TT | TT | TT | TT | TT | TT | TT |
|  | AA | AA | AA | AA | AA | AA | AA | AA | AA | AA | AA | AA | AA |
|  | AA | AA | AA | AA | AA | AA | AA | AA | AA | AA | AA | AA | AA |
|  | AA | AA | AA | AA | AA | AA | AA | AA | AA | AA | AA | AA | AA |
|  | TT | TT | TT | AA | AA | TT | TT | TT | TT | TT | TT | TT | TT |
|  | TT | TT | TT | CC | CC | TT | TT | TT | TT | TT | TT | TT | TT |
|  | AA | AA | AA | GG | GG | AA | AA | AA | AA | AA | AA | AA | AA |
|  | AA | AA | AA | GG | GG | AA | AA | AA | AA | AA | AA | AA | AA |
|  | AA | AA | AA | TT | TT | AA | AA | AA | AA | AA | AA | AA | AA |
|  | GG | GG | GG | TT | TT | GG | GG | GG | GG | GG | GG | GG | GG |
|  | TT | TT | TT | CC | CC | TT | TT | TT | TT | TT | TT | TT | TT |
|  | TT | TT | TT | CC | CC | TT | TT | TT | TT | TT | TT | TT | TT |
|  | TT | TT | TT | CC | CC | TT | TT | TT | TT | TT | TT | TT | TT |
|  | TT | TT | TT | CC | CC | TT | TT | TT | TT | TT | TT | TT | TT |
|  | TT | TT | TT | CC | CC | TT | TT | TT | TT | TT | TT | TT | TT |
|  | AA | AA | AA | GG | GG | AA | AA | AA | AA | AA | AA | AA | AA |
|  | AA | AA | AA | GG | GG | AA | AA | AA | AA | AA | AA | AA | AA |
|  | TT | TT | TT | CC | CC | TT | TT | TT | TT | TT | TT | TT | TT |
|  | AA | AA | AA | GG | GG | AA | AA | AA | AA | AA | AA | AA | AA |
|  | CC | CC | CC | TT | TT | CC | CC | CC | CC | CC | CC | CC | CC |
|  | CC | CC | CC | TT | TT | CC | CC | CC | CC | CC | CC | CC | CC |
|  | CC | CC | CC | TT | TT | CC | CC | CC | CC | CC | CC | CC | CC |
|  | CC | CC | CC | TT | TT | CC | CC | CC | CC | CC | CC | CC | CC |
|  | CC | CC | CC | TT | TT | CC | CC | CC | CC | CC | CC | CC | CC |
|  | AA | AA | AA | CC | CC | AA | AA | AA | AA | AA | AA | AA | AA |
|  | CC | CC | CC | TT | TT | CC | CC | CC | CC | CC | CC | CC | CC |
|  | TT | TT | TT | TT | TT | TT | TT | TT | TT | TT | TT | TT | TT |
|  | AA | AA | AA | AA | AA | AA | AA | AA | AA | AA | AA | AA | AA |
|  | AA | AA | AA | AA | AA | AA | AA | AA | AA | AA | AA | AA | AA |
|  | GG | GG | GG | GG | GG | GG | GG | GG | GG | GG | GG | GG | GG |
|  | AA | AA | AA | AA | AA | AA | AA | AA | AA | AA | AA | AA | AA |
|  | CC | CC | CC | CC | CC | CC | CC | CC | CC | CC | CC | CC | CC |
|  | AA | AA | AA | AA | AA | AA | AA | AA | AA | AA | AA | AA | AA |
|  | AA | AA | AA | AA | AA | AA | AA | AA | AA | AA | AA | AA | AA |
|  | CC | CC | CC | CC | CC | CC | CC | CC | CC | CC | CC | CC | CC |
|  | GG | GG | GG | GG | GG | GG | GG | GG | GG | GG | GG | GG | GG |
|  | GG | GG | GG | GG | GG | GG | GG | GG | GG | GG | GG | GG | GG |
|  | TT | TT | TT | TT | TT | TT | TT | TT | TT | TT | TT | TT | TT |
|  | TT | TT | TT | TT | TT | TT | TT | TT | TT | TT | TT | TT | TT |
|  | AA | AA | AA | AA | AA | AA | AA | AA | AA | AA | AA | AA | AA |
|  | GG | GG | GG | GG | GG | GG | GG | GG | GG | GG | GG | GG | GG |
|  | AA | AA | AA | AA | AA | AA | AA | AA | AA | AA | AA | AA | AA |
|  | AA | AA | AA | AA | AA | AA | AA | AA | AA | AA | AA | AA | AA |
|  | AA | AA | AA | AA | AA | AA | AA | AA | AA | AA | AA | AA | AA |
|  | GG | GG | GG | GG | GG | GG | GG | GG | GG | GG | GG | GG | GG |
|  | GG | GG | GG | GG | GG | GG | GG | GG | GG | GG | GG | GG | GG |
|  | GG | GG | GG | GG | GG | GG | GG | GG | GG | GG | GG | GG | GG |
|  | TT | TT | TT | TT | TT | TT | TT | TT | TT | TT | TT | TT | TT |
|  | GG | GG | GG | GG | GG | GG | GG | GG | GG | GG | GG | GG | GG |
|  | TT | TT | TT | TT | TT | TT | TT | TT | TT | TT | TT | TT | TT |
|  | TT | TT | TT | TT | TT | TT | TT | TT | TT | TT | TT | TT | TT |
|  | AA | AA | AA | AA | AA | AA | AA | AA | AA | AA | AA | AA | AA |
|  | AA | AA | AA | AA | AA | AA | AA | AA | AA | AA | AA | AA | AA |
|  | AA | AA | AA | AA | AA | AA | AA | AA | AA | AA | AA | AA | AA |
|  | GG | GG | GG | GG | GG | GG | GG | GG | GG | GG | GG | GG | GG |
|  | TT | TT | TT | TT | TT | TT | TT | TT | TT | TT | TT | TT | TT |
|  | GG | GG | GG | GG | GG | GG | GG | GG | GG | GG | GG | GG | GG |
|  | GG | GG | GG | GG | GG | GG | GG | GG | GG | GG | GG | GG | GG |
|  | TT | TT | TT | TT | TT | TT | TT | TT | TT | TT | TT | TT | TT |
|  | CC | CC | CC | CC | CC | CC | CC | CC | CC | CC | CC | CC | CC |
|  | TT | TT | TT | TT | TT | TT | TT | TT | TT | TT | TT | TT | TT |
|  | TT | TT | TT | TT | TT | TT | TT | TT | TT | TT | TT | TT | TT |
|  | CC | CC | CC | CC | CC | CC | CC | CC | CC | CC | CC | CC | CC |
|  | GG | GG | GG | GG | GG | GG | GG | GG | GG | GG | GG | GG | GG |
|  | CC | CC | CC | CC | CC | CC | CC | CC | CC | CC | CC | CC | CC |
|  | AA | AA | AA | AA | AA | AA | AA | AA | AA | AA | AA | AA | AA |
|  | CC | CC | CC | CC | CC | CC | CC | CC | CC | CC | CC | CC | CC |
|  | TT | TT | TT | TT | TT | TT | TT | TT | TT | TT | TT | TT | TT |
|  | GG | GG | GG | GG | GG | GG | GG | GG | GG | GG | GG | GG | GG |
|  | TT | TT | TT | TT | TT | TT | TT | TT | TT | TT | TT | TT | TT |
|  | TT | TT | TT | TT | TT | TT | TT | TT | TT | TT | TT | TT | TT |
|  | TT | TT | TT | TT | TT | TT | TT | TT | TT | TT | TT | TT | TT |
|  | TT | TT | TT | TT | TT | TT | TT | TT | TT | TT | TT | TT | TT |
|  | AA | AA | AA | AA | AA | AA | AA | AA | AA | AA | AA | AA | AA |
|  | AA | AA | AA | AA | AA | AA | AA | AA | AA | AA | AA | AA | AA |
|  | TT | TT | TT | TT | TT | TT | TT | TT | TT | TT | TT | TT | TT |
|  | GG | GG | GG | GG | GG | GG | GG | GG | GG | GG | GG | GG | GG |
|  | GG | GG | GG | GG | GG | GG | GG | GG | GG | GG | GG | GG | GG |
|  | CC | CC | CC | CC | CC | CC | CC | CC | CC | CC | CC | CC | CC |
|  | CC | CC | CC | CC | CC | CC | CC | CC | CC | CC | CC | CC | CC |
|  | CC | CC | CC | CC | CC | CC | CC | CC | CC | CC | CC | CC | CC |
|  | TT | TT | TT | TT | TT | TT | TT | TT | TT | TT | TT | TT | TT |
|  | AA | AA | AA | AA | AA | AA | AA | AA | AA | AA | AA | AA | AA |
|  | CC | CC | CC | CC | CC | CC | CC | CC | CC | CC | CC | CC | CC |
|  | GG | GG | GG | GG | GG | GG | GG | GG | GG | GG | GG | GG | GG |
|  | AA | AA | AA | AA | AA | AA | AA | AA | AA | AA | AA | AA | AA |
|  | GG | GG | GG | GG | GG | GG | GG | GG | GG | GG | GG | GG | GG |
|  | GG | GG | GG | GG | GG | GG | GG | GG | GG | GG | GG | GG | GG |
|  | AA | AA | AA | AA | AA | AA | AA | AA | AA | AA | AA | AA | AA |
|  | TT | TT | TT | TT | TT | TT | TT | TT | TT | TT | TT | TT | TT |
|  | GG | GG | GG | GG | GG | GG | GG | GG | GG | GG | GG | GG | GG |
|  | TT | TT | TT | TT | TT | TT | TT | TT | TT | TT | TT | TT | TT |
|  | AA | AA | AA | AA | AA | AA | AA | AA | AA | AA | AA | AA | AA |
|  | TT | TT | TT | TT | TT | TT | TT | TT | TT | TT | TT | TT | TT |
|  | GG | GG | GG | GG | GG | GG | GG | GG | GG | GG | GG | GG | GG |
|  | TT | TT | TT | TT | TT | TT | TT | TT | TT | TT | TT | TT | TT |
|  | GG | GG | GG | GG | GG | GG | GG | GG | GG | GG | GG | GG | GG |
|  | TT | TT | TT | TT | TT | TT | TT | TT | TT | TT | TT | TT | TT |
|  | GG | GG | GG | GG | GG | GG | GG | GG | GG | GG | GG | GG | GG |
|  | AA | AA | AA | AA | AA | AA | AA | AA | AA | AA | AA | AA | AA |
|  | AA | AA | AA | AA | AA | AA | AA | AA | AA | AA | AA | AA | AA |
|  | GG | GG | GG | GG | GG | GG | GG | GG | GG | GG | GG | GG | GG |
|  | AA | AA | AA | AA | AA | AA | AA | AA | AA | AA | AA | AA | AA |
|  | TT | TT | TT | TT | TT | TT | TT | TT | TT | TT | TT | TT | TT |
|  | CC | CC | CC | CC | CC | CC | CC | CC | CC | CC | CC | CC | CC |
|  | TT | TT | TT | TT | TT | TT | TT | TT | TT | TT | TT | TT | TT |
|  | TT | TT | TT | TT | TT | TT | TT | TT | TT | TT | TT | TT | TT |
|  | CC | CC | CC | CC | CC | CC | CC | CC | CC | CC | CC | CC | CC |
|  | CC | CC | CC | CC | CC | CC | CC | CC | CC | CC | CC | CC | CC |
|  | GG | GG | GG | GG | GG | GG | GG | GG | GG | GG | GG | GG | GG |
|  | GG | GG | GG | GG | GG | GG | GG | GG | GG | GG | GG | GG | GG |
|  | AA | AA | AA | AA | AA | AA | AA | AA | AA | AA | AA | AA | AA |
|  | AA | AA | AA | AA | AA | AA | AA | AA | AA | AA | AA | AA | AA |
|  | CC | CC | CC | CC | CC | CC | CC | CC | CC | CC | CC | CC | CC |
|  | CC | CC | CC | CC | CC | CC | CC | CC | CC | CC | CC | CC | CC |
|  | CC | CC | CC | CC | CC | CC | CC | CC | CC | CC | CC | CC | CC |
|  | GG | GG | GG | GG | GG | GG | GG | GG | GG | GG | GG | GG | GG |
|  | TT | TT | TT | TT | TT | TT | TT | TT | TT | TT | TT | TT | TT |
|  | AA | AA | AA | AA | AA | AA | AA | AA | AA | AA | AA | AA | AA |
|  | TT | TT | TT | TT | TT | TT | TT | TT | TT | TT | TT | TT | TT |
|  | CC | CC | CC | CC | CC | CC | CC | CC | CC | CC | CC | CC | CC |
|  | GG | GG | GG | GG | GG | GG | GG | GG | GG | GG | GG | GG | GG |
|  | GG | GG | GG | GG | GG | GG | GG | GG | GG | GG | GG | GG | GG |
|  | CC | CC | CC | CC | CC | CC | CC | CC | CC | CC | CC | CC | CC |
|  | GG | GG | GG | GG | GG | GG | GG | GG | GG | GG | GG | GG | GG |
|  | AA | AA | AA | AA | AA | AA | AA | AA | AA | AA | AA | AA | AA |
|  | GG | GG | GG | GG | GG | GG | GG | GG | GG | GG | GG | GG | GG |
|  | CC | CC | CC | CC | CC | CC | CC | CC | CC | CC | CC | CC | CC |
|  | CC | CC | CC | CC | CC | CC | CC | CC | CC | CC | CC | CC | CC |
|  | AA | AA | AA | AA | AA | AA | AA | AA | AA | AA | AA | AA | AA |
|  | AA | AA | AA | AA | AA | AA | AA | AA | AA | AA | AA | AA | AA |
|  | TT | TT | TT | TT | TT | TT | TT | CC | CC | TT | TT | TT | TT |
|  | CC | CC | CC | CC | CC | CC | CC | TT | TT | CC | CC | CC | CC |
|  | GG | GG | GG | GG | GG | GG | GG | CC | CC | GG | GG | GG | GG |
|  | CC | CC | CC | CC | CC | CC | CC | TT | TT | CC | CC | CC | CC |
|  | AA | AA | AA | AA | AA | AA | AA | GG | GG | AA | AA | AA | AA |
|  | GG | GG | GG | GG | GG | GG | GG | TT | TT | GG | GG | GG | GG |
|  | TT | TT | TT | TT | TT | TT | TT | TT | TT | TT | TT | TT | TT |
|  | GG | GG | GG | GG | GG | GG | GG | GG | GG | GG | GG | GG | GG |
|  | TT | TT | TT | TT | TT | TT | TT | TT | TT | TT | TT | TT | TT |
|  | TT | TT | TT | TT | TT | TT | TT | TT | TT | TT | TT | TT | TT |
|  | CC | CC | CC | CC | CC | CC | CC | CC | CC | CC | CC | CC | CC |
|  | AA | AA | AA | AA | AA | AA | AA | AA | AA | AA | AA | AA | AA |
|  | AA | AA | AA | AA | AA | AA | AA | AA | AA | AA | AA | AA | AA |
|  | CC | CC | CC | CC | CC | CC | CC | CC | CC | CC | CC | CC | CC |
|  | AA | AA | AA | AA | AA | AA | AA | AA | AA | AA | AA | AA | AA |
|  | GG | GG | GG | GG | GG | GG | GG | GG | GG | GG | GG | GG | GG |
|  | AA | AA | AA | AA | AA | AA | AA | AA | AA | AA | AA | AA | AA |
|  | GG | GG | GG | GG | GG | GG | GG | GG | GG | GG | GG | GG | GG |
|  | GG | GG | GG | GG | GG | GG | GG | GG | GG | GG | GG | GG | GG |
|  | AA | AA | AA | AA | AA | AA | AA | AA | AA | AA | AA | AA | AA |
|  | TT | TT | TT | TT | TT | TT | TT | TT | TT | TT | TT | TT | TT |
|  | TT | TT | TT | TT | TT | TT | TT | TT | TT | TT | TT | TT | TT |
|  | CC | CC | CC | CC | CC | CC | CC | CC | CC | CC | CC | CC | CC |
|  | CC | CC | CC | CC | CC | CC | CC | CC | CC | CC | CC | CC | CC |
|  | TT | TT | TT | TT | TT | TT | TT | TT | TT | TT | TT | TT | TT |
|  | TT | TT | TT | TT | TT | TT | TT | TT | TT | TT | TT | TT | TT |
|  | AA | AA | AA | AA | AA | AA | AA | AA | AA | AA | AA | AA | AA |
|  | CC | CC | CC | CC | CC | CC | CC | CC | CC | CC | CC | CC | CC |
|  | GG | GG | GG | GG | GG | GG | GG | GG | GG | GG | GG | GG | GG |
|  | CC | CC | CC | CC | CC | CC | CC | CC | CC | CC | CC | CC | CC |
|  | CC | CC | CC | CC | CC | CC | CC | CC | CC | CC | CC | CC | CC |
|  | CC | CC | CC | CC | CC | CC | CC | CC | CC | CC | CC | CC | CC |
|  | AA | AA | AA | AA | AA | AA | AA | AA | AA | AA | AA | AA | AA |
|  | AA | AA | AA | AA | AA | AA | AA | AA | AA | AA | AA | AA | AA |
|  | AA | AA | AA | AA | AA | AA | AA | AA | AA | AA | AA | AA | AA |
|  | GG | GG | GG | GG | GG | GG | GG | GG | GG | GG | GG | GG | GG |
|  | TT | TT | TT | TT | TT | TT | TT | TT | TT | TT | TT | TT | TT |
|  | AA | AA | AA | AA | AA | AA | AA | AA | AA | AA | AA | AA | AA |
|  | AA | TT | TT | AA | AA | AA | AA | AA | AA | AA | AA | AA | AA |
|  | CC | GG | GG | CC | CC | CC | CC | CC | CC | CC | CC | CC | CC |
|  | AA | TT | TT | AA | AA | AA | AA | AA | AA | AA | AA | AA | AA |
|  | AA | TT | TT | AA | AA | AA | AA | AA | AA | AA | AA | AA | AA |
|  | CC | AA | AA | CC | CC | CC | CC | CC | CC | CC | CC | CC | CC |
|  | GG | GG | GG | GG | GG | GG | GG | GG | GG | GG | GG | GG | GG |
|  | AA | AA | AA | AA | AA | AA | AA | AA | AA | AA | AA | AA | AA |
|  | TT | TT | TT | TT | TT | TT | TT | TT | TT | TT | TT | TT | TT |
|  | AA | AA | AA | AA | AA | AA | AA | AA | AA | AA | AA | AA | AA |
|  | AA | AA | AA | AA | AA | AA | AA | AA | AA | AA | AA | AA | AA |
|  | CC | CC | CC | CC | CC | CC | CC | CC | CC | CC | CC | CC | CC |
|  | GG | GG | GG | GG | GG | GG | GG | GG | GG | GG | GG | GG | GG |
|  | CC | CC | CC | CC | CC | CC | CC | CC | CC | CC | CC | CC | CC |
|  | GG | GG | GG | GG | GG | GG | GG | GG | GG | GG | GG | GG | GG |
|  | GG | GG | GG | GG | GG | GG | GG | GG | GG | GG | GG | GG | GG |
|  | AA | AA | AA | AA | AA | AA | AA | AA | AA | AA | AA | AA | AA |
|  | CC | CC | CC | CC | CC | CC | CC | CC | CC | CC | CC | CC | CC |
|  | CC | CC | CC | CC | CC | CC | CC | CC | CC | CC | CC | CC | CC |
|  | TT | TT | TT | TT | TT | TT | TT | TT | TT | TT | TT | TT | TT |
|  | AA | AA | AA | AA | AA | AA | AA | AA | AA | AA | AA | AA | AA |
|  | TT | TT | TT | TT | TT | TT | TT | TT | TT | TT | TT | TT | TT |
|  | AA | AA | AA | AA | AA | AA | AA | AA | AA | AA | AA | AA | AA |
|  | GG | GG | GG | GG | GG | GG | GG | GG | GG | GG | GG | GG | GG |
|  | GG | GG | GG | GG | GG | GG | GG | GG | GG | GG | GG | GG | GG |
|  | AA | AA | AA | AA | AA | AA | AA | AA | AA | AA | AA | AA | AA |
|  | GG | GG | GG | GG | GG | GG | GG | GG | GG | GG | GG | GG | GG |
|  | AA | AA | AA | AA | AA | AA | AA | AA | AA | AA | AA | AA | AA |
|  | TT | TT | TT | TT | TT | TT | TT | TT | TT | TT | TT | TT | TT |
|  | GG | GG | GG | GG | GG | GG | GG | GG | GG | GG | GG | GG | GG |
|  | CC | CC | CC | CC | CC | CC | CC | CC | CC | CC | CC | CC | CC |
|  | TT | TT | TT | TT | TT | TT | TT | TT | TT | TT | TT | TT | TT |
|  | TT | TT | TT | TT | TT | TT | TT | TT | TT | TT | TT | TT | TT |
|  | AA | AA | AA | AA | AA | AA | AA | AA | AA | AA | AA | AA | AA |
|  | TT | TT | TT | TT | TT | TT | TT | TT | TT | TT | TT | TT | TT |
|  | GG | GG | GG | GG | GG | GG | GG | GG | GG | GG | GG | GG | GG |
|  | GG | GG | GG | GG | GG | GG | GG | GG | GG | GG | GG | GG | GG |
|  | AA | AA | AA | AA | AA | AA | AA | AA | AA | AA | AA | AA | AA |
|  | GG | GG | GG | GG | GG | GG | GG | GG | GG | GG | GG | GG | GG |
|  | AA | AA | AA | AA | AA | AA | AA | AA | AA | AA | AA | AA | AA |
|  | TT | TT | TT | TT | TT | TT | TT | TT | TT | TT | TT | TT | TT |
|  | TT | TT | TT | TT | TT | TT | TT | TT | TT | TT | TT | TT | TT |
|  | CC | CC | CC | CC | CC | CC | CC | CC | CC | CC | CC | CC | CC |
|  | TT | TT | TT | TT | TT | TT | TT | TT | TT | TT | TT | TT | TT |
|  | GG | GG | GG | GG | GG | GG | GG | GG | GG | GG | GG | GG | GG |
|  | CC | CC | CC | CC | CC | CC | CC | CC | CC | CC | CC | CC | CC |
|  | AA | AA | AA | AA | AA | AA | AA | AA | AA | AA | AA | AA | AA |
|  | TT | TT | TT | TT | TT | TT | TT | TT | TT | TT | TT | TT | TT |
|  | GG | GG | GG | GG | GG | GG | GG | GG | GG | GG | GG | GG | GG |
|  | CC | CC | CC | CC | CC | CC | CC | CC | CC | CC | CC | CC | CC |
|  | TT | TT | TT | TT | TT | TT | TT | TT | TT | TT | TT | TT | TT |
|  | CC | CC | CC | CC | CC | CC | CC | CC | CC | CC | CC | CC | CC |
|  | CC | CC | CC | CC | CC | CC | CC | CC | CC | CC | CC | CC | CC |
|  | TT | TT | TT | TT | TT | TT | TT | TT | TT | TT | TT | TT | TT |
|  | CC | CC | CC | CC | CC | CC | CC | CC | CC | CC | CC | CC | CC |
|  | GG | GG | GG | GG | GG | GG | GG | GG | GG | GG | GG | GG | GG |
|  | AA | AA | AA | AA | AA | AA | AA | AA | AA | AA | AA | AA | AA |
|  | AA | AA | AA | AA | AA | AA | AA | AA | AA | AA | AA | AA | AA |
|  | CC | CC | CC | CC | CC | CC | CC | CC | CC | CC | CC | CC | CC |
|  | TT | TT | TT | TT | TT | TT | TT | TT | TT | TT | TT | TT | TT |
|  | GG | GG | GG | GG | GG | GG | GG | GG | GG | GG | GG | GG | GG |
|  | TT | TT | TT | TT | TT | TT | TT | TT | TT | TT | TT | TT | TT |
|  | CC | CC | CC | CC | CC | CC | CC | CC | CC | CC | CC | CC | CC |
|  | CC | CC | CC | CC | CC | CC | CC | CC | CC | CC | CC | CC | CC |
|  | CC | CC | CC | CC | CC | CC | CC | CC | CC | CC | CC | CC | CC |
|  | CC | CC | CC | CC | CC | CC | CC | CC | CC | CC | CC | CC | CC |
|  | CC | CC | CC | CC | CC | CC | CC | CC | CC | CC | CC | CC | CC |
|  | GG | GG | GG | GG | GG | GG | GG | GG | GG | GG | GG | GG | GG |
|  | AA | AA | AA | AA | AA | AA | AA | AA | AA | AA | AA | AA | AA |
|  | CC | CC | CC | CC | CC | CC | CC | CC | CC | CC | CC | CC | CC |
|  | TT | TT | TT | TT | TT | TT | TT | TT | TT | TT | TT | TT | TT |
|  | TT | TT | TT | TT | TT | TT | TT | TT | TT | TT | TT | TT | TT |
|  | GG | GG | GG | GG | GG | GG | GG | GG | GG | GG | GG | GG | GG |
|  | AA | AA | AA | AA | AA | AA | AA | AA | AA | AA | AA | AA | AA |
|  | GG | GG | GG | GG | GG | GG | GG | GG | GG | GG | GG | GG | GG |
|  | CC | CC | CC | CC | CC | CC | CC | CC | CC | CC | CC | CC | CC |
|  | TT | TT | TT | TT | TT | TT | TT | TT | TT | TT | TT | TT | TT |
|  | CC | CC | CC | CC | CC | CC | CC | CC | CC | CC | CC | CC | CC |
|  | TT | TT | TT | TT | TT | TT | TT | TT | TT | TT | TT | TT | TT |
|  | TT | TT | TT | TT | TT | TT | TT | TT | TT | TT | TT | TT | TT |
|  | TT | TT | TT | TT | TT | TT | TT | TT | TT | TT | TT | TT | TT |
|  | TT | TT | TT | TT | TT | TT | TT | TT | TT | TT | TT | TT | TT |
|  | TT | TT | TT | TT | TT | TT | TT | TT | TT | TT | TT | TT | TT |
|  | AA | AA | AA | AA | AA | AA | AA | AA | AA | AA | AA | AA | AA |
|  | AA | AA | AA | AA | AA | AA | AA | AA | AA | AA | AA | AA | AA |
|  | AA | AA | AA | AA | AA | AA | AA | AA | AA | AA | AA | AA | AA |
|  | GG | GG | GG | GG | GG | GG | GG | GG | GG | GG | GG | GG | GG |
|  | GG | GG | GG | GG | GG | GG | GG | GG | GG | GG | GG | GG | GG |
|  | GG | GG | GG | GG | GG | GG | GG | GG | GG | GG | GG | GG | GG |
|  | CC | CC | CC | CC | CC | CC | CC | CC | CC | CC | CC | CC | CC |
|  | GG | GG | GG | GG | GG | GG | GG | GG | GG | GG | GG | GG | GG |
|  | GG | GG | GG | GG | GG | GG | GG | GG | GG | GG | GG | GG | GG |
|  | GG | GG | GG | GG | GG | GG | GG | GG | GG | GG | GG | GG | GG |
|  | GG | GG | GG | GG | GG | GG | GG | GG | GG | GG | GG | GG | GG |
|  | TT | TT | TT | TT | TT | TT | TT | TT | TT | TT | TT | TT | TT |
|  | GG | GG | GG | GG | GG | GG | GG | GG | GG | GG | GG | GG | GG |
|  | AA | AA | AA | AA | AA | AA | AA | AA | AA | AA | AA | AA | AA |
|  | TT | TT | TT | TT | TT | TT | TT | TT | TT | TT | TT | TT | TT |
|  | CC | CC | CC | CC | CC | CC | CC | CC | CC | CC | CC | CC | CC |
|  | CC | CC | CC | CC | CC | CC | CC | CC | CC | CC | CC | CC | CC |
|  | GG | GG | GG | GG | GG | GG | GG | GG | GG | GG | GG | GG | GG |
|  | CC | CC | CC | CC | CC | CC | CC | CC | CC | CC | CC | CC | CC |
|  | TT | TT | TT | TT | TT | TT | TT | TT | TT | TT | TT | TT | TT |
|  | AA | AA | AA | AA | AA | AA | AA | AA | AA | AA | AA | AA | AA |
|  | TT | TT | TT | TT | TT | TT | TT | TT | TT | TT | TT | TT | TT |
|  | TT | TT | TT | TT | TT | TT | TT | TT | TT | TT | TT | TT | TT |
|  | AA | AA | AA | AA | AA | AA | AA | AA | AA | AA | AA | AA | AA |
|  | AA | AA | AA | AA | AA | AA | AA | AA | AA | AA | AA | AA | AA |
|  | AA | AA | AA | AA | AA | AA | AA | AA | AA | AA | AA | AA | AA |
|  | TT | TT | TT | TT | TT | TT | TT | TT | TT | TT | TT | TT | TT |
|  | CC | CC | CC | CC | CC | CC | CC | CC | CC | CC | CC | CC | CC |
|  | TT | TT | TT | TT | TT | TT | TT | TT | TT | TT | TT | TT | TT |
|  | GG | GG | GG | GG | GG | GG | GG | GG | GG | GG | GG | GG | GG |
|  | GG | GG | GG | GG | GG | GG | GG | GG | GG | GG | GG | GG | GG |
|  | TT | TT | TT | TT | TT | TT | TT | TT | TT | TT | TT | TT | TT |
|  | GG | GG | GG | GG | GG | GG | GG | GG | GG | GG | GG | GG | GG |
|  | GG | GG | GG | GG | GG | GG | GG | GG | GG | GG | GG | GG | GG |
|  | GG | GG | GG | GG | GG | GG | GG | GG | GG | GG | GG | GG | GG |
|  | CC | CC | CC | CC | CC | CC | CC | CC | CC | CC | CC | CC | CC |
|  | GG | GG | GG | GG | GG | GG | GG | GG | GG | GG | GG | GG | GG |
|  | CC | CC | CC | CC | CC | CC | CC | CC | CC | CC | CC | CC | CC |
|  | GG | GG | GG | GG | GG | GG | GG | GG | GG | GG | GG | GG | GG |
|  | GG | GG | GG | GG | GG | GG | GG | GG | GG | GG | GG | GG | GG |
|  | GG | GG | GG | GG | GG | GG | GG | GG | GG | GG | GG | GG | GG |
|  | CC | CC | CC | CC | CC | CC | CC | CC | CC | CC | CC | CC | CC |
|  | TT | TT | TT | TT | TT | TT | TT | TT | TT | TT | TT | TT | TT |
|  | TT | TT | TT | TT | TT | TT | TT | TT | TT | TT | TT | TT | TT |
|  | CC | CC | CC | CC | CC | CC | CC | CC | CC | CC | CC | CC | CC |
|  | GG | GG | GG | GG | GG | GG | GG | GG | GG | GG | GG | GG | GG |
|  | TT | TT | TT | TT | TT | TT | TT | TT | TT | TT | TT | TT | TT |
|  | TT | TT | TT | TT | TT | TT | TT | TT | TT | TT | TT | TT | TT |
|  | TT | TT | TT | TT | TT | TT | TT | TT | TT | TT | TT | TT | TT |
|  | CC | TT | TT | CC | CC | CC | CC | CC | CC | CC | CC | CC | CC |
|  | GG | AA | AA | GG | GG | GG | GG | GG | GG | GG | GG | GG | GG |
|  | TT | CC | CC | TT | TT | TT | TT | TT | TT | TT | TT | TT | TT |
|  | AA | AA | AA | AA | AA | AA | AA | AA | AA | AA | AA | AA | AA |
|  | AA | AA | AA | AA | AA | AA | AA | AA | AA | AA | AA | AA | AA |
|  | GG | GG | GG | GG | GG | GG | GG | GG | GG | GG | GG | GG | GG |
|  | CC | CC | CC | CC | CC | CC | CC | CC | CC | CC | CC | CC | CC |
|  | AA | AA | AA | AA | AA | AA | AA | AA | AA | AA | AA | AA | AA |
|  | CC | CC | CC | CC | CC | CC | CC | CC | CC | CC | CC | CC | CC |
|  | TT | TT | TT | TT | TT | TT | TT | TT | TT | TT | TT | TT | TT |
|  | AA | AA | AA | AA | AA | AA | AA | AA | AA | AA | AA | AA | AA |
|  | TT | TT | TT | TT | TT | TT | TT | TT | TT | TT | TT | TT | TT |
|  | TT | TT | TT | TT | TT | TT | TT | TT | TT | TT | TT | TT | TT |
|  | GG | GG | GG | GG | GG | GG | GG | GG | GG | GG | GG | GG | GG |
|  | CC | CC | CC | CC | CC | CC | CC | CC | CC | CC | CC | CC | CC |
|  | AA | AA | AA | AA | AA | AA | AA | AA | AA | AA | AA | AA | AA |
|  | TT | TT | TT | TT | TT | TT | TT | TT | TT | TT | TT | TT | TT |
|  | TT | TT | TT | TT | TT | TT | TT | CC | CC | TT | TT | TT | TT |
|  | CC | CC | CC | CC | CC | CC | CC | TT | TT | CC | CC | CC | CC |
|  | CC | CC | CC | CC | CC | CC | CC | TT | TT | CC | CC | CC | CC |
|  | TT | TT | TT | TT | TT | TT | TT | CC | CC | TT | TT | TT | TT |
|  | TT | TT | TT | TT | TT | TT | TT | CC | CC | TT | TT | TT | TT |
|  | TT | TT | TT | TT | TT | TT | TT | CC | CC | TT | TT | TT | TT |
|  | GG | GG | GG | GG | GG | GG | GG | GG | GG | GG | GG | GG | GG |
|  | AA | AA | AA | AA | AA | AA | AA | AA | AA | AA | AA | AA | AA |
|  | AA | AA | AA | AA | AA | AA | AA | AA | AA | AA | AA | AA | AA |
|  | AA | AA | AA | AA | AA | AA | AA | AA | AA | AA | AA | AA | AA |
|  | CC | CC | CC | CC | CC | CC | CC | CC | CC | CC | CC | CC | CC |
|  | AA | AA | AA | AA | AA | AA | AA | AA | AA | AA | AA | AA | AA |
|  | TT | TT | TT | TT | TT | TT | TT | TT | TT | TT | TT | TT | TT |
|  | CC | CC | CC | CC | CC | CC | CC | CC | CC | CC | CC | CC | CC |
|  | AA | AA | AA | AA | AA | AA | AA | AA | AA | AA | AA | AA | AA |
|  | GG | GG | GG | GG | GG | GG | GG | GG | GG | GG | GG | GG | GG |
|  | TT | TT | TT | TT | TT | TT | TT | TT | TT | TT | TT | TT | TT |
|  | CC | CC | CC | CC | CC | CC | CC | CC | CC | CC | CC | CC | CC |
|  | AA | AA | AA | AA | AA | AA | AA | AA | AA | AA | AA | AA | AA |
|  | TT | TT | TT | TT | TT | TT | TT | TT | TT | TT | TT | TT | TT |
|  | AA | AA | AA | AA | AA | AA | AA | AA | AA | AA | AA | AA | AA |
|  | TT | TT | TT | TT | TT | TT | TT | TT | TT | TT | TT | TT | TT |
|  | AA | AA | AA | AA | AA | AA | AA | AA | AA | AA | AA | AA | AA |
|  | GG | GG | GG | GG | GG | GG | GG | GG | GG | GG | GG | GG | GG |
|  | AA | AA | AA | AA | AA | AA | AA | AA | AA | AA | AA | AA | AA |
|  | GG | GG | GG | GG | GG | GG | GG | GG | GG | GG | GG | GG | GG |
|  | AA | AA | AA | AA | AA | AA | AA | AA | AA | AA | AA | AA | AA |
|  | CC | CC | CC | CC | CC | CC | CC | CC | CC | CC | CC | CC | CC |
|  | CC | CC | CC | CC | CC | CC | CC | CC | CC | CC | CC | CC | CC |
|  | AA | AA | AA | AA | AA | AA | AA | AA | AA | AA | AA | AA | AA |
|  | AA | AA | AA | AA | AA | AA | AA | AA | AA | AA | AA | AA | AA |
|  | CC | CC | CC | CC | CC | CC | CC | CC | CC | CC | CC | CC | CC |
|  | CC | CC | CC | CC | CC | CC | CC | CC | CC | CC | CC | CC | CC |
|  | CC | CC | CC | CC | CC | CC | CC | CC | CC | CC | CC | CC | CC |
|  | GG | GG | GG | GG | GG | GG | GG | GG | GG | GG | GG | GG | GG |
|  | TT | TT | TT | TT | TT | TT | TT | TT | TT | TT | TT | TT | TT |
|  | GG | GG | GG | GG | GG | GG | GG | GG | GG | GG | GG | GG | GG |
|  | CC | CC | CC | CC | CC | CC | CC | CC | CC | CC | CC | CC | CC |
|  | CC | CC | CC | CC | CC | CC | CC | CC | CC | CC | CC | CC | CC |
|  | TT | TT | TT | TT | TT | TT | TT | TT | TT | TT | TT | TT | TT |
|  | AA | AA | AA | AA | AA | AA | AA | AA | AA | AA | AA | AA | AA |
|  | CC | CC | CC | CC | CC | CC | CC | CC | CC | CC | CC | CC | CC |
|  | TT | TT | TT | TT | TT | TT | TT | TT | TT | TT | TT | TT | TT |
|  | GG | GG | GG | GG | GG | GG | GG | GG | GG | GG | GG | GG | GG |
|  | AA | AA | AA | AA | AA | AA | AA | AA | AA | AA | AA | AA | AA |
|  | GG | GG | GG | GG | GG | GG | GG | GG | GG | GG | GG | GG | GG |
|  | TT | TT | TT | TT | TT | TT | TT | TT | TT | TT | TT | TT | TT |
|  | TT | TT | TT | TT | TT | TT | TT | TT | TT | TT | TT | TT | TT |
|  | TT | TT | TT | TT | TT | CC | CC | TT | TT | TT | TT | TT | TT |
|  | CC | CC | CC | CC | CC | AA | AA | CC | CC | CC | CC | CC | CC |
|  | CC | CC | CC | CC | CC | TT | TT | CC | CC | CC | CC | CC | CC |
|  | TT | TT | TT | TT | TT | CC | CC | TT | TT | TT | TT | TT | TT |
|  | GG | GG | GG | GG | GG | CC | CC | GG | GG | GG | GG | GG | GG |
|  | AA | AA | AA | AA | AA | CC | CC | AA | AA | AA | AA | AA | AA |
|  | GG | GG | GG | GG | GG | AA | AA | GG | GG | GG | GG | GG | GG |
|  | AA | AA | AA | AA | AA | GG | GG | AA | AA | AA | AA | AA | AA |
|  | GG | GG | GG | GG | GG | TT | TT | GG | GG | GG | GG | GG | GG |
|  | CC | CC | CC | CC | CC | CC | CC | CC | CC | CC | CC | CC | CC |
|  | AA | AA | AA | AA | AA | AA | AA | AA | AA | AA | AA | AA | AA |
|  | GG | GG | GG | GG | GG | GG | GG | GG | GG | GG | GG | GG | GG |
|  | CC | CC | CC | CC | CC | CC | CC | CC | CC | CC | CC | CC | CC |
|  | CC | CC | CC | CC | CC | CC | CC | CC | CC | CC | CC | CC | CC |
|  | CC | CC | CC | CC | CC | CC | CC | CC | CC | CC | CC | CC | CC |
|  | CC | CC | CC | CC | CC | CC | CC | CC | CC | CC | CC | CC | CC |
|  | CC | CC | CC | CC | CC | CC | CC | CC | CC | CC | CC | CC | CC |
|  | AA | AA | AA | AA | AA | AA | AA | AA | AA | AA | AA | AA | AA |
|  | TT | TT | TT | TT | TT | TT | TT | TT | TT | TT | TT | TT | TT |
|  | AA | AA | AA | AA | AA | AA | AA | AA | AA | AA | AA | AA | AA |
|  | TT | TT | TT | TT | TT | TT | TT | TT | TT | TT | TT | TT | TT |
|  | CC | CC | CC | CC | CC | CC | CC | CC | CC | CC | CC | CC | CC |
|  | CC | CC | CC | CC | CC | CC | CC | CC | CC | CC | CC | CC | CC |
|  | CC | CC | CC | CC | CC | CC | CC | CC | CC | CC | CC | CC | CC |
|  | TT | TT | TT | TT | TT | TT | TT | TT | TT | TT | TT | TT | TT |
|  | AA | AA | AA | AA | AA | AA | AA | AA | AA | AA | AA | AA | AA |
|  | AA | AA | AA | AA | AA | AA | AA | AA | AA | AA | AA | AA | AA |
|  | AA | AA | AA | AA | AA | AA | AA | AA | AA | AA | AA | AA | AA |
|  | AA | AA | AA | AA | AA | AA | AA | AA | AA | AA | AA | AA | AA |
|  | GG | GG | GG | GG | GG | GG | GG | GG | GG | GG | GG | AA | GG |
|  | CC | CC | CC | CC | CC | CC | CC | CC | CC | CC | CC | TT | CC |
|  | GG | GG | GG | GG | GG | GG | GG | GG | GG | GG | GG | AA | GG |
|  | CC | CC | CC | CC | CC | CC | CC | TT | CC | CC | CC | TT | CC |
|  | AA | AA | AA | AA | AA | AA | AA | CC | AA | AA | AA | CC | AA |
|  | TT | TT | TT | GG | TT | TT | TT | GG | TT | TT | TT | GG | TT |
|  | TT | TT | TT | CC | TT | CC | TT | CC | TT | TT | TT | CC | TT |
|  | AA | AA | AA | GG | AA | GG | AA | GG | AA | AA | AA | GG | AA |
|  | GG | AA | GG | AA | GG | AA | GG | AA | GG | GG | GG | AA | GG |
|  | AA | GG | AA | GG | AA | GG | AA | GG | AA | AA | AA | GG | AA |
|  | CC | TT | CC | TT | CC | TT | CC | TT | CC | TT | CC | TT | CC |
|  | TT | CC | TT | CC | TT | CC | TT | CC | TT | CC | TT | CC | TT |
|  | AA | TT | AA | TT | AA | TT | AA | TT | AA | TT | AA | TT | AA |
|  | AA | GG | AA | GG | AA | GG | AA | GG | AA | GG | AA | GG | AA |
|  | GG | TT | GG | TT | GG | TT | GG | TT | GG | TT | GG | TT | GG |
|  | TT | CC | TT | CC | TT | CC | TT | CC | TT | CC | TT | CC | TT |
|  | CC | TT | CC | TT | CC | TT | CC | TT | CC | TT | CC | TT | CC |
|  | GG | AA | GG | AA | GG | AA | GG | AA | GG | AA | GG | AA | GG |
|  | CC | TT | CC | TT | CC | TT | CC | TT | CC | TT | CC | TT | CC |
|  | AA | TT | AA | TT | AA | TT | AA | TT | AA | TT | AA | TT | AA |
|  | GG | AA | GG | AA | GG | AA | GG | AA | GG | AA | GG | AA | GG |
|  | GG | AA | GG | AA | GG | AA | GG | AA | GG | AA | GG | AA | GG |
|  | TT | TT | TT | CC | TT | TT | TT | CC | TT | CC | TT | CC | TT |
|  | TT | TT | TT | GG | TT | TT | TT | GG | TT | GG | TT | GG | TT |
|  | TT | TT | TT | CC | TT | TT | TT | CC | TT | CC | TT | CC | TT |
|  | AA | AA | AA | GG | AA | AA | AA | GG | AA | AA | AA | GG | AA |
|  | TT | TT | TT | CC | TT | TT | TT | CC | TT | TT | TT | CC | TT |
|  | CC | CC | CC | TT | CC | CC | CC | TT | CC | CC | CC | TT | CC |
|  | AA | AA | AA | AA | AA | AA | AA | TT | AA | AA | AA | TT | AA |
|  | AA | AA | AA | AA | AA | AA | AA | AA | AA | AA | AA | TT | AA |
|  | GG | GG | GG | GG | GG | GG | GG | GG | GG | GG | GG | AA | GG |
|  | GG | GG | GG | GG | GG | GG | GG | GG | GG | GG | GG | AA | GG |
|  | TT | TT | TT | TT | TT | TT | TT | TT | TT | TT | TT | TT | TT |
|  | TT | TT | TT | TT | TT | TT | TT | TT | TT | TT | TT | TT | TT |
|  | AA | AA | AA | AA | AA | AA | AA | AA | AA | AA | AA | AA | AA |
|  | CC | CC | CC | CC | CC | CC | CC | CC | CC | CC | CC | CC | CC |
|  | TT | TT | TT | TT | TT | TT | TT | TT | TT | TT | TT | TT | TT |
|  | CC | CC | CC | CC | CC | CC | CC | CC | CC | CC | CC | CC | CC |
|  | TT | TT | TT | TT | TT | TT | TT | TT | TT | TT | TT | TT | TT |
|  | CC | CC | CC | CC | CC | CC | CC | CC | CC | CC | CC | CC | CC |
|  | CC | CC | CC | CC | CC | CC | CC | CC | CC | CC | CC | CC | CC |
|  | AA | AA | AA | AA | AA | AA | AA | AA | AA | AA | AA | AA | AA |
|  | GG | GG | GG | GG | GG | GG | GG | GG | GG | GG | GG | GG | GG |
|  | AA | AA | AA | AA | AA | AA | AA | AA | AA | AA | AA | AA | AA |
|  | TT | TT | TT | TT | TT | TT | TT | TT | TT | TT | TT | TT | TT |
|  | AA | AA | AA | AA | AA | AA | AA | AA | AA | AA | AA | AA | AA |
|  | CC | CC | CC | CC | CC | CC | CC | CC | CC | CC | CC | CC | CC |
|  | TT | TT | TT | TT | TT | TT | TT | TT | TT | TT | TT | TT | TT |
|  | GG | GG | GG | GG | GG | GG | GG | GG | GG | GG | GG | GG | GG |
|  | CC | CC | CC | CC | CC | CC | CC | CC | CC | CC | CC | CC | CC |
|  | AA | AA | AA | AA | AA | AA | AA | AA | AA | AA | AA | AA | AA |
|  | TT | TT | TT | TT | TT | TT | TT | TT | TT | TT | TT | TT | TT |
|  | AA | AA | AA | AA | AA | AA | AA | AA | AA | AA | AA | AA | AA |
|  | AA | AA | AA | AA | AA | AA | AA | AA | AA | AA | AA | AA | AA |
|  | AA | AA | AA | AA | AA | AA | AA | AA | AA | AA | AA | AA | AA |
|  | GG | GG | GG | GG | GG | GG | GG | GG | GG | GG | GG | GG | GG |
|  | AA | AA | AA | AA | AA | AA | AA | AA | AA | AA | AA | AA | AA |
|  | GG | GG | GG | GG | GG | GG | GG | GG | GG | GG | GG | GG | GG |
|  | AA | AA | AA | AA | AA | AA | AA | AA | AA | AA | AA | AA | AA |
|  | TT | TT | TT | TT | TT | TT | TT | TT | TT | TT | TT | TT | TT |
|  | TT | TT | TT | TT | TT | TT | TT | TT | TT | TT | TT | TT | TT |
|  | AA | AA | AA | AA | AA | AA | AA | AA | AA | AA | AA | AA | AA |
|  | GG | GG | GG | GG | GG | GG | GG | GG | GG | GG | GG | GG | GG |
|  | TT | TT | TT | TT | TT | TT | TT | TT | TT | TT | TT | TT | TT |
|  | TT | TT | TT | TT | TT | TT | TT | TT | TT | TT | TT | TT | TT |
|  | AA | AA | AA | AA | AA | AA | AA | AA | AA | AA | AA | AA | AA |
|  | AA | AA | AA | AA | AA | AA | AA | AA | AA | AA | AA | AA | AA |
|  | AA | AA | AA | AA | AA | AA | AA | AA | AA | AA | AA | AA | AA |
|  | AA | AA | AA | AA | AA | AA | AA | AA | AA | AA | AA | AA | AA |
|  | GG | GG | GG | GG | GG | GG | GG | GG | GG | GG | GG | GG | GG |
|  | TT | TT | TT | TT | TT | TT | TT | TT | TT | TT | TT | TT | TT |
|  | AA | AA | AA | AA | AA | AA | AA | AA | AA | AA | AA | AA | AA |
|  | AA | AA | AA | AA | AA | AA | AA | AA | AA | AA | AA | AA | AA |
|  | CC | CC | CC | CC | CC | CC | CC | CC | CC | CC | CC | CC | CC |
|  | GG | GG | GG | GG | GG | GG | GG | GG | GG | GG | GG | GG | GG |
|  | CC | CC | CC | CC | CC | CC | CC | CC | CC | CC | CC | CC | CC |
|  | AA | AA | AA | AA | AA | AA | AA | AA | AA | AA | AA | AA | AA |
|  | TT | TT | TT | TT | TT | TT | TT | TT | TT | TT | TT | TT | TT |
|  | TT | AA | AA | TT | TT | TT | TT | TT | TT | TT | TT | TT | TT |
|  | TT | CC | CC | TT | TT | TT | TT | TT | TT | TT | TT | TT | TT |
|  | TT | CC | CC | TT | TT | TT | TT | TT | TT | TT | TT | TT | TT |
|  | TT | CC | CC | TT | TT | TT | TT | TT | TT | TT | TT | TT | TT |
|  | CC | CC | CC | CC | CC | CC | CC | CC | CC | CC | CC | TT | TT |
|  | CC | CC | CC | CC | CC | CC | CC | CC | CC | CC | CC | TT | TT |
|  | TT | TT | TT | TT | TT | TT | TT | TT | TT | TT | TT | CC | CC |
|  | CC | CC | CC | CC | CC | CC | CC | CC | CC | CC | CC | TT | TT |
|  | CC | CC | CC | CC | CC | CC | CC | CC | CC | CC | CC | GG | GG |
|  | AA | AA | AA | AA | AA | AA | AA | AA | AA | AA | AA | AA | AA |
|  | CC | CC | CC | CC | CC | CC | CC | CC | CC | CC | CC | CC | CC |
|  | CC | CC | CC | CC | CC | CC | CC | CC | CC | CC | CC | CC | CC |
|  | CC | CC | CC | CC | CC | CC | CC | CC | CC | CC | CC | CC | CC |
|  | CC | CC | CC | CC | CC | CC | CC | CC | CC | CC | CC | CC | CC |
|  | GG | GG | GG | GG | GG | GG | GG | GG | GG | GG | GG | GG | GG |
|  | TT | TT | TT | TT | TT | TT | TT | TT | TT | TT | TT | TT | TT |
|  | CC | CC | CC | CC | CC | CC | CC | CC | CC | CC | CC | CC | CC |
|  | CC | CC | CC | CC | CC | CC | CC | CC | CC | CC | CC | CC | CC |
|  | TT | TT | TT | TT | TT | TT | TT | TT | TT | TT | TT | TT | TT |
|  | GG | GG | GG | GG | GG | GG | GG | GG | GG | GG | GG | GG | GG |
|  | TT | TT | TT | TT | TT | TT | TT | TT | TT | TT | TT | TT | TT |
|  | AA | AA | AA | AA | AA | AA | AA | AA | AA | AA | AA | AA | AA |
|  | GG | GG | GG | GG | GG | GG | GG | GG | GG | GG | GG | GG | GG |
|  | CC | CC | CC | CC | CC | CC | CC | CC | CC | CC | CC | CC | CC |
|  | TT | TT | TT | TT | TT | TT | TT | TT | TT | TT | TT | TT | TT |
|  | AA | AA | AA | AA | AA | AA | AA | AA | AA | AA | AA | AA | AA |
|  | AA | AA | AA | AA | AA | AA | AA | AA | AA | AA | AA | AA | AA |
|  | TT | TT | TT | TT | TT | TT | TT | TT | TT | TT | TT | TT | TT |
|  | TT | TT | TT | TT | TT | TT | TT | TT | TT | TT | TT | TT | TT |
|  | GG | GG | GG | GG | GG | GG | GG | GG | GG | GG | GG | GG | GG |
|  | GG | GG | GG | GG | GG | GG | GG | GG | GG | GG | GG | GG | GG |
|  | CC | CC | CC | CC | CC | CC | CC | CC | CC | CC | CC | CC | CC |
|  | CC | CC | CC | CC | CC | CC | CC | CC | CC | CC | CC | CC | CC |
|  | TT | TT | TT | TT | TT | TT | TT | TT | TT | TT | TT | TT | TT |
|  | CC | CC | CC | CC | CC | CC | CC | CC | CC | CC | CC | CC | CC |
|  | CC | CC | CC | CC | CC | CC | CC | CC | CC | CC | CC | CC | CC |
|  | TT | TT | TT | TT | TT | TT | TT | TT | TT | TT | TT | TT | TT |
|  | GG | GG | GG | AA | AA | GG | GG | GG | GG | GG | GG | GG | GG |
|  | CC | CC | CC | GG | GG | CC | CC | CC | CC | CC | CC | CC | CC |
|  | TT | TT | TT | GG | GG | TT | TT | TT | TT | TT | TT | TT | TT |
|  | TT | TT | TT | CC | CC | TT | TT | TT | TT | TT | TT | TT | TT |
|  | AA | AA | AA | GG | GG | AA | AA | AA | AA | AA | AA | AA | AA |
|  | TT | TT | TT | CC | CC | TT | TT | TT | TT | TT | TT | TT | TT |
|  | AA | AA | AA | GG | GG | AA | AA | AA | AA | AA | AA | AA | AA |
|  | TT | TT | TT | CC | CC | TT | TT | TT | TT | TT | TT | TT | TT |
|  | AA | AA | AA | AA | AA | AA | AA | AA | AA | AA | AA | AA | AA |
|  | TT | TT | TT | TT | TT | TT | TT | TT | TT | TT | TT | TT | TT |
|  | TT | TT | TT | TT | TT | TT | TT | TT | TT | TT | TT | TT | TT |
|  | CC | CC | CC | CC | CC | CC | CC | CC | CC | CC | CC | CC | CC |
|  | AA | AA | AA | AA | AA | AA | AA | AA | AA | AA | AA | AA | AA |
|  | GG | GG | GG | GG | GG | GG | GG | GG | GG | GG | GG | GG | GG |
|  | AA | AA | AA | AA | AA | AA | AA | AA | AA | AA | AA | AA | AA |
|  | CC | CC | CC | CC | CC | CC | CC | CC | CC | CC | CC | CC | CC |
|  | TT | TT | TT | TT | TT | TT | TT | TT | TT | TT | TT | TT | TT |
|  | CC | CC | CC | CC | CC | CC | CC | CC | CC | CC | CC | CC | CC |
|  | GG | GG | GG | GG | GG | GG | GG | GG | GG | GG | GG | GG | GG |
|  | AA | AA | AA | AA | AA | AA | AA | GG | GG | AA | AA | AA | AA |
|  | GG | GG | GG | GG | GG | GG | GG | AA | AA | GG | GG | GG | GG |
|  | AA | AA | AA | AA | AA | AA | AA | GG | GG | AA | AA | AA | AA |
|  | CC | CC | CC | CC | CC | CC | CC | TT | TT | CC | CC | CC | CC |
|  | AA | AA | AA | AA | AA | AA | AA | GG | GG | AA | AA | AA | AA |
|  | CC | CC | CC | CC | CC | CC | CC | GG | GG | CC | CC | CC | CC |
|  | AA | AA | AA | AA | AA | AA | AA | AA | AA | AA | AA | AA | AA |
|  | TT | TT | TT | TT | TT | TT | TT | TT | TT | TT | TT | TT | TT |
|  | TT | TT | TT | TT | TT | TT | TT | TT | TT | TT | TT | TT | TT |
|  | CC | CC | CC | CC | CC | CC | CC | CC | CC | CC | CC | CC | CC |
|  | AA | AA | AA | AA | AA | AA | AA | AA | AA | AA | AA | AA | AA |
|  | CC | CC | CC | CC | CC | CC | CC | CC | CC | CC | CC | CC | CC |
|  | AA | AA | AA | AA | AA | AA | AA | AA | AA | AA | AA | AA | AA |
|  | CC | CC | CC | CC | CC | CC | CC | CC | CC | CC | CC | CC | CC |
|  | AA | AA | AA | AA | AA | AA | AA | AA | AA | AA | AA | AA | AA |
|  | AA | AA | AA | AA | AA | AA | AA | AA | AA | AA | AA | AA | AA |
|  | TT | TT | TT | TT | TT | TT | TT | TT | TT | TT | TT | TT | TT |
|  | AA | AA | AA | AA | AA | AA | AA | AA | AA | AA | AA | AA | AA |
|  | GG | GG | GG | GG | GG | GG | GG | GG | GG | GG | GG | GG | GG |
|  | GG | GG | GG | GG | GG | GG | GG | GG | GG | GG | GG | GG | GG |
|  | TT | TT | TT | TT | TT | TT | TT | TT | TT | TT | TT | TT | TT |
|  | GG | GG | GG | GG | GG | GG | GG | GG | GG | GG | GG | GG | GG |
|  | AA | AA | AA | AA | AA | AA | AA | AA | AA | AA | AA | AA | AA |
|  | CC | CC | CC | CC | CC | CC | CC | CC | CC | CC | CC | CC | CC |
|  | AA | AA | AA | AA | AA | AA | AA | AA | AA | AA | AA | AA | AA |
|  | GG | GG | GG | GG | GG | GG | GG | GG | GG | GG | GG | GG | GG |
|  | GG | GG | GG | GG | GG | GG | GG | GG | GG | GG | GG | GG | GG |
|  | GG | GG | GG | GG | GG | GG | GG | GG | GG | GG | GG | GG | GG |
|  | GG | GG | GG | GG | GG | GG | GG | GG | GG | GG | GG | GG | GG |
|  | AA | AA | AA | AA | AA | AA | AA | AA | AA | AA | AA | AA | AA |
|  | GG | GG | GG | GG | GG | GG | GG | GG | GG | GG | GG | GG | GG |
|  | GG | GG | GG | GG | GG | GG | GG | GG | GG | GG | GG | GG | GG |
|  | CC | CC | CC | CC | CC | CC | CC | CC | CC | CC | CC | CC | CC |
|  | AA | AA | AA | AA | AA | AA | AA | AA | AA | AA | AA | AA | AA |
|  | GG | GG | GG | GG | GG | GG | GG | GG | GG | GG | GG | GG | GG |
|  | AA | AA | AA | AA | AA | AA | AA | AA | AA | AA | AA | AA | AA |
|  | AA | AA | AA | AA | AA | AA | AA | AA | AA | AA | AA | AA | AA |
|  | CC | CC | CC | CC | CC | CC | CC | CC | CC | CC | CC | CC | CC |
|  | CC | CC | CC | CC | CC | CC | CC | CC | CC | CC | CC | CC | CC |
|  | TT | TT | TT | TT | TT | TT | TT | TT | TT | TT | TT | TT | TT |
|  | TT | TT | TT | TT | TT | TT | TT | TT | TT | TT | TT | TT | TT |
|  | CC | CC | CC | CC | CC | CC | CC | CC | CC | CC | CC | CC | CC |
|  | GG | GG | GG | GG | GG | GG | GG | GG | GG | GG | GG | GG | GG |
|  | AA | AA | AA | AA | AA | AA | AA | AA | AA | AA | AA | AA | AA |
|  | CC | CC | CC | CC | CC | CC | CC | CC | CC | CC | CC | CC | CC |
|  | GG | GG | GG | GG | GG | GG | GG | GG | GG | GG | GG | GG | GG |
|  | TT | TT | TT | TT | TT | TT | TT | CC | CC | TT | TT | TT | TT |
|  | TT | TT | TT | TT | TT | TT | TT | AA | AA | TT | TT | TT | TT |
|  | GG | GG | GG | GG | GG | GG | GG | AA | AA | GG | GG | GG | GG |
|  | TT | TT | TT | TT | TT | TT | TT | AA | AA | TT | TT | TT | TT |
|  | AA | AA | AA | AA | AA | AA | AA | TT | TT | AA | AA | AA | AA |
|  | AA | AA | AA | AA | AA | AA | AA | GG | GG | AA | AA | AA | AA |
|  | GG | GG | GG | GG | GG | GG | GG | AA | AA | GG | GG | GG | GG |
|  | CC | CC | CC | CC | CC | CC | CC | TT | TT | CC | CC | CC | CC |
|  | AA | AA | AA | AA | AA | AA | AA | GG | GG | AA | AA | AA | AA |
|  | GG | GG | GG | GG | GG | GG | GG | GG | GG | GG | GG | GG | GG |
|  | AA | AA | AA | AA | AA | AA | AA | AA | AA | AA | AA | AA | AA |
|  | CC | CC | CC | CC | CC | CC | CC | CC | CC | CC | CC | CC | CC |
|  | GG | GG | GG | GG | GG | GG | GG | GG | GG | GG | GG | GG | GG |
|  | CC | CC | CC | CC | CC | CC | CC | CC | CC | CC | CC | CC | CC |
|  | TT | TT | TT | TT | TT | TT | TT | TT | TT | TT | TT | TT | TT |
|  | AA | AA | AA | AA | AA | AA | AA | AA | AA | AA | AA | AA | AA |
|  | TT | TT | TT | TT | TT | TT | TT | TT | TT | TT | TT | TT | TT |
|  | GG | GG | GG | GG | GG | GG | GG | GG | GG | GG | GG | GG | GG |
|  | TT | TT | TT | TT | TT | TT | TT | TT | TT | TT | TT | TT | TT |
|  | CC | CC | CC | CC | CC | CC | CC | CC | CC | CC | CC | CC | CC |
|  | GG | GG | GG | GG | GG | GG | GG | GG | GG | GG | GG | GG | GG |
|  | AA | AA | AA | AA | AA | AA | AA | AA | AA | AA | AA | AA | AA |
|  | CC | CC | CC | CC | CC | CC | CC | CC | CC | CC | CC | CC | CC |
|  | AA | AA | AA | AA | AA | AA | AA | AA | AA | AA | AA | AA | AA |
|  | AA | AA | AA | AA | AA | AA | AA | AA | AA | AA | AA | AA | AA |
|  | CC | CC | CC | CC | CC | CC | CC | CC | CC | CC | CC | CC | CC |
|  | TT | TT | TT | TT | TT | TT | TT | TT | TT | TT | TT | TT | TT |
|  | TT | TT | TT | TT | TT | TT | TT | TT | TT | TT | TT | TT | TT |
|  | CC | CC | CC | CC | CC | CC | CC | CC | CC | CC | CC | CC | CC |
|  | AA | AA | AA | AA | AA | AA | AA | AA | AA | AA | AA | AA | AA |
|  | AA | AA | AA | AA | AA | AA | AA | AA | AA | AA | AA | AA | AA |
|  | GG | GG | GG | GG | GG | GG | GG | GG | GG | GG | GG | GG | GG |
|  | AA | AA | AA | AA | AA | AA | AA | AA | AA | AA | AA | AA | AA |
|  | AA | AA | AA | AA | AA | AA | AA | AA | AA | AA | AA | AA | AA |
|  | TT | TT | TT | TT | TT | TT | TT | TT | TT | TT | TT | TT | TT |
|  | GG | GG | GG | GG | GG | GG | GG | GG | GG | GG | GG | GG | GG |
|  | AA | AA | AA | AA | AA | AA | AA | AA | AA | AA | AA | AA | AA |
|  | GG | GG | GG | GG | GG | GG | GG | GG | GG | GG | GG | GG | GG |
|  | TT | TT | TT | TT | TT | TT | TT | TT | TT | TT | TT | TT | TT |
|  | TT | TT | TT | TT | TT | TT | TT | TT | TT | TT | TT | TT | TT |
|  | AA | AA | AA | AA | AA | AA | AA | AA | AA | AA | AA | AA | AA |
|  | AA | AA | AA | AA | AA | AA | AA | AA | AA | AA | AA | AA | AA |
|  | TT | TT | TT | TT | TT | TT | TT | TT | TT | TT | TT | TT | TT |
|  | CC | CC | CC | CC | CC | CC | CC | CC | CC | CC | CC | CC | CC |
|  | AA | AA | AA | AA | AA | AA | AA | AA | AA | AA | AA | AA | AA |
|  | TT | TT | TT | TT | TT | TT | TT | TT | TT | TT | TT | TT | TT |
|  | TT | TT | TT | TT | TT | TT | TT | TT | TT | TT | TT | TT | TT |
|  | CC | CC | CC | CC | CC | CC | CC | CC | CC | CC | CC | CC | CC |
|  | CC | CC | CC | CC | CC | CC | CC | CC | CC | CC | CC | CC | CC |
|  | CC | CC | CC | CC | CC | CC | CC | CC | CC | CC | CC | CC | CC |
|  | AA | AA | AA | AA | AA | AA | AA | AA | AA | AA | AA | AA | AA |
|  | AA | AA | AA | AA | AA | AA | AA | AA | AA | AA | AA | AA | AA |
|  | AA | AA | AA | AA | AA | AA | AA | AA | AA | AA | AA | AA | AA |
|  | AA | AA | AA | AA | AA | AA | AA | AA | AA | AA | AA | AA | AA |
|  | AA | AA | AA | AA | AA | AA | AA | AA | AA | AA | AA | AA | AA |
|  | AA | AA | AA | AA | AA | AA | AA | AA | AA | AA | AA | AA | AA |
|  | CC | CC | CC | CC | CC | CC | CC | CC | CC | CC | CC | CC | CC |
|  | GG | GG | GG | GG | GG | GG | GG | GG | GG | GG | GG | GG | GG |
|  | CC | CC | CC | CC | CC | CC | CC | CC | CC | CC | CC | CC | CC |
|  | GG | GG | GG | GG | GG | GG | GG | GG | GG | GG | GG | GG | GG |
|  | TT | TT | TT | TT | TT | TT | TT | TT | TT | TT | TT | TT | TT |
|  | CC | CC | CC | CC | CC | CC | CC | CC | CC | CC | CC | CC | CC |
|  | GG | GG | GG | GG | GG | GG | GG | GG | GG | GG | GG | GG | GG |
|  | AA | AA | AA | AA | AA | AA | AA | AA | AA | GG | GG | AA | AA |
|  | TT | TT | TT | TT | TT | TT | TT | TT | TT | CC | CC | TT | TT |
|  | AA | AA | AA | AA | AA | AA | AA | AA | AA | GG | GG | AA | AA |
|  | TT | TT | TT | TT | TT | TT | TT | TT | TT | CC | CC | TT | TT |
|  | GG | GG | GG | GG | GG | GG | GG | GG | GG | GG | GG | GG | GG |
|  | CC | CC | CC | CC | CC | CC | CC | CC | CC | CC | CC | CC | CC |
|  | CC | CC | CC | CC | CC | CC | CC | CC | CC | CC | CC | CC | CC |
|  | GG | GG | GG | GG | GG | GG | GG | GG | GG | GG | GG | GG | GG |
|  | TT | TT | TT | TT | TT | TT | TT | TT | TT | TT | TT | TT | TT |
|  | AA | AA | AA | AA | AA | AA | AA | AA | AA | AA | AA | AA | AA |
|  | CC | CC | CC | CC | CC | CC | CC | CC | CC | CC | CC | CC | CC |
|  | CC | CC | CC | CC | CC | CC | CC | CC | CC | CC | CC | CC | CC |
|  | AA | AA | AA | AA | AA | AA | AA | AA | AA | AA | AA | AA | AA |
|  | GG | GG | GG | GG | GG | GG | GG | GG | GG | GG | GG | GG | GG |
|  | TT | TT | TT | TT | TT | TT | TT | TT | TT | TT | TT | TT | TT |
|  | AA | AA | AA | AA | AA | AA | AA | AA | AA | AA | AA | AA | AA |
|  | AA | AA | AA | AA | AA | AA | AA | AA | AA | AA | AA | AA | AA |
|  | AA | AA | AA | AA | AA | AA | AA | AA | AA | AA | AA | AA | AA |
|  | CC | CC | CC | CC | CC | CC | CC | CC | CC | CC | CC | CC | CC |
|  | GG | GG | GG | GG | GG | GG | GG | GG | GG | GG | GG | GG | GG |
|  | CC | CC | CC | CC | CC | CC | CC | CC | CC | CC | CC | CC | CC |
|  | TT | TT | TT | TT | TT | TT | TT | TT | TT | TT | TT | TT | TT |
|  | GG | GG | GG | GG | GG | GG | GG | GG | GG | GG | GG | GG | GG |
|  | CC | CC | CC | CC | CC | CC | CC | CC | CC | CC | CC | CC | CC |
|  | TT | TT | TT | TT | TT | TT | TT | TT | TT | TT | TT | TT | TT |
|  | GG | GG | GG | GG | GG | GG | GG | GG | GG | GG | GG | GG | GG |
|  | TT | TT | TT | TT | TT | TT | TT | TT | TT | TT | TT | TT | TT |
|  | TT | TT | TT | TT | TT | TT | TT | TT | TT | TT | TT | TT | TT |
|  | CC | CC | CC | CC | CC | CC | CC | CC | CC | CC | CC | CC | CC |
|  | GG | GG | GG | GG | GG | GG | GG | GG | GG | GG | GG | GG | GG |
|  | TT | TT | TT | TT | TT | TT | TT | TT | TT | TT | TT | TT | TT |
|  | AA | AA | AA | AA | AA | AA | AA | AA | AA | AA | AA | AA | AA |
|  | AA | AA | AA | AA | AA | AA | AA | AA | AA | AA | AA | AA | AA |
|  | CC | CC | CC | CC | CC | CC | CC | CC | CC | CC | CC | CC | CC |
|  | GG | GG | GG | GG | GG | GG | GG | GG | GG | GG | GG | GG | GG |
|  | TT | TT | TT | TT | TT | TT | TT | TT | TT | TT | TT | TT | TT |
|  | TT | TT | TT | TT | TT | TT | TT | TT | TT | TT | TT | TT | TT |
|  | GG | GG | GG | GG | GG | GG | GG | GG | GG | GG | GG | GG | GG |
|  | GG | GG | GG | GG | GG | GG | GG | GG | GG | GG | GG | GG | GG |
|  | AA | AA | AA | AA | AA | AA | AA | AA | AA | AA | AA | AA | AA |
|  | GG | GG | GG | GG | GG | GG | GG | GG | GG | GG | GG | GG | GG |
|  | GG | GG | GG | GG | GG | GG | GG | GG | GG | GG | GG | GG | GG |
|  | GG | GG | GG | GG | GG | GG | GG | GG | GG | GG | GG | GG | GG |
|  | AA | AA | AA | AA | AA | AA | AA | AA | AA | AA | AA | AA | AA |
|  | GG | GG | GG | GG | GG | GG | GG | GG | GG | GG | GG | GG | GG |
|  | TT | TT | TT | TT | TT | TT | TT | TT | TT | TT | TT | TT | TT |
|  | TT | TT | TT | TT | TT | TT | TT | TT | TT | TT | TT | TT | TT |
|  | CC | CC | CC | CC | CC | CC | CC | CC | CC | CC | CC | CC | CC |
|  | CC | CC | CC | CC | CC | CC | CC | CC | CC | CC | CC | CC | CC |
|  | GG | GG | GG | GG | GG | GG | GG | GG | GG | GG | GG | GG | GG |
|  | TT | TT | TT | TT | TT | TT | TT | TT | TT | TT | TT | TT | TT |
|  | AA | AA | AA | AA | AA | AA | AA | AA | AA | AA | AA | AA | AA |
|  | CC | CC | CC | CC | CC | CC | CC | CC | CC | CC | CC | CC | CC |
|  | CC | CC | CC | CC | CC | CC | CC | CC | CC | CC | CC | CC | CC |
|  | AA | AA | AA | AA | AA | AA | AA | AA | AA | AA | AA | AA | AA |
|  | TT | TT | TT | TT | TT | TT | TT | TT | TT | TT | TT | TT | TT |
|  | AA | AA | AA | AA | AA | AA | AA | AA | AA | AA | AA | AA | AA |
|  | AA | AA | AA | AA | AA | AA | AA | AA | AA | AA | AA | AA | AA |
|  | AA | AA | AA | AA | AA | AA | AA | AA | AA | AA | AA | AA | AA |
|  | AA | AA | AA | AA | AA | AA | AA | AA | AA | AA | AA | AA | AA |
|  | GG | GG | GG | GG | GG | GG | GG | GG | GG | GG | GG | GG | GG |
|  | AA | AA | AA | AA | AA | AA | AA | AA | AA | AA | AA | AA | AA |
|  | AA | AA | AA | AA | AA | AA | AA | AA | AA | AA | AA | AA | AA |
|  | AA | AA | AA | AA | AA | AA | AA | AA | AA | AA | AA | AA | AA |
|  | TT | TT | TT | TT | TT | CC | CC | TT | TT | TT | TT | TT | TT |
|  | AA | AA | AA | AA | AA | GG | GG | AA | AA | AA | AA | AA | AA |
|  | TT | TT | TT | TT | TT | CC | CC | TT | TT | TT | TT | TT | TT |
|  | TT | TT | TT | TT | TT | TT | TT | TT | TT | TT | TT | TT | TT |
|  | GG | GG | GG | GG | GG | GG | GG | GG | GG | GG | GG | GG | GG |
|  | AA | AA | AA | AA | AA | AA | AA | AA | AA | AA | AA | AA | AA |
|  | AA | AA | AA | AA | AA | AA | AA | AA | AA | AA | AA | AA | AA |
|  | TT | TT | TT | TT | TT | TT | TT | TT | TT | TT | TT | TT | TT |
|  | GG | GG | GG | GG | GG | GG | GG | GG | GG | GG | GG | GG | GG |
|  | AA | AA | AA | AA | AA | AA | AA | AA | AA | AA | AA | AA | AA |
|  | TT | TT | TT | TT | TT | TT | TT | TT | TT | TT | TT | TT | TT |
|  | AA | AA | AA | AA | AA | AA | AA | AA | AA | AA | AA | AA | AA |
|  | CC | CC | CC | CC | CC | CC | CC | CC | CC | CC | CC | CC | CC |
|  | TT | TT | TT | TT | TT | TT | TT | TT | TT | TT | TT | TT | TT |
|  | TT | TT | TT | TT | TT | TT | TT | TT | TT | TT | TT | TT | TT |
|  | TT | TT | TT | TT | TT | TT | TT | TT | TT | TT | TT | TT | TT |
|  | AA | AA | AA | AA | AA | AA | AA | AA | AA | AA | AA | AA | AA |
|  | AA | AA | AA | AA | AA | AA | AA | AA | AA | AA | AA | AA | AA |
|  | GG | GG | GG | GG | GG | GG | GG | GG | GG | GG | GG | GG | GG |
|  | TT | TT | TT | TT | TT | TT | TT | TT | TT | TT | TT | TT | TT |
|  | GG | GG | GG | GG | GG | GG | GG | GG | GG | GG | GG | GG | GG |
|  | AA | AA | AA | AA | AA | AA | AA | AA | AA | AA | AA | AA | AA |
|  | CC | CC | CC | CC | CC | CC | CC | CC | CC | CC | CC | CC | CC |
|  | TT | TT | TT | TT | TT | TT | TT | TT | TT | TT | TT | TT | TT |
|  | GG | GG | GG | GG | GG | GG | GG | GG | GG | GG | GG | GG | GG |
|  | GG | GG | GG | GG | GG | GG | GG | GG | GG | GG | GG | GG | GG |
|  | GG | GG | GG | GG | GG | GG | GG | GG | GG | GG | GG | GG | GG |
|  | TT | TT | TT | TT | TT | TT | TT | TT | TT | TT | TT | TT | TT |
|  | TT | TT | TT | TT | TT | TT | TT | TT | TT | TT | TT | TT | TT |
|  | CC | CC | CC | CC | CC | CC | CC | CC | CC | CC | CC | CC | CC |
|  | CC | CC | CC | CC | CC | CC | CC | CC | CC | CC | CC | CC | CC |
|  | GG | GG | GG | GG | GG | GG | GG | GG | GG | GG | GG | GG | GG |
|  | GG | GG | GG | GG | GG | GG | GG | GG | GG | GG | GG | GG | GG |
|  | GG | GG | GG | GG | GG | GG | GG | GG | GG | GG | GG | GG | GG |
|  | CC | CC | CC | CC | CC | CC | CC | CC | CC | CC | CC | CC | CC |
|  | CC | CC | CC | CC | CC | CC | CC | CC | CC | CC | CC | CC | CC |
|  | TT | TT | TT | TT | TT | TT | TT | TT | TT | TT | TT | TT | TT |
|  | AA | AA | AA | AA | AA | AA | AA | AA | AA | AA | AA | AA | AA |
|  | CC | CC | CC | CC | CC | CC | CC | CC | CC | CC | CC | CC | CC |
|  | GG | GG | GG | GG | GG | GG | GG | GG | GG | GG | GG | GG | GG |
|  | CC | CC | CC | CC | CC | CC | CC | CC | CC | CC | CC | CC | CC |
|  | GG | GG | GG | GG | GG | GG | GG | GG | GG | GG | GG | GG | GG |
|  | GG | GG | GG | GG | GG | GG | GG | GG | GG | GG | GG | GG | GG |
|  | AA | AA | AA | AA | AA | AA | AA | AA | AA | AA | AA | AA | AA |
|  | GG | GG | GG | GG | GG | GG | GG | GG | GG | GG | GG | GG | GG |
|  | GG | GG | GG | GG | GG | GG | GG | GG | GG | GG | GG | GG | GG |
|  | TT | TT | TT | TT | TT | TT | TT | TT | TT | TT | TT | TT | TT |
|  | GG | GG | GG | GG | GG | GG | GG | GG | GG | GG | GG | GG | GG |
|  | GG | GG | GG | GG | GG | GG | GG | GG | GG | GG | GG | GG | GG |
|  | AA | AA | AA | AA | AA | AA | AA | AA | AA | AA | AA | AA | AA |
|  | GG | GG | GG | GG | GG | GG | GG | GG | GG | GG | GG | GG | GG |
|  | AA | AA | AA | AA | AA | AA | AA | AA | AA | AA | AA | AA | AA |
|  | CC | TT | TT | CC | CC | CC | CC | CC | CC | CC | CC | CC | CC |
|  | CC | TT | TT | CC | CC | CC | CC | CC | CC | CC | CC | CC | CC |
|  | TT | CC | CC | TT | TT | TT | TT | TT | TT | TT | TT | TT | TT |
|  | GG | AA | AA | GG | GG | GG | GG | GG | GG | GG | GG | GG | GG |
|  | TT | CC | CC | TT | TT | TT | TT | TT | TT | TT | TT | TT | TT |
|  | CC | AA | AA | CC | CC | CC | CC | CC | CC | CC | CC | CC | CC |
|  | CC | CC | CC | CC | CC | CC | CC | CC | CC | CC | CC | CC | CC |
|  | GG | GG | GG | GG | GG | GG | GG | GG | GG | GG | GG | GG | GG |
|  | GG | GG | GG | GG | GG | GG | GG | GG | GG | GG | GG | GG | GG |
|  | AA | AA | AA | AA | AA | AA | AA | AA | AA | AA | AA | AA | AA |
|  | CC | CC | CC | CC | CC | CC | CC | CC | CC | CC | CC | CC | CC |
|  | CC | CC | CC | CC | CC | CC | CC | CC | CC | CC | CC | CC | CC |
|  | CC | CC | CC | CC | CC | CC | CC | CC | CC | CC | CC | CC | CC |
|  | AA | AA | AA | AA | AA | AA | AA | AA | AA | AA | AA | AA | AA |
|  | AA | AA | AA | AA | AA | AA | AA | AA | AA | AA | AA | AA | AA |
|  | CC | CC | CC | CC | CC | CC | CC | CC | CC | CC | CC | CC | CC |
|  | AA | AA | AA | AA | AA | AA | AA | AA | AA | AA | AA | AA | AA |
|  | CC | CC | CC | CC | CC | CC | CC | CC | CC | CC | CC | CC | CC |
|  | AA | AA | AA | AA | AA | AA | AA | AA | AA | AA | AA | AA | AA |
|  | TT | TT | TT | TT | TT | TT | TT | TT | TT | TT | TT | TT | TT |
|  | AA | AA | AA | AA | AA | AA | AA | AA | AA | AA | AA | AA | AA |
|  | AA | AA | AA | AA | AA | AA | AA | AA | AA | AA | AA | AA | AA |
|  | GG | GG | GG | GG | GG | GG | GG | AA | AA | GG | GG | GG | GG |
|  | TT | TT | TT | TT | TT | TT | TT | AA | AA | TT | TT | TT | TT |
|  | GG | GG | GG | GG | GG | GG | GG | AA | AA | GG | GG | GG | GG |
|  | AA | AA | AA | AA | AA | AA | AA | GG | GG | AA | AA | AA | AA |
|  | CC | CC | CC | CC | CC | CC | CC | AA | AA | CC | CC | CC | CC |
|  | TT | TT | TT | TT | TT | TT | TT | AA | AA | TT | TT | TT | TT |
|  | GG | GG | GG | GG | GG | GG | GG | AA | AA | GG | GG | GG | GG |
|  | CC | CC | CC | CC | CC | CC | CC | AA | AA | CC | CC | CC | CC |
|  | GG | GG | GG | GG | GG | GG | GG | AA | AA | GG | GG | GG | GG |
|  | TT | TT | TT | TT | TT | TT | TT | TT | TT | TT | TT | TT | TT |
|  | CC | CC | CC | AA | AA | CC | CC | CC | CC | CC | CC | CC | CC |
|  | AA | AA | AA | GG | GG | AA | AA | AA | AA | AA | AA | AA | AA |
|  | AA | AA | AA | TT | TT | AA | AA | AA | AA | AA | AA | AA | AA |
|  | CC | CC | CC | TT | TT | CC | CC | CC | CC | CC | CC | CC | CC |
|  | TT | TT | TT | CC | CC | TT | TT | TT | TT | TT | TT | TT | TT |
|  | GG | GG | GG | AA | AA | GG | GG | GG | GG | GG | GG | GG | GG |
|  | AA | AA | AA | GG | GG | AA | AA | AA | AA | AA | AA | AA | AA |
|  | AA | AA | AA | TT | TT | AA | AA | AA | AA | AA | AA | AA | AA |
|  | GG | GG | GG | AA | AA | GG | GG | GG | GG | GG | GG | GG | GG |
|  | TT | TT | TT | CC | CC | TT | TT | TT | TT | TT | TT | TT | TT |
|  | CC | CC | CC | TT | TT | CC | CC | CC | CC | CC | CC | CC | CC |
|  | TT | TT | TT | CC | CC | TT | TT | TT | TT | TT | TT | TT | TT |
|  | GG | GG | GG | AA | AA | GG | GG | GG | GG | GG | GG | GG | GG |
|  | TT | TT | TT | TT | TT | TT | TT | TT | TT | TT | TT | TT | TT |
|  | AA | AA | AA | AA | AA | AA | AA | AA | AA | AA | AA | AA | AA |
|  | TT | TT | TT | TT | TT | TT | TT | TT | TT | TT | TT | TT | TT |
|  | CC | CC | CC | CC | CC | CC | CC | CC | CC | CC | CC | CC | CC |
|  | CC | CC | CC | CC | CC | CC | CC | CC | CC | CC | CC | CC | CC |
|  | TT | TT | TT | TT | TT | TT | TT | TT | TT | TT | TT | TT | TT |
|  | AA | AA | AA | AA | AA | AA | AA | AA | AA | AA | AA | AA | AA |
|  | GG | GG | GG | GG | GG | GG | GG | GG | GG | GG | GG | GG | GG |
|  | TT | TT | TT | TT | TT | TT | TT | TT | TT | TT | TT | TT | TT |
|  | CC | CC | CC | CC | CC | CC | CC | CC | CC | CC | CC | CC | CC |
|  | GG | GG | GG | GG | GG | GG | GG | GG | GG | GG | GG | GG | GG |
|  | AA | AA | AA | AA | AA | AA | AA | AA | AA | AA | AA | AA | AA |
|  | AA | AA | AA | AA | AA | AA | AA | AA | AA | AA | AA | AA | AA |
|  | AA | AA | AA | AA | AA | AA | AA | AA | AA | AA | AA | AA | AA |
|  | TT | TT | TT | TT | TT | TT | TT | TT | TT | TT | TT | TT | TT |
|  | GG | GG | GG | GG | GG | GG | GG | GG | GG | GG | GG | GG | GG |
|  | AA | AA | AA | AA | AA | AA | AA | AA | AA | AA | AA | AA | AA |
|  | CC | CC | CC | CC | CC | CC | CC | CC | CC | CC | CC | CC | CC |
|  | AA | AA | AA | AA | AA | AA | AA | AA | AA | AA | AA | AA | AA |
|  | CC | CC | CC | CC | CC | CC | CC | CC | CC | CC | CC | CC | CC |
|  | AA | AA | AA | AA | AA | AA | AA | AA | AA | AA | AA | AA | AA |
|  | AA | AA | AA | AA | AA | AA | AA | AA | AA | AA | AA | AA | AA |
|  | GG | GG | GG | GG | GG | GG | GG | GG | GG | GG | GG | GG | GG |
|  | AA | AA | AA | AA | AA | AA | AA | AA | AA | AA | AA | AA | AA |
|  | GG | GG | GG | GG | GG | GG | GG | GG | GG | GG | GG | GG | GG |
|  | GG | GG | GG | GG | GG | GG | GG | GG | GG | GG | GG | GG | GG |
|  | TT | TT | TT | TT | TT | TT | TT | TT | TT | TT | TT | TT | TT |
|  | AA | AA | AA | AA | AA | AA | AA | AA | AA | AA | AA | AA | AA |
|  | AA | AA | AA | AA | AA | AA | AA | AA | AA | AA | AA | AA | AA |
|  | GG | GG | GG | GG | GG | GG | GG | GG | GG | GG | GG | GG | GG |
|  | TT | TT | TT | TT | TT | TT | TT | TT | TT | TT | TT | TT | TT |
|  | CC | CC | CC | CC | CC | CC | CC | CC | CC | CC | CC | CC | CC |
|  | CC | CC | CC | CC | CC | CC | CC | CC | CC | CC | CC | CC | CC |
|  | TT | TT | TT | TT | TT | TT | TT | TT | TT | TT | TT | TT | TT |
|  | AA | AA | AA | AA | AA | AA | AA | AA | AA | AA | AA | AA | AA |
|  | AA | AA | AA | AA | AA | AA | AA | AA | AA | AA | AA | AA | AA |
|  | GG | GG | GG | GG | GG | GG | GG | GG | GG | GG | GG | GG | GG |
|  | AA | AA | AA | AA | AA | AA | AA | AA | AA | AA | AA | AA | AA |
|  | TT | TT | TT | TT | TT | TT | TT | TT | TT | TT | TT | TT | TT |
|  | TT | TT | TT | TT | TT | TT | TT | TT | TT | TT | TT | TT | TT |
|  | AA | AA | AA | AA | AA | AA | AA | AA | AA | AA | AA | AA | AA |
|  | TT | TT | TT | TT | TT | TT | TT | TT | TT | TT | TT | TT | TT |
|  | AA | AA | AA | AA | AA | AA | AA | AA | AA | AA | AA | AA | AA |
|  | AA | AA | AA | AA | AA | AA | AA | AA | AA | AA | AA | AA | AA |
|  | TT | TT | TT | TT | TT | TT | TT | TT | TT | TT | TT | TT | TT |
|  | GG | GG | GG | GG | GG | GG | GG | GG | GG | GG | GG | GG | GG |
|  | AA | AA | AA | AA | AA | AA | AA | AA | AA | AA | AA | AA | AA |
|  | GG | GG | GG | GG | GG | GG | GG | GG | GG | GG | GG | GG | GG |
|  | AA | AA | AA | AA | AA | AA | AA | AA | AA | AA | AA | AA | AA |
|  | CC | CC | CC | CC | CC | CC | CC | CC | CC | CC | CC | CC | CC |
|  | AA | AA | AA | AA | AA | AA | AA | AA | AA | AA | AA | AA | AA |
|  | CC | CC | CC | CC | CC | CC | CC | CC | CC | CC | CC | CC | CC |
|  | CC | CC | CC | CC | CC | CC | CC | CC | CC | CC | CC | CC | CC |
|  | CC | CC | CC | CC | CC | CC | CC | CC | CC | CC | CC | CC | CC |
|  | AA | AA | AA | AA | AA | AA | AA | AA | AA | AA | AA | AA | AA |
|  | TT | TT | TT | TT | TT | TT | TT | TT | TT | TT | TT | TT | TT |
|  | TT | TT | TT | TT | TT | TT | TT | TT | TT | TT | TT | TT | TT |
|  | TT | TT | TT | TT | TT | TT | TT | TT | TT | TT | TT | TT | TT |
|  | AA | AA | AA | AA | AA | AA | AA | AA | AA | AA | AA | AA | AA |
|  | AA | AA | AA | AA | AA | AA | AA | AA | AA | AA | AA | AA | AA |
|  | GG | GG | GG | GG | GG | GG | GG | GG | GG | GG | GG | GG | GG |
|  | CC | CC | CC | CC | CC | CC | CC | CC | CC | CC | CC | CC | CC |
|  | AA | AA | AA | AA | AA | AA | AA | AA | AA | AA | AA | AA | AA |
|  | AA | AA | AA | AA | AA | AA | AA | AA | AA | AA | AA | AA | AA |
|  | GG | GG | GG | GG | GG | GG | GG | GG | GG | GG | GG | GG | GG |
|  | AA | AA | AA | AA | AA | AA | AA | AA | AA | AA | AA | AA | AA |
|  | TT | TT | TT | TT | TT | TT | TT | TT | TT | TT | TT | TT | TT |
|  | TT | TT | TT | TT | TT | TT | TT | TT | TT | TT | TT | TT | TT |
|  | AA | AA | AA | AA | AA | AA | AA | AA | AA | AA | AA | AA | AA |
|  | AA | AA | AA | AA | AA | AA | AA | AA | AA | AA | AA | AA | AA |
|  | GG | GG | GG | GG | GG | GG | GG | GG | GG | GG | GG | GG | GG |
|  | AA | AA | AA | AA | AA | AA | AA | AA | AA | AA | AA | AA | AA |
|  | CC | CC | CC | CC | CC | CC | CC | CC | CC | CC | CC | CC | CC |
|  | GG | GG | GG | GG | GG | GG | GG | GG | GG | GG | GG | AA | AA |
|  | TT | TT | TT | TT | TT | TT | TT | TT | TT | TT | TT | CC | CC |
|  | AA | AA | AA | AA | AA | AA | AA | AA | AA | AA | AA | GG | GG |
|  | AA | AA | AA | AA | AA | AA | AA | AA | AA | AA | AA | CC | CC |
|  | CC | CC | CC | CC | CC | CC | CC | CC | CC | CC | CC | TT | TT |
|  | AA | AA | AA | AA | AA | AA | AA | AA | AA | AA | AA | GG | GG |
|  | AA | AA | AA | AA | AA | AA | AA | AA | AA | AA | AA | GG | GG |
|  | AA | AA | AA | AA | AA | AA | AA | AA | AA | AA | AA | CC | CC |
|  | TT | TT | TT | TT | TT | TT | TT | TT | TT | TT | TT | TT | TT |
|  | TT | TT | TT | TT | TT | TT | TT | TT | TT | TT | TT | TT | TT |
|  | AA | AA | AA | AA | AA | AA | AA | AA | AA | AA | AA | AA | AA |
|  | TT | TT | TT | TT | TT | TT | TT | TT | TT | TT | TT | TT | TT |
|  | CC | CC | CC | CC | CC | CC | CC | CC | CC | CC | CC | CC | CC |
|  | GG | GG | GG | GG | GG | GG | GG | GG | GG | GG | GG | GG | GG |
|  | AA | AA | AA | AA | AA | AA | AA | AA | AA | AA | AA | AA | AA |
|  | TT | TT | TT | TT | TT | TT | TT | TT | TT | TT | TT | TT | TT |
|  | AA | AA | AA | AA | AA | AA | AA | AA | AA | AA | AA | AA | AA |
|  | TT | TT | TT | TT | TT | TT | TT | TT | TT | TT | TT | TT | TT |
|  | AA | AA | AA | AA | AA | AA | AA | AA | AA | AA | AA | AA | AA |
|  | TT | TT | TT | TT | TT | TT | TT | TT | TT | TT | TT | TT | TT |
|  | CC | CC | CC | CC | CC | CC | CC | CC | CC | CC | CC | CC | CC |
|  | CC | CC | CC | CC | CC | CC | CC | CC | CC | CC | CC | CC | CC |
|  | AA | AA | AA | AA | AA | AA | AA | AA | AA | AA | AA | AA | AA |
|  | CC | CC | CC | CC | CC | CC | CC | CC | CC | CC | CC | CC | CC |
|  | TT | TT | TT | TT | TT | TT | TT | TT | TT | TT | TT | TT | TT |
|  | CC | CC | CC | CC | CC | CC | CC | CC | CC | CC | CC | CC | CC |
|  | TT | TT | TT | TT | TT | TT | TT | TT | TT | TT | TT | TT | TT |
|  | TT | TT | TT | TT | TT | TT | TT | TT | TT | TT | TT | TT | TT |
|  | TT | TT | TT | TT | TT | TT | TT | TT | TT | TT | TT | TT | TT |
|  | CC | CC | CC | CC | CC | CC | CC | CC | CC | CC | CC | CC | CC |
|  | TT | TT | TT | TT | TT | TT | TT | TT | TT | TT | TT | TT | TT |
|  | TT | TT | TT | TT | TT | TT | TT | TT | TT | TT | TT | TT | TT |
|  | GG | GG | GG | GG | GG | GG | GG | GG | GG | GG | GG | GG | GG |
|  | TT | TT | TT | TT | TT | TT | TT | TT | TT | TT | TT | TT | TT |
|  | CC | CC | CC | CC | CC | CC | CC | CC | CC | CC | CC | CC | CC |
|  | AA | AA | AA | AA | AA | AA | AA | AA | AA | AA | AA | AA | AA |
|  | AA | AA | AA | AA | AA | AA | AA | AA | AA | AA | AA | AA | AA |
|  | TT | TT | TT | TT | TT | TT | TT | TT | TT | TT | TT | TT | TT |
|  | CC | CC | CC | CC | CC | CC | CC | CC | CC | CC | CC | CC | CC |
|  | CC | CC | CC | CC | CC | CC | CC | CC | CC | CC | CC | CC | CC |
|  | AA | AA | AA | AA | AA | AA | AA | AA | AA | AA | AA | AA | AA |
|  | TT | TT | TT | TT | TT | TT | TT | TT | TT | TT | TT | TT | TT |
|  | AA | AA | AA | AA | AA | AA | AA | AA | AA | AA | AA | AA | AA |
|  | TT | TT | TT | TT | TT | TT | TT | TT | TT | TT | TT | TT | TT |
|  | CC | CC | CC | CC | CC | CC | CC | CC | CC | CC | CC | CC | CC |
|  | CC | CC | CC | CC | CC | CC | CC | CC | CC | CC | CC | CC | CC |
|  | TT | TT | TT | TT | TT | TT | TT | TT | TT | TT | TT | TT | TT |
|  | CC | CC | CC | CC | CC | CC | CC | CC | CC | CC | CC | CC | CC |
|  | AA | AA | AA | AA | AA | AA | AA | AA | AA | AA | AA | AA | AA |
|  | AA | AA | AA | AA | AA | AA | AA | AA | AA | AA | AA | AA | AA |
|  | GG | GG | GG | GG | GG | GG | GG | GG | GG | GG | GG | GG | GG |
|  | TT | TT | TT | TT | TT | TT | TT | TT | TT | TT | TT | TT | TT |
|  | TT | TT | TT | TT | TT | TT | TT | TT | TT | TT | TT | TT | TT |
|  | TT | TT | TT | TT | TT | TT | TT | TT | TT | TT | TT | TT | TT |
|  | TT | TT | TT | TT | TT | TT | TT | TT | TT | TT | TT | TT | TT |
|  | TT | TT | TT | TT | TT | TT | TT | TT | TT | TT | TT | TT | TT |
|  | GG | GG | GG | GG | GG | GG | GG | GG | GG | GG | GG | GG | GG |
|  | GG | GG | GG | GG | GG | GG | GG | GG | GG | GG | GG | GG | GG |
|  | AA | AA | AA | AA | AA | AA | AA | AA | AA | AA | AA | AA | AA |
|  | CC | CC | CC | CC | CC | CC | CC | CC | CC | CC | CC | CC | CC |
|  | AA | AA | AA | AA | AA | AA | AA | AA | AA | AA | AA | AA | AA |
|  | AA | AA | AA | AA | AA | AA | AA | AA | AA | AA | AA | AA | AA |
|  | CC | CC | CC | CC | CC | CC | CC | CC | CC | CC | CC | CC | CC |
|  | GG | GG | GG | GG | GG | GG | GG | GG | GG | GG | GG | GG | GG |
|  | CC | CC | CC | CC | CC | CC | CC | CC | CC | CC | CC | CC | CC |
|  | CC | CC | CC | CC | CC | CC | CC | CC | CC | CC | CC | CC | CC |
|  | GG | GG | GG | GG | GG | GG | GG | GG | GG | GG | GG | GG | GG |
|  | GG | GG | GG | GG | GG | GG | GG | GG | GG | GG | GG | GG | GG |
|  | CC | CC | CC | CC | CC | CC | CC | CC | CC | CC | CC | CC | CC |
|  | TT | TT | TT | TT | TT | TT | TT | TT | TT | TT | TT | TT | TT |
|  | AA | AA | AA | AA | AA | AA | AA | AA | AA | AA | AA | AA | AA |
|  | TT | TT | TT | TT | TT | TT | TT | TT | TT | TT | TT | TT | TT |
|  | CC | CC | CC | CC | CC | CC | CC | CC | CC | CC | CC | CC | CC |
|  | CC | CC | CC | CC | CC | CC | CC | CC | CC | CC | CC | CC | CC |
|  | GG | GG | GG | GG | GG | GG | GG | GG | GG | GG | GG | GG | GG |
|  | CC | CC | CC | CC | CC | CC | CC | CC | CC | CC | CC | CC | CC |
|  | AA | AA | AA | AA | AA | AA | AA | AA | AA | AA | AA | GG | GG |
|  | CC | CC | CC | CC | CC | CC | CC | CC | CC | CC | CC | TT | TT |
|  | GG | GG | GG | GG | GG | GG | GG | GG | GG | GG | GG | CC | CC |
|  | AA | AA | AA | AA | AA | AA | AA | AA | AA | AA | AA | GG | GG |
|  | GG | GG | GG | GG | GG | GG | GG | GG | GG | GG | GG | GG | GG |
|  | AA | AA | AA | AA | AA | AA | AA | AA | AA | AA | AA | AA | AA |
|  | TT | TT | TT | TT | TT | TT | TT | TT | TT | TT | TT | TT | TT |
|  | CC | CC | CC | CC | CC | CC | CC | CC | CC | CC | CC | CC | CC |
|  | GG | GG | GG | GG | GG | GG | GG | GG | GG | GG | GG | GG | GG |
|  | CC | CC | CC | CC | CC | CC | CC | CC | CC | CC | CC | CC | CC |
|  | AA | AA | AA | AA | AA | AA | AA | AA | AA | AA | AA | AA | AA |
|  | AA | AA | AA | AA | AA | AA | AA | AA | AA | AA | AA | AA | AA |
|  | AA | AA | AA | AA | AA | AA | AA | AA | AA | AA | AA | AA | AA |
|  | AA | AA | AA | AA | AA | AA | AA | AA | AA | AA | AA | AA | AA |
|  | GG | GG | GG | GG | GG | GG | GG | GG | GG | GG | GG | GG | GG |
|  | CC | CC | CC | CC | CC | CC | CC | CC | CC | CC | CC | CC | CC |
|  | CC | CC | CC | CC | CC | CC | CC | CC | CC | CC | CC | CC | CC |
|  | TT | TT | TT | TT | TT | TT | TT | TT | TT | TT | TT | TT | TT |
|  | GG | GG | GG | GG | GG | GG | GG | GG | GG | GG | GG | GG | GG |
|  | CC | CC | CC | CC | CC | CC | CC | CC | CC | CC | CC | CC | CC |
|  | GG | GG | GG | GG | GG | GG | GG | GG | GG | GG | GG | GG | GG |
|  | AA | AA | AA | AA | AA | AA | AA | AA | AA | AA | AA | AA | AA |
|  | GG | GG | GG | GG | GG | GG | GG | GG | GG | GG | GG | GG | GG |
|  | TT | TT | TT | TT | TT | TT | TT | TT | TT | TT | TT | TT | TT |
|  | GG | GG | GG | GG | GG | GG | GG | GG | GG | GG | GG | GG | GG |
|  | TT | TT | TT | TT | TT | TT | TT | TT | TT | TT | TT | TT | TT |
|  | AA | AA | AA | AA | AA | AA | AA | AA | AA | AA | AA | AA | AA |
|  | AA | AA | AA | AA | AA | AA | AA | AA | AA | AA | AA | AA | AA |
|  | TT | TT | TT | TT | TT | TT | TT | TT | TT | TT | TT | TT | TT |
|  | GG | GG | GG | GG | GG | GG | GG | GG | GG | GG | GG | GG | GG |
|  | GG | GG | GG | GG | GG | GG | GG | GG | GG | GG | GG | GG | GG |
|  | AA | AA | AA | AA | AA | AA | AA | AA | AA | AA | AA | AA | AA |
|  | GG | GG | GG | GG | GG | GG | GG | GG | GG | GG | GG | GG | GG |
|  | AA | AA | AA | AA | AA | AA | AA | AA | AA | AA | AA | AA | AA |
|  | AA | AA | AA | AA | AA | AA | AA | AA | AA | AA | AA | AA | AA |
|  | AA | AA | AA | AA | AA | AA | AA | AA | AA | AA | AA | AA | AA |
|  | AA | AA | AA | AA | AA | AA | AA | AA | AA | AA | AA | AA | AA |
|  | CC | CC | CC | CC | CC | CC | CC | CC | CC | CC | CC | CC | CC |
|  | CC | CC | CC | CC | CC | CC | CC | CC | CC | CC | CC | CC | CC |
|  | CC | CC | CC | CC | CC | CC | CC | CC | CC | CC | CC | CC | CC |
|  | AA | AA | AA | AA | AA | AA | AA | AA | AA | AA | AA | AA | AA |
|  | AA | AA | AA | AA | AA | AA | AA | AA | AA | AA | AA | AA | AA |
|  | GG | GG | GG | GG | GG | GG | GG | GG | GG | GG | GG | GG | GG |
|  | TT | TT | TT | TT | TT | TT | TT | TT | TT | TT | TT | TT | TT |
|  | TT | TT | TT | TT | TT | TT | TT | TT | TT | TT | TT | TT | TT |
|  | TT | TT | TT | TT | TT | TT | TT | TT | TT | TT | TT | TT | TT |
|  | TT | TT | TT | TT | TT | TT | TT | TT | TT | TT | TT | TT | TT |
|  | CC | CC | CC | CC | CC | CC | CC | CC | CC | CC | CC | CC | CC |
|  | AA | AA | AA | AA | AA | AA | AA | AA | AA | AA | AA | AA | AA |
|  | CC | CC | CC | CC | CC | CC | CC | CC | CC | CC | CC | CC | CC |
|  | GG | GG | GG | GG | GG | GG | GG | GG | GG | GG | GG | GG | GG |
|  | AA | AA | AA | AA | AA | AA | AA | AA | AA | AA | AA | AA | AA |
|  | GG | GG | GG | GG | GG | GG | GG | GG | GG | GG | GG | GG | GG |
|  | GG | GG | GG | GG | GG | GG | GG | GG | GG | GG | GG | GG | GG |
|  | GG | GG | GG | GG | GG | GG | GG | GG | GG | GG | GG | GG | GG |
|  | CC | CC | CC | CC | CC | CC | CC | CC | CC | CC | CC | CC | CC |
|  | AA | AA | AA | AA | AA | AA | AA | AA | AA | AA | AA | AA | AA |
|  | CC | CC | CC | CC | CC | CC | CC | CC | CC | CC | CC | CC | CC |
|  | AA | CC | CC | AA | AA | AA | AA | AA | AA | AA | AA | AA | AA |
|  | GG | AA | AA | GG | GG | GG | GG | GG | GG | GG | GG | GG | GG |
|  | TT | AA | AA | TT | TT | TT | TT | TT | TT | TT | TT | TT | TT |
|  | AA | TT | TT | AA | AA | AA | AA | AA | AA | AA | AA | AA | AA |
|  | TT | TT | TT | TT | TT | TT | TT | TT | TT | TT | TT | TT | TT |
|  | AA | AA | AA | AA | AA | AA | AA | AA | AA | AA | AA | AA | AA |
|  | CC | CC | CC | CC | CC | CC | CC | CC | CC | CC | CC | CC | CC |
|  | TT | TT | TT | TT | TT | TT | TT | TT | TT | TT | TT | TT | TT |
|  | AA | AA | AA | AA | AA | AA | AA | AA | AA | AA | AA | AA | AA |
|  | CC | CC | CC | CC | CC | CC | CC | CC | CC | CC | CC | CC | CC |
|  | TT | TT | TT | TT | TT | TT | TT | TT | TT | TT | TT | TT | TT |
|  | GG | GG | GG | GG | GG | GG | GG | GG | GG | GG | GG | GG | GG |
|  | AA | AA | AA | AA | AA | AA | AA | TT | TT | AA | AA | AA | AA |
|  | TT | TT | TT | TT | TT | TT | TT | GG | GG | TT | TT | TT | TT |
|  | GG | GG | GG | GG | GG | GG | GG | AA | AA | GG | GG | GG | GG |
|  | AA | AA | AA | AA | AA | AA | AA | GG | GG | AA | AA | AA | AA |
|  | CC | CC | CC | CC | CC | CC | CC | GG | GG | CC | CC | CC | CC |
|  | GG | GG | GG | GG | GG | GG | GG | AA | AA | GG | GG | GG | GG |
|  | GG | GG | GG | GG | GG | GG | GG | AA | AA | GG | GG | GG | GG |
|  | AA | AA | AA | AA | AA | AA | AA | GG | GG | AA | AA | AA | AA |
|  | CC | CC | CC | CC | CC | CC | CC | CC | CC | CC | CC | CC | CC |
|  | GG | GG | GG | GG | GG | GG | GG | GG | GG | GG | GG | GG | GG |
|  | GG | GG | GG | GG | GG | GG | GG | GG | GG | GG | GG | GG | GG |
|  | AA | AA | AA | AA | AA | AA | AA | AA | AA | AA | AA | AA | AA |
|  | GG | GG | GG | GG | GG | GG | GG | GG | GG | GG | GG | GG | GG |
|  | GG | GG | GG | GG | GG | GG | GG | GG | GG | GG | GG | CC | CC |
|  | TT | TT | TT | TT | TT | TT | TT | TT | TT | TT | TT | GG | GG |
|  | TT | TT | TT | TT | TT | TT | TT | TT | TT | TT | TT | GG | GG |
|  | GG | GG | GG | GG | GG | GG | GG | GG | GG | GG | GG | AA | AA |
|  | TT | TT | TT | TT | TT | TT | TT | TT | TT | TT | TT | TT | TT |
|  | GG | GG | GG | GG | GG | GG | GG | GG | GG | GG | GG | GG | GG |
|  | AA | AA | AA | AA | AA | AA | AA | AA | AA | AA | AA | AA | AA |
|  | CC | CC | CC | CC | CC | CC | CC | CC | CC | CC | CC | CC | CC |
|  | TT | TT | TT | TT | TT | TT | TT | TT | TT | TT | TT | TT | TT |
|  | TT | TT | TT | TT | TT | TT | TT | TT | TT | TT | TT | TT | TT |
|  | AA | AA | AA | AA | AA | AA | AA | AA | AA | AA | AA | AA | AA |
|  | CC | CC | CC | CC | CC | CC | CC | CC | CC | CC | CC | CC | CC |
|  | AA | AA | AA | AA | AA | AA | AA | AA | AA | AA | AA | AA | AA |
|  | TT | TT | TT | TT | TT | TT | TT | TT | TT | TT | TT | TT | TT |
|  | CC | CC | CC | CC | CC | CC | CC | CC | CC | CC | CC | CC | CC |
|  | CC | CC | CC | CC | CC | CC | CC | CC | CC | CC | CC | CC | CC |
|  | GG | GG | GG | GG | GG | GG | GG | GG | GG | GG | GG | GG | GG |
|  | CC | CC | CC | CC | CC | CC | CC | CC | CC | CC | CC | CC | CC |
|  | CC | CC | CC | CC | CC | CC | CC | CC | CC | CC | CC | CC | CC |
|  | AA | AA | AA | AA | AA | AA | AA | AA | AA | AA | AA | AA | AA |
|  | GG | GG | GG | GG | GG | GG | GG | GG | GG | GG | GG | GG | GG |
|  | GG | GG | GG | GG | GG | GG | GG | GG | GG | GG | GG | GG | GG |
|  | CC | CC | CC | CC | CC | CC | CC | CC | CC | CC | CC | CC | CC |
|  | CC | CC | CC | CC | CC | CC | CC | CC | CC | CC | CC | CC | CC |
|  | AA | AA | AA | AA | AA | AA | AA | AA | AA | AA | AA | AA | AA |
|  | CC | CC | CC | CC | CC | CC | CC | CC | CC | CC | CC | CC | CC |
|  | TT | TT | TT | TT | TT | TT | TT | TT | TT | TT | TT | TT | TT |
|  | GG | GG | GG | GG | GG | GG | GG | GG | GG | GG | GG | GG | GG |
|  | CC | CC | CC | CC | CC | CC | CC | CC | CC | CC | CC | CC | CC |
|  | AA | AA | AA | AA | AA | AA | AA | AA | AA | AA | AA | AA | AA |
|  | CC | CC | CC | CC | CC | CC | CC | CC | CC | CC | CC | CC | CC |
|  | AA | AA | AA | AA | AA | AA | AA | AA | AA | AA | AA | AA | AA |
|  | TT | TT | TT | TT | TT | TT | TT | TT | TT | TT | TT | TT | TT |
|  | GG | GG | GG | GG | GG | GG | GG | GG | GG | GG | GG | GG | GG |
|  | TT | TT | TT | TT | TT | TT | TT | TT | TT | TT | TT | TT | TT |
|  | CC | CC | CC | CC | CC | CC | CC | CC | CC | CC | CC | CC | CC |
|  | TT | TT | TT | TT | TT | TT | TT | TT | TT | TT | TT | TT | TT |
|  | CC | CC | CC | CC | CC | CC | CC | CC | CC | CC | CC | CC | CC |
|  | AA | AA | AA | AA | AA | AA | AA | AA | AA | AA | AA | AA | AA |
|  | TT | TT | TT | TT | TT | TT | TT | TT | TT | TT | TT | TT | TT |
|  | CC | CC | CC | CC | CC | CC | CC | CC | CC | CC | CC | CC | CC |
|  | CC | CC | CC | CC | CC | CC | CC | CC | CC | CC | CC | CC | CC |
|  | GG | GG | GG | GG | GG | GG | GG | GG | GG | GG | GG | GG | GG |
|  | AA | AA | AA | AA | AA | AA | AA | AA | AA | AA | AA | AA | AA |
|  | TT | TT | TT | TT | TT | TT | TT | TT | TT | TT | TT | TT | TT |
|  | CC | CC | CC | CC | CC | CC | CC | CC | CC | CC | CC | CC | CC |
|  | GG | GG | GG | GG | GG | GG | GG | GG | GG | GG | GG | GG | GG |
|  | TT | TT | TT | TT | TT | TT | TT | TT | TT | TT | TT | TT | TT |
|  | TT | TT | TT | TT | TT | TT | TT | TT | TT | TT | TT | TT | TT |
|  | TT | TT | TT | TT | TT | TT | TT | TT | TT | TT | TT | TT | TT |
|  | GG | GG | GG | GG | GG | GG | GG | GG | GG | GG | GG | GG | GG |
|  | GG | GG | GG | GG | GG | GG | GG | GG | GG | GG | GG | GG | GG |
|  | GG | GG | GG | GG | GG | GG | GG | GG | GG | GG | GG | GG | GG |
|  | GG | GG | GG | GG | GG | GG | GG | GG | GG | GG | GG | GG | GG |
|  | AA | AA | AA | AA | AA | AA | AA | AA | AA | AA | AA | AA | AA |
|  | CC | CC | CC | CC | CC | CC | CC | CC | CC | CC | CC | CC | CC |
|  | GG | GG | GG | GG | GG | GG | GG | GG | GG | GG | GG | GG | GG |
|  | AA | AA | AA | AA | AA | AA | AA | AA | AA | AA | AA | AA | AA |
|  | AA | AA | AA | AA | AA | AA | AA | AA | AA | AA | AA | AA | AA |
|  | AA | AA | AA | AA | AA | AA | AA | AA | AA | AA | AA | AA | AA |
|  | AA | AA | AA | AA | AA | AA | AA | AA | AA | AA | AA | AA | AA |
|  | CC | CC | CC | CC | CC | CC | CC | CC | CC | CC | CC | CC | CC |
|  | TT | TT | TT | TT | TT | TT | TT | TT | TT | TT | TT | TT | TT |
|  | CC | CC | CC | CC | CC | CC | CC | CC | CC | CC | CC | CC | CC |
|  | CC | CC | CC | CC | CC | CC | CC | CC | CC | CC | CC | CC | CC |
|  | CC | CC | CC | CC | CC | CC | CC | CC | CC | CC | CC | CC | CC |
|  | TT | TT | TT | TT | TT | TT | TT | TT | TT | TT | TT | TT | TT |
|  | AA | AA | AA | AA | AA | AA | AA | AA | AA | AA | AA | AA | AA |
|  | AA | AA | AA | AA | AA | AA | AA | AA | AA | AA | AA | AA | AA |
|  | TT | TT | TT | TT | TT | TT | TT | TT | TT | TT | TT | TT | TT |
|  | CC | CC | CC | CC | CC | CC | CC | CC | CC | CC | CC | CC | CC |
|  | GG | GG | GG | GG | GG | GG | GG | GG | GG | GG | GG | GG | GG |
|  | TT | TT | TT | TT | TT | TT | TT | TT | TT | TT | TT | TT | TT |
|  | GG | GG | GG | GG | GG | GG | GG | GG | GG | GG | GG | GG | GG |
|  | CC | CC | CC | CC | CC | CC | CC | CC | CC | CC | CC | CC | CC |
|  | GG | GG | GG | GG | GG | GG | GG | GG | GG | GG | GG | GG | GG |
|  | CC | CC | CC | CC | CC | CC | CC | CC | CC | CC | CC | CC | CC |
|  | GG | GG | GG | GG | GG | GG | GG | GG | GG | GG | GG | GG | GG |
|  | CC | CC | CC | CC | CC | CC | CC | CC | CC | CC | CC | CC | CC |
|  | TT | TT | TT | TT | TT | TT | TT | TT | TT | TT | TT | TT | TT |
|  | AA | AA | AA | AA | AA | AA | AA | AA | AA | AA | AA | AA | AA |
|  | TT | TT | TT | TT | TT | TT | TT | TT | TT | TT | TT | TT | TT |
|  | CC | CC | CC | CC | CC | CC | CC | CC | CC | CC | CC | CC | CC |
|  | GG | GG | GG | GG | GG | GG | GG | GG | GG | GG | GG | GG | GG |
|  | AA | AA | AA | AA | AA | AA | AA | AA | AA | AA | AA | AA | AA |
|  | GG | GG | GG | GG | GG | GG | GG | GG | GG | GG | GG | GG | GG |
|  | AA | AA | AA | AA | AA | AA | AA | AA | AA | AA | AA | AA | AA |
|  | GG | GG | GG | GG | GG | GG | GG | GG | GG | GG | GG | GG | GG |
|  | CC | CC | CC | CC | CC | CC | CC | CC | CC | CC | CC | CC | CC |
|  | GG | GG | GG | GG | GG | GG | GG | GG | GG | GG | GG | GG | GG |
|  | AA | AA | AA | AA | AA | AA | AA | AA | AA | AA | AA | AA | AA |
|  | TT | TT | TT | TT | TT | TT | TT | TT | TT | TT | TT | TT | TT |
|  | GG | GG | GG | GG | GG | GG | GG | GG | GG | GG | GG | GG | GG |
|  | GG | GG | GG | GG | GG | GG | GG | GG | GG | GG | GG | GG | GG |
|  | CC | CC | CC | CC | CC | CC | CC | CC | CC | CC | CC | CC | CC |
|  | GG | GG | GG | GG | GG | GG | GG | GG | GG | GG | GG | GG | GG |
|  | GG | GG | GG | GG | GG | GG | GG | GG | GG | GG | GG | GG | GG |
|  | TT | TT | TT | TT | TT | TT | TT | TT | TT | TT | TT | TT | TT |
|  | GG | GG | GG | GG | GG | GG | GG | GG | GG | GG | GG | GG | GG |
|  | TT | TT | TT | TT | TT | TT | TT | TT | TT | TT | TT | TT | TT |
|  | GG | GG | GG | GG | GG | GG | GG | GG | GG | GG | GG | GG | GG |
|  | AA | AA | AA | AA | AA | AA | AA | AA | AA | AA | AA | AA | AA |
|  | GG | GG | GG | GG | GG | GG | GG | GG | GG | GG | GG | GG | GG |
|  | TT | TT | TT | TT | TT | TT | TT | TT | TT | TT | TT | TT | TT |
|  | TT | TT | TT | TT | TT | TT | TT | TT | TT | TT | TT | TT | TT |
|  | TT | TT | TT | TT | TT | TT | TT | TT | TT | TT | TT | TT | TT |
|  | TT | TT | TT | TT | TT | TT | TT | TT | TT | TT | TT | TT | TT |
|  | TT | TT | TT | TT | TT | TT | TT | TT | TT | TT | TT | TT | TT |
|  | TT | TT | TT | TT | TT | TT | TT | TT | TT | TT | TT | TT | TT |
|  | AA | AA | AA | AA | AA | AA | AA | AA | AA | AA | AA | AA | AA |
|  | TT | TT | TT | TT | TT | TT | TT | TT | TT | TT | TT | TT | TT |
|  | AA | AA | AA | AA | AA | AA | AA | AA | AA | AA | AA | AA | AA |
|  | AA | AA | AA | AA | AA | AA | AA | AA | AA | AA | AA | AA | AA |
|  | TT | TT | TT | TT | TT | TT | TT | TT | TT | TT | TT | TT | TT |
|  | CC | CC | CC | CC | CC | CC | CC | CC | CC | CC | CC | CC | CC |
|  | AA | AA | AA | AA | AA | AA | AA | AA | AA | AA | AA | AA | AA |
|  | GG | GG | GG | GG | GG | GG | GG | GG | GG | GG | GG | GG | GG |
|  | GG | GG | GG | GG | GG | GG | GG | GG | GG | GG | GG | GG | GG |
|  | TT | TT | TT | TT | TT | TT | TT | TT | TT | TT | TT | TT | TT |
|  | GG | GG | GG | GG | GG | GG | GG | GG | GG | GG | GG | GG | GG |
|  | AA | AA | AA | AA | AA | AA | AA | AA | AA | AA | AA | AA | AA |
|  | AA | AA | AA | AA | AA | AA | AA | AA | AA | AA | AA | AA | AA |
|  | CC | CC | CC | CC | CC | CC | CC | CC | CC | CC | CC | CC | CC |
|  | CC | CC | CC | CC | CC | CC | CC | CC | CC | CC | CC | CC | CC |
|  | AA | AA | AA | AA | AA | AA | AA | AA | AA | AA | AA | AA | AA |
|  | AA | AA | AA | AA | AA | AA | AA | AA | AA | AA | AA | AA | AA |
|  | GG | GG | GG | GG | GG | GG | GG | GG | GG | GG | GG | GG | GG |
|  | TT | TT | TT | TT | TT | TT | TT | TT | TT | TT | TT | TT | TT |
|  | TT | TT | TT | TT | TT | TT | TT | TT | TT | TT | TT | TT | TT |
|  | TT | TT | TT | TT | TT | TT | TT | TT | TT | TT | TT | TT | TT |
|  | CC | CC | CC | CC | CC | CC | CC | CC | CC | CC | CC | CC | CC |
|  | AA | AA | AA | AA | AA | AA | AA | AA | AA | AA | AA | AA | AA |
|  | CC | CC | CC | CC | CC | CC | CC | CC | CC | CC | CC | CC | CC |
|  | AA | AA | AA | AA | AA | AA | AA | AA | AA | AA | AA | AA | AA |
|  | AA | AA | AA | AA | AA | AA | AA | AA | AA | AA | AA | AA | AA |
|  | AA | AA | AA | AA | AA | AA | AA | AA | AA | AA | AA | AA | AA |
|  | GG | GG | GG | GG | GG | GG | GG | GG | GG | GG | GG | GG | GG |
|  | GG | GG | GG | GG | GG | GG | GG | GG | GG | GG | GG | GG | GG |
|  | CC | CC | CC | CC | CC | CC | CC | CC | CC | CC | CC | CC | CC |
|  | CC | CC | CC | CC | CC | CC | CC | CC | CC | CC | CC | CC | CC |
|  | TT | TT | TT | TT | TT | TT | TT | TT | TT | TT | TT | TT | TT |
|  | CC | CC | CC | CC | CC | CC | CC | CC | CC | CC | CC | CC | CC |
|  | GG | GG | GG | GG | GG | GG | GG | GG | GG | GG | GG | GG | GG |
|  | TT | TT | TT | TT | TT | TT | TT | TT | TT | TT | TT | TT | TT |
|  | CC | CC | CC | CC | CC | CC | CC | CC | CC | CC | CC | CC | CC |
|  | GG | GG | GG | GG | GG | GG | GG | GG | GG | GG | GG | GG | GG |
|  | CC | CC | CC | CC | CC | CC | CC | CC | CC | CC | CC | CC | CC |
|  | GG | GG | GG | GG | GG | GG | GG | GG | GG | GG | GG | GG | GG |
|  | AA | AA | AA | AA | AA | AA | AA | AA | AA | AA | AA | AA | AA |
|  | AA | AA | AA | AA | AA | AA | AA | AA | AA | AA | AA | AA | AA |
|  | GG | GG | GG | GG | GG | GG | GG | GG | GG | GG | GG | GG | GG |
|  | GG | GG | GG | GG | GG | GG | GG | GG | GG | GG | GG | GG | GG |
|  | GG | GG | GG | GG | GG | GG | GG | GG | GG | GG | GG | GG | GG |
|  | AA | AA | AA | AA | AA | AA | AA | AA | AA | AA | AA | AA | AA |
|  | TT | TT | TT | TT | TT | TT | TT | TT | TT | TT | TT | TT | TT |
|  | GG | GG | GG | GG | GG | GG | GG | GG | GG | GG | GG | GG | GG |
|  | TT | TT | TT | TT | TT | TT | TT | TT | TT | TT | TT | TT | TT |
|  | AA | AA | AA | AA | AA | AA | AA | AA | AA | AA | AA | AA | AA |
|  | CC | CC | CC | CC | CC | CC | CC | CC | CC | CC | CC | CC | CC |
|  | CC | CC | CC | CC | CC | CC | CC | CC | CC | CC | CC | CC | CC |
|  | AA | AA | AA | AA | AA | AA | AA | AA | AA | AA | AA | AA | AA |
|  | AA | AA | AA | AA | AA | AA | AA | AA | AA | AA | AA | AA | AA |
|  | TT | TT | TT | TT | TT | TT | TT | TT | TT | TT | TT | TT | TT |
|  | AA | AA | AA | AA | AA | AA | AA | AA | AA | AA | AA | AA | AA |
|  | GG | GG | GG | GG | GG | GG | GG | GG | GG | GG | GG | GG | GG |
|  | AA | AA | AA | AA | AA | AA | AA | AA | AA | AA | AA | AA | AA |
|  | TT | TT | TT | TT | TT | TT | TT | TT | TT | TT | TT | TT | TT |
|  | TT | TT | TT | TT | TT | TT | TT | TT | TT | TT | TT | TT | TT |
|  | CC | CC | CC | CC | CC | CC | CC | CC | CC | CC | CC | CC | CC |
|  | GG | GG | GG | GG | GG | GG | GG | GG | GG | GG | GG | GG | GG |
|  | AA | AA | AA | AA | AA | AA | AA | AA | AA | AA | AA | AA | AA |
|  | TT | TT | TT | TT | TT | TT | TT | TT | TT | TT | TT | TT | TT |
|  | GG | GG | GG | GG | GG | GG | GG | GG | GG | GG | GG | GG | GG |
|  | AA | AA | AA | AA | AA | AA | AA | AA | AA | AA | AA | AA | AA |
|  | AA | AA | AA | AA | AA | AA | AA | AA | AA | AA | AA | AA | AA |
|  | GG | GG | GG | GG | GG | GG | GG | GG | GG | GG | GG | GG | GG |
|  | TT | TT | TT | TT | TT | TT | TT | TT | TT | TT | TT | TT | TT |
|  | AA | AA | AA | AA | AA | AA | AA | AA | AA | AA | AA | AA | AA |
|  | CC | CC | CC | CC | CC | CC | CC | CC | CC | CC | CC | CC | CC |
|  | AA | AA | AA | AA | AA | AA | AA | AA | AA | AA | AA | AA | AA |
|  | CC | CC | CC | CC | CC | CC | CC | CC | CC | CC | CC | CC | CC |
|  | CC | CC | CC | CC | CC | CC | CC | CC | CC | CC | CC | CC | CC |
|  | TT | TT | TT | TT | TT | TT | TT | TT | TT | TT | TT | TT | TT |
|  | CC | CC | CC | CC | CC | CC | CC | CC | CC | CC | CC | CC | CC |
|  | TT | TT | TT | TT | TT | TT | TT | TT | TT | TT | TT | TT | TT |
|  | AA | AA | AA | AA | AA | AA | AA | AA | AA | AA | AA | AA | AA |
|  | CC | CC | CC | CC | CC | CC | CC | CC | CC | CC | CC | CC | CC |
|  | AA | AA | AA | AA | AA | AA | AA | AA | AA | AA | AA | AA | AA |
|  | GG | GG | GG | GG | GG | GG | GG | GG | GG | GG | GG | GG | GG |
|  | TT | TT | TT | TT | TT | TT | TT | TT | TT | TT | TT | TT | TT |
|  | TT | TT | TT | TT | TT | TT | TT | TT | TT | TT | TT | TT | TT |
|  | CC | CC | CC | CC | CC | CC | CC | CC | CC | CC | CC | CC | CC |
|  | TT | TT | TT | TT | TT | TT | TT | TT | TT | TT | TT | TT | TT |
|  | GG | GG | GG | GG | GG | GG | GG | GG | GG | GG | GG | GG | GG |
|  | AA | AA | AA | AA | AA | AA | AA | AA | AA | AA | AA | AA | AA |
|  | AA | AA | AA | AA | AA | AA | AA | AA | AA | AA | AA | AA | AA |
|  | GG | GG | GG | GG | GG | GG | GG | GG | GG | GG | GG | GG | GG |
|  | CC | CC | CC | CC | CC | CC | CC | CC | CC | CC | CC | CC | CC |
|  | GG | GG | GG | GG | GG | GG | GG | GG | GG | GG | GG | GG | GG |
|  | TT | TT | TT | TT | TT | TT | TT | TT | TT | TT | TT | TT | TT |
|  | CC | CC | CC | CC | CC | CC | CC | CC | CC | CC | CC | CC | CC |
|  | TT | TT | TT | TT | TT | TT | TT | TT | TT | TT | TT | TT | TT |
|  | GG | GG | GG | GG | GG | GG | GG | GG | GG | GG | GG | GG | GG |
|  | GG | GG | GG | GG | GG | GG | GG | GG | GG | GG | GG | GG | GG |
|  | TT | TT | TT | TT | TT | TT | TT | TT | TT | TT | TT | TT | TT |
|  | AA | AA | AA | AA | AA | AA | AA | AA | AA | AA | AA | AA | AA |
|  | AA | AA | AA | AA | AA | AA | AA | AA | AA | AA | AA | AA | AA |
|  | CC | CC | CC | CC | CC | CC | CC | CC | CC | CC | CC | CC | CC |
|  | AA | AA | AA | AA | AA | AA | AA | AA | AA | AA | AA | AA | AA |
|  | GG | GG | GG | GG | GG | GG | GG | GG | GG | GG | GG | GG | GG |
|  | AA | AA | AA | AA | AA | AA | AA | AA | AA | AA | AA | AA | AA |
|  | CC | CC | CC | CC | CC | CC | CC | CC | CC | CC | CC | CC | CC |
|  | AA | AA | AA | AA | AA | AA | AA | AA | AA | AA | AA | AA | AA |
|  | TT | TT | TT | TT | TT | TT | TT | TT | TT | TT | TT | TT | TT |
|  | AA | AA | AA | AA | AA | AA | AA | AA | AA | AA | AA | AA | AA |
|  | AA | AA | AA | AA | AA | AA | AA | AA | AA | AA | AA | AA | AA |
|  | AA | AA | AA | AA | AA | AA | AA | AA | AA | AA | AA | AA | AA |
|  | TT | TT | TT | TT | TT | TT | TT | TT | TT | TT | TT | TT | TT |
|  | CC | CC | CC | CC | CC | CC | CC | CC | CC | CC | CC | CC | CC |
|  | GG | GG | GG | GG | GG | GG | GG | GG | GG | GG | GG | GG | GG |
|  | AA | AA | AA | AA | AA | AA | AA | AA | AA | AA | AA | AA | AA |
|  | TT | TT | TT | TT | TT | TT | TT | TT | TT | TT | TT | TT | TT |
|  | CC | CC | CC | CC | CC | CC | CC | CC | CC | CC | CC | CC | CC |
|  | AA | AA | AA | AA | AA | AA | AA | AA | AA | AA | AA | AA | AA |
|  | CC | CC | CC | CC | CC | CC | CC | CC | CC | CC | CC | CC | CC |
|  | GG | GG | GG | GG | GG | GG | GG | GG | GG | GG | GG | GG | GG |
|  | GG | GG | GG | GG | GG | GG | GG | GG | GG | GG | GG | GG | GG |
|  | CC | CC | CC | CC | CC | CC | CC | CC | CC | CC | CC | CC | CC |
|  | TT | TT | TT | TT | TT | TT | TT | TT | TT | TT | TT | TT | TT |
|  | GG | GG | GG | GG | GG | GG | GG | GG | GG | GG | GG | GG | GG |
|  | TT | TT | TT | TT | TT | TT | TT | TT | TT | TT | TT | TT | TT |
|  | CC | CC | CC | CC | CC | CC | CC | CC | CC | CC | CC | CC | CC |
|  | AA | AA | AA | AA | AA | AA | AA | AA | AA | AA | AA | AA | AA |
|  | AA | AA | AA | AA | AA | AA | AA | AA | AA | AA | AA | AA | AA |
|  | TT | TT | TT | TT | TT | TT | TT | TT | TT | TT | TT | TT | TT |
|  | GG | GG | GG | GG | GG | GG | GG | GG | GG | GG | GG | GG | GG |
|  | AA | AA | AA | AA | AA | AA | AA | AA | AA | AA | AA | AA | AA |
|  | GG | GG | GG | GG | GG | GG | GG | GG | GG | GG | GG | GG | GG |
|  | AA | AA | AA | AA | AA | AA | AA | AA | AA | AA | AA | AA | AA |
|  | TT | TT | TT | TT | TT | TT | TT | TT | TT | TT | TT | TT | TT |
|  | TT | TT | TT | TT | TT | TT | TT | TT | TT | TT | TT | TT | TT |
|  | TT | TT | TT | TT | TT | TT | TT | TT | TT | TT | TT | TT | TT |
